# Enzymatic Fluoromethylation Enabled by the *S*-Adenosylmethionine Analog *Te*-Adenosyl-*L*-(fluoromethyl)homotellurocysteine

**DOI:** 10.1021/acscentsci.2c01385

**Published:** 2023-05-08

**Authors:** Syam Sundar Neti, Bo Wang, David F. Iwig, Elizabeth L. Onderko, Squire J. Booker

**Affiliations:** ^†^Department of Chemistry, ^‡^Department of Biochemistry and Molecular Biology, and ^§^Howard Hughes Medical Institute, The Pennsylvania State University, University Park, Pennsylvania 16802, United States

## Abstract

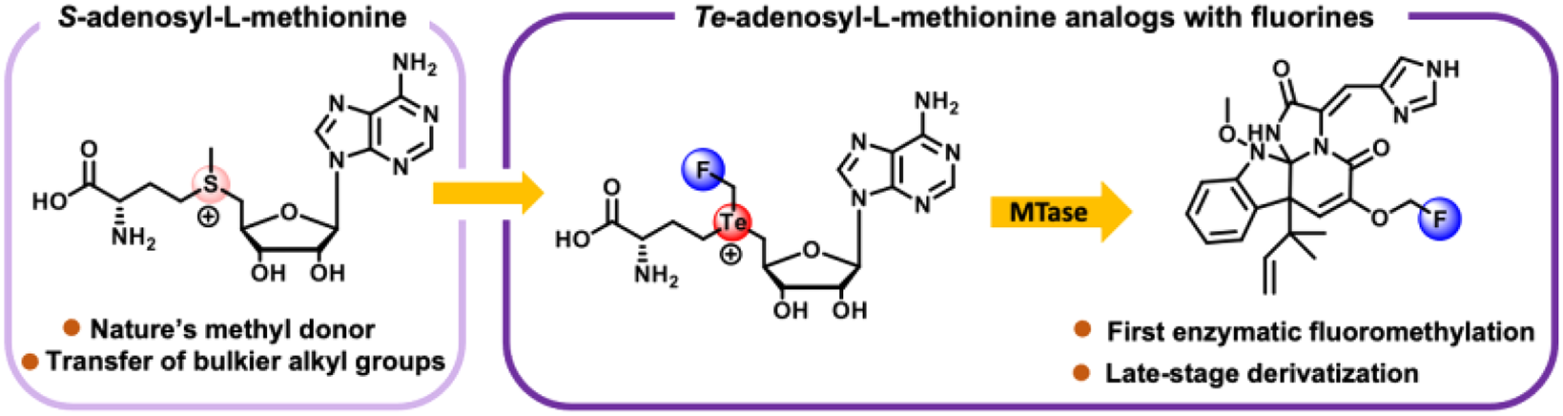

Fluoromethyl, difluoromethyl, and trifluoromethyl groups are present in numerous pharmaceuticals and agrochemicals, where they play critical roles in the efficacy and metabolic stability of these molecules. Strategies for late-stage incorporation of fluorine-containing atoms in molecules have become an important area of organic and medicinal chemistry as well as synthetic biology. Herein, we describe the synthesis and use of *Te*-adenosyl-*L*-(fluoromethyl)homotellurocysteine (FMeTeSAM), a novel and biologically relevant fluoromethylating agent. FMeTeSAM is structurally and chemically related to the universal cellular methyl donor *S*-adenosyl-*L*-methionine (SAM) and supports the robust transfer of fluoromethyl groups to oxygen, nitrogen, sulfur, and some carbon nucleophiles. FMeTeSAM is also used to fluoromethylate precursors to oxaline and daunorubicin, two complex natural products that exhibit antitumor properties.

## Abstract 2

FMeTeSAM, an analog of SAM containing a fluoromethyl group, was chemoenzymatically synthesized and used to study enzymatic fluoromethyl transfer on a variety of nucleophilic substrates.

## Introduction

**Figure 1:**
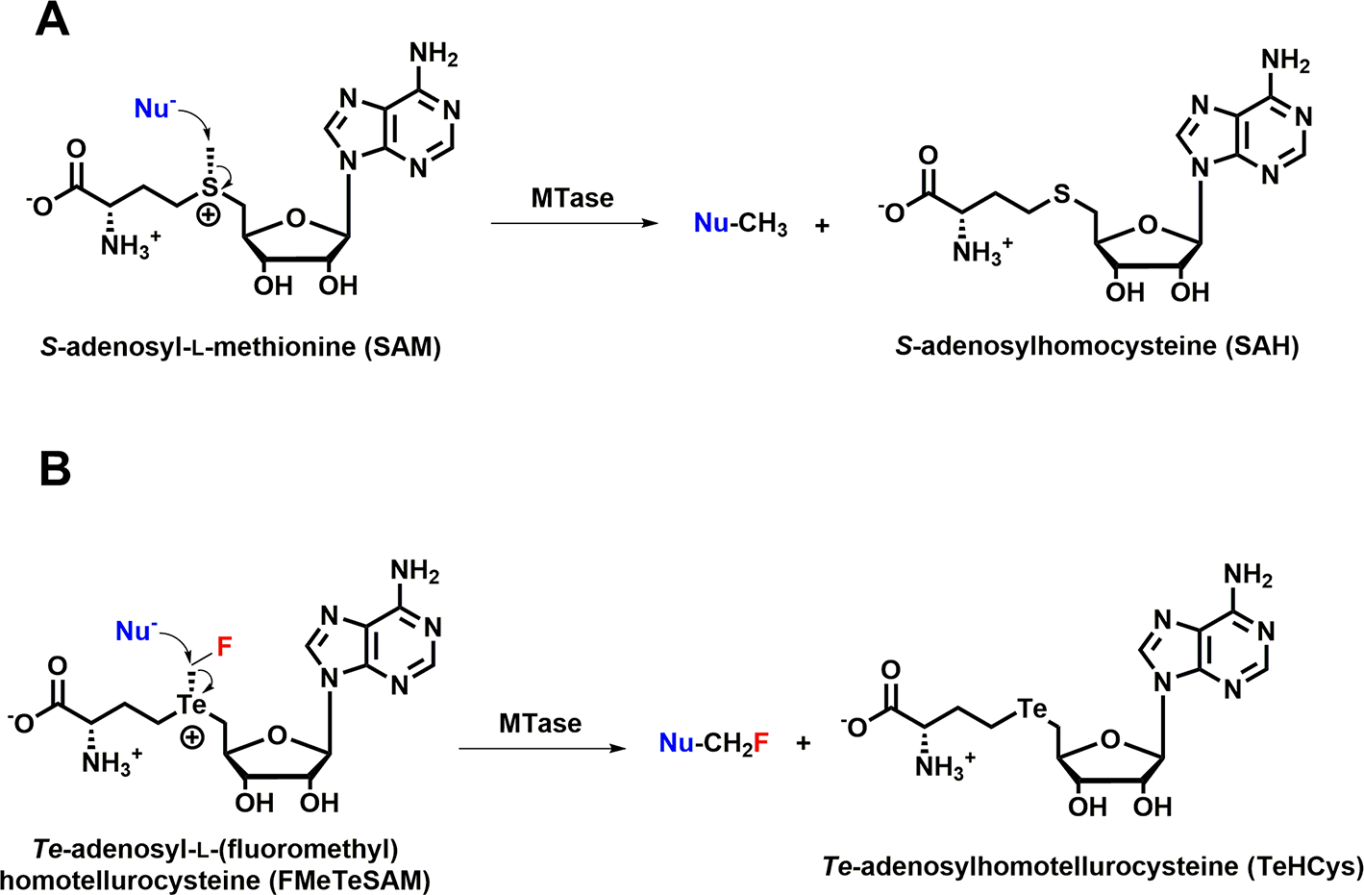
MTase-catalyzed methylation using (A) SAM and fluoromethylation using (B) FMeTeSAM.

Methylation underpins myriad cellular processes central to life, such as transcription, translation, gene regulation, signaling, and general metabolism.^1−7^*S*-Adenosyl-*L*-methionine (SAM or AdoMet), often referred to as Nature’s universal methylating agent, is the overwhelmingly predominant source of the appended methyl groups.^8,9^ Enzymes use SAM to methylate numerous biomolecules, including DNA, RNA, proteins, lipids, carbohydrates, and a wide variety of small molecules.^5,10−19^ The canonical reaction involves a polar S_N_2 attack of a nucleophile onto the electrophilic methyl substituent of SAM, affording *S*-adenosyl-*L*-homocysteine (SAH) as a coproduct (Figure 1A).^20,21^ The most widely methylated nucleophiles are N, O, and C (where carbanions can be generated). However, P, S, Se, Te, As, Co (in cobalamin), and Hg also receive methyl groups from SAM.^10,11,22−26^ SAM-dependent methyltransferases (MTases) are ubiquitous in nature, and a variety of them have been shown *in vitro* to have broad substrate scopes.^27^

The introduction of alkyl groups—especially methyl groups—is a well-known strategy in the pharmaceutical and agricultural industries for improving the pharmacological properties of drug candidates and natural products by tuning their binding affinities, solubilities, and metabolic profiles.^28^ However, conventional synthetic alkylating methods have several disadvantages, including toxic reactants (such as alkyl halide) and a lack of robust regio-, chemo-, or stereoselectivities. By contrast, MTase-catalyzed methylations are highly regio- and stereoselective, making them promising biocatalysts for the late-stage diversification of complex molecules. There has been growing interest in exploiting MTases for synthetic applications in recent years.^29^ A variety of SAM analogs bearing different alkyl groups in place of the methyl substituent have been synthesized and used as cosubstrates in MTase-catalyzed reactions for various purposes.^30^ However, the transfer of fluorine-bearing alkyl groups is much more difficult due to the instability of the corresponding SAM analog.^31^

The introduction of fluorine into pharmaceuticals, agrochemicals, and other molecules of value is an ongoing and major focus of synthetic chemists because of the unique properties that fluorine atoms confer on molecules.^32^ In fact, fluorine is found in 20–30% of all pharmaceuticals and ∼30% of all agrochemicals.^33,34^ Due to the electronegativity of fluorine, C–F bonds can tune the strength of proximal bonds, resulting in a substantial impact on the physicochemical properties of an entire molecule.^35,36^ In addition, ^19^F, the only natural isotope of fluorine, provides a low-noise and highly sensitive tool to study drug–target interactions and other properties of molecules through nuclear magnetic resonance (NMR) spectroscopy.^37^ Due to the importance of fluorine-containing small molecules for therapeutics, there has been ongoing interest in developing strategies to incorporate fluorine atoms site-specifically into organic compounds, biochemical metabolites, and natural products.^38−48^ In this work, we describe the synthesis of *Te*-adenosyl-*L*-(fluoromethyl)homotellurocysteine (FMeTeSAM) and its application in the fluoromethylation of biological targets (Figure 1B).

## Design and Synthesis of FMeTeSAM

**Figure 2:**
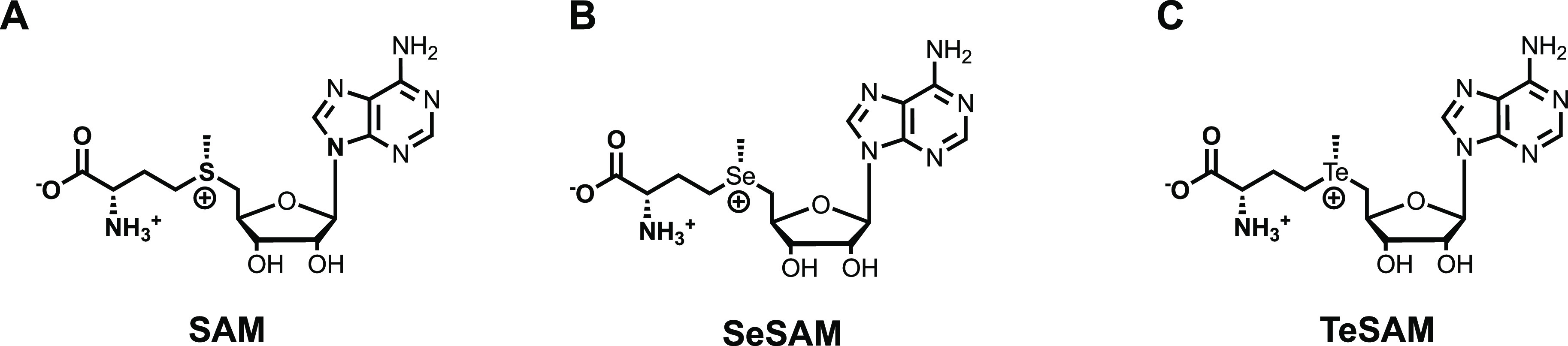
Chemical structures of (A) SAM, (B) SeSAM, and (C) TeSAM

In our studies of the enzyme cyclopropane fatty acid (CFA) synthase, we synthesized the *Se*- and *Te*-containing analogs of SAM (*Se*-adenosyl-*L*-selenomethionine and *Te*-adenosyl-*L*-telluromethionine (SeSAM and TeSAM, respectively)) (Figure 2) as probes of the enzyme’s reaction mechanism and showed that TeSAM exhibits marked stability over SAM and SeSAM.^49^

SAM exhibits a half-life on the order of days at pH 7.5–10.0 and 37 °C and degrades in three distinct ways (Figure S1). It undergoes racemization at the sulfur to afford the inactive (*R*, *S*) diastereomer. At a pH above 3, it undergoes an intramolecular cyclization to render methylthioadenosine (MTA) and homoserine lactone. At pH values above 7, it undergoes deprotonation at C5′, which results in the elimination of adenine and the production of *S*-ribosylmethionine.^49−54^ SeSAM does not undergo racemization and only undergoes deprotonation at C5′ at pH values above 12.0. However, it degrades to homoserine lactone and methylselenoadenosine about 10-fold faster than SAM. By contrast, TeSAM does not undergo any significant degradation at 37 °C in the pH range of 2–12. Moreover, we found that *L*-telluromethionine appears to be as good as *L*-methionine in SAM synthetase reactions under saturating conditions, suggesting the possibility of generating FMeTeSAM enzymatically from *L*-(fluoromethyl)homotellurocysteine.^49^ Importantly, both SeSAM and TeSAM were good cosubstrates in methylation reactions catalyzed by CFA synthase and catechol *O*-methyltransferase (COMT).^55^ Given the stability of TeSAM and the ease by which it can be synthesized enzymatically, we posited that it might be possible to generate and isolate its fluoromethyl analog. Recently, Seebeck and colleagues reported the *in situ* generation of fluoromethyl-SAM—using fluoromethyl iodide, SAH, and halide methyltransferase—and its application in transferring fluoromethyl groups to a variety of nucleophiles. Although this strategy was clever, the fluoromethyl-SAM was highly unstable and could not be isolated for kinetic studies.^31^

The synthesis of FMeTeSAM was completed in six steps starting from *L*-homoserine, with the final step involving an enzymatic transformation (Scheme S1). NMR spectroscopy and high-resolution mass spectrometry (HRMS) verified the final product’s authenticity. In particular, the mass spectrum of FMeTeSAM shows the characteristic isotopic distribution associated with tellurium, with the major isotopes being ^130^Te (34.08%), ^128^Te (31.74%), ^126^Te (18.84%), ^125^Te (7.07%), ^124^Te (4.74%), and ^122^Te (2.55%). ^19^F-NMR shows a resonance split into a triplet by the two protons on the fluoromethyl group. Moreover, the resonance is highly upfield (−229 ppm), due to the attachment of the fluoromethyl group to the positively charged tellurium atom (see Supporting Information).

## Fluoromethyl Transfer to Oxygen Nucleophiles

Mammalian COMT has served as a paradigm for enzymatic SAM-dependent methyl transfer.^21,56−62^ Its normal function is to methylate hydroxyl groups on catechols, catecholamines, and other small molecules to prepare biologically active and/or potentially toxic hydroxylated metabolites for elimination. It is especially important in the metabolism of catecholamine neurotransmitters and catechol estrogens.^63^ Because of its historic role in early studies of the mechanism of SAM-dependent MT, COMT was chosen as an initial model to address the transfer of the fluoromethyl group from FMeTeSAM to dihydroxybenzoic acid (DHBA), one of its known substrates. Using SAM as a methylating agent, COMT methylates DHBA with *k*_cat_ and *k*_cat_/*K*_m_ values of 1.34 ± 0.08 min^–1^ and 0.65 ± 0.17 μM^–1^ min^–1^, respectively (Table 1). In the presence of TeSAM, *k*_cat_ is reduced only by a factor of 2, although *k*_cat_/*K*_m_ is reduced by a factor of almost 34. Excitingly, COMT also uses FMeTeSAM to fluoromethylate DHBA, exhibiting *k*_cat_ and *k*_cat_/*K*_m_ values of 0.281 ± 0.011 min^–1^ and 0.00819 ± 0.00113 μM^–1^ min^–1^, respectively. The *k*_cat_ value for the reaction is reduced only by a factor of ∼5 from that with SAM, indicating that FMeTeSAM is a robust fluoromethylating agent under saturating conditions. As expected, the substantial increase in the size of tellurium (covalent radius, 135 pm) over sulfur (covalent radius, 103 pm) drives the *K*_m_ value for TeSAM upward, and the addition of the fluorine atom increases it further, which impacts the *k*_cat_/*K*_m_ values substantially.^64^ Nevertheless, these *K*_m_ values are still lower than or on the order of the *in vivo* concentrations of SAM in most organisms.^65−67^

**Table 1:**
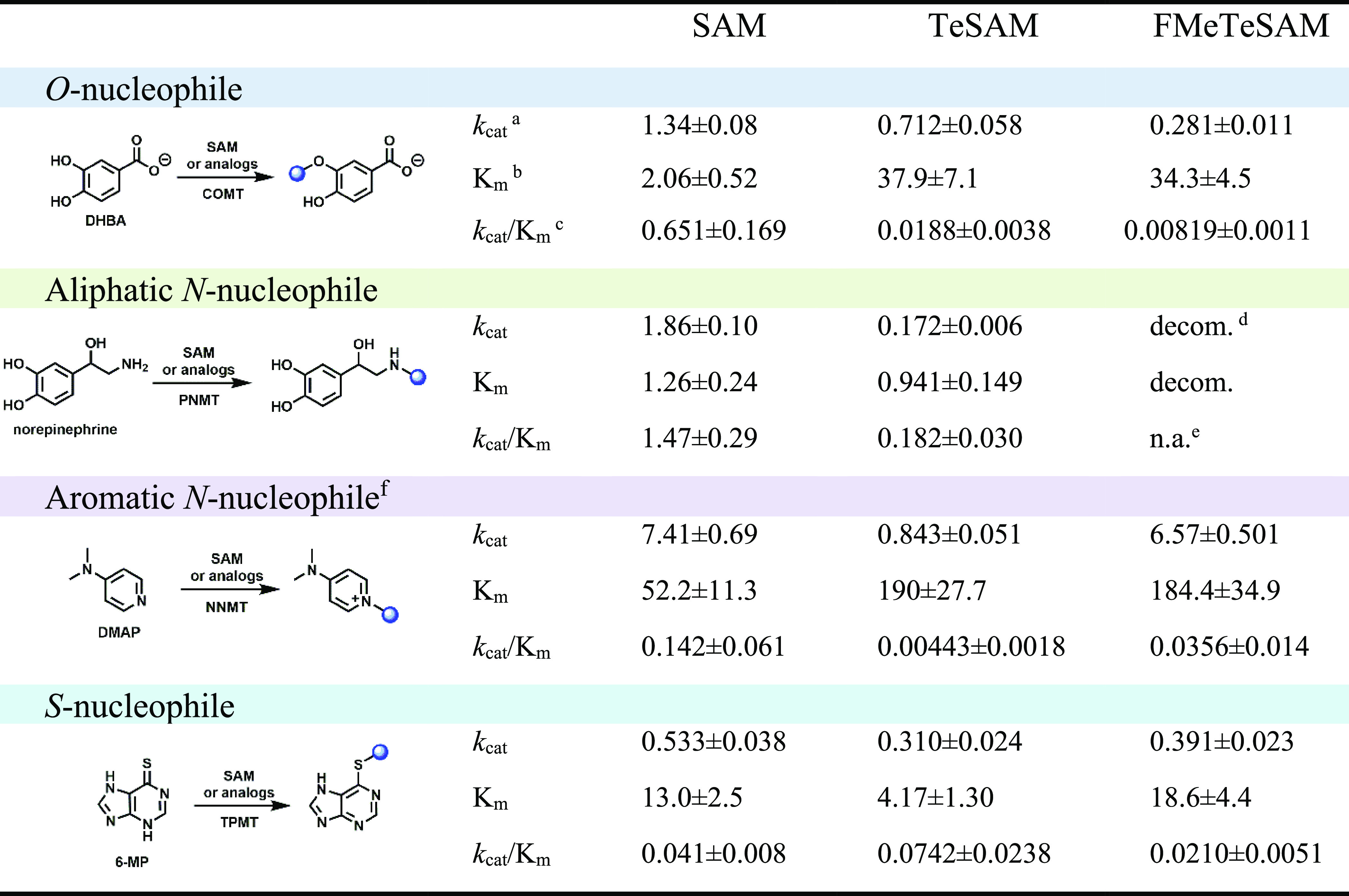
Nucleophile Scopes and Key Kinetic Parameters

a Unit of *k*_cat_ is min^–1^.

b Unit of *K*_m_ is μM.

c Unit of *k*_cat_/*K*_m_ is μM^–1^min^–1^.

d Monofluoromethylation on aliphatic amines resulted in carbinolamine decomposition followed by hydrolysis, presumably giving fluoride ion and formaldehyde.

e Not applicable.

f *k*_cat_ and *k*_cat_/*K*_m_ for NNMT is multiplied by 10^–3^.

## Fluoromethyl Transfer to Nitrogen Nucleophiles

We also assessed whether FMeTeSAM could be used to fluoromethylate nitrogen nucleophiles, among the most abundant targets of SAM-derived methyl groups. Indeed, nitrogen atoms on DNA and RNA bases, phospholipid head groups, lysine, histidine, and arginine side chains on proteins, and various small molecules are methylated by SAM-dependent MTases.^5,7,8,14,15,18,19,68^ Phenylethanolamine *N*-methyltransferase (PNMT), nicotinamide *N*-methyltransferase (NNMT), and TrmD were chosen as test cases. PNMT methylates norepinephrine, affording epinephrine, while NNMT methylates nicotinamide, yielding 1-methylnicotinamide.^69−76^ Norepinephrine and epinephrine serve as hormones and neurotransmitters, while 1-methylnicotinamide is produced predominantly in human fat and liver cells, where it has been reported to play a role in obesity and type 2 diabetes.^72,77,78^ Moreover, NNMT is upregulated in several cancers.^79−83^ Last, TrmD is involved in tRNA modification.^84−86^

**Figure 3:**
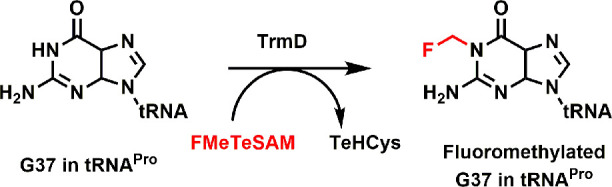
Methylation/fluoromethylation of tRNA guanine N37 by TrmD using SAM, TeSAM, and FMeTeSAM.

The kinetic parameters for PNMT using SAM, TeSAM, or FMeTeSAM as the methyl or fluoromethyl donor are displayed in Table 1. When SAM is used as the methyl donor, the enzyme exhibits a *k*_cat_ of 1.86 ± 0.1 min^–1^ and a *K*_m_ of 1.26 ± 0.24 μM. The *k*_cat_ when using TeSAM as the methyl donor is about 10-fold lower, although *K*_m_ remains relatively unchanged. Interestingly, no fluoromethyl-containing product is observed in the reaction using FMeTeSAM as the methyl donor. However, a careful analysis of the reaction by mass spectrometry revealed that *Te*-adenosyl-*L*-homotellurocysteine (TeHCys) is produced in a time-dependent manner, suggesting that methyl transfer does indeed take place (Figure S3). It is known that the attachment of fluoromethyl groups to aliphatic amines results in hydrolysis with the concomitant release of formaldehyde and fluoride.^87^ Given the instability of these fluoromethylated products, we also tested molecules containing nitrogen atoms in different electronic environments as substrates for *N*-fluoromethylation.^88^ Results of a kinetic analysis of NNMT-catalyzed methylation of 4-dimethylaminopyridine (DMAP) using SAM, TeSAM, or FMeTeSAM as the methyl donor are also displayed in Table 1. In this instance, FMeTeSAM is equivalent to or better than SAM as a methylating agent under saturating conditions. However, the *K*_m_ for FMeTeSAM is approximately 3.5 times higher than that of SAM, resulting in a *k*_cat_/*K*_m_ that is ∼4-fold lower than that when using SAM as the methyl donor. 4-DMAP was chosen as a substrate for NNMT because the native substrate, nicotinamide (NAM), did not yield detectable levels of the fluoromethylated product (fm-NAM), although the coproduct TeHCys was detected in our LC–MS analysis. The fm-NAM could have been degraded via hydrolysis generating formaldehyde and fluoride. It should be noted that the *k*_cat_ and *k*_cat_/*K*_m_ of SAM and SAM analogs with NNMT using 4-DMAP as a cosubstrate are approximately ∼1000-fold lower compared to other MTases used in this study. In addition, we studied *E. coli* TrmD, which methylates N1 of guanine37 (G37) in tRNA^pro^ (Figure 3), and found that the enzyme can indeed fluoromethylate G37 using FMeTeSAM (Figure S5). As expected, it also uses TeSAM and SAM to transfer methyl groups to G37 of tRNA^pro^. TrmD (15 μM) converts approximately 54% of the substrate (180 μM) to the fluoromethylated product in 2 min.

## Fluoromethyl Transfer to Sulfur Nucleophiles

Thiopurine methyltransferase (TPMT) was used to investigate fluoromethyl transfer to sulfur nucleophiles. TPMT is involved in the metabolism of thiopurine drugs such as 6-mercaptopurine, 6-thioguanine, and azathioprine, which are cytotoxic immunosuppressant compounds used to treat childhood acute lymphoblastic leukemia, inflammatory bowel disease, and rheumatological diseases.^89−91^ Their methylation by TPMT reduces their cytotoxic effects.^92,93^ Surprisingly, FMeTeSAM is as good a methylating agent under saturating conditions as SAM, and the *K*_m_ for FMeTeSAM is nearly the same as that for SAM. TeSAM supports about 58% of the activity exhibited with SAM under saturating conditions (Table 1).

## Fluoromethyl Transfer to Carbon Nucleophiles

Several *C*-methyltransferases were chosen to investigate fluoromethyl transfer to carbon nucleophiles. These *C*-MTases include SgvM, a phenyl pyruvic acid MTase;^94^ M. SssI, a cytosine C5 DNA MTase;^95,96^ and NovO, an MTase involved in the biosynthesis of the antibiotic novobiocin.^97−99^ Initially, a series of phenyl pyruvic acid analogs bearing various substituents on the phenyl ring were used to study SgvM (Figure S7); however, none of these compounds yielded detectable fluoromethylated products as judged by HRMS. Moreover, some of the compounds did not support methylation by TeSAM or SAM. A similar result was obtained with M. SssI, suggesting that these carbon centers may not be sufficiently nucleophilic to displace the fluoromethyl group from FMeTeSAM (Figure S8).

**Figure 4:**
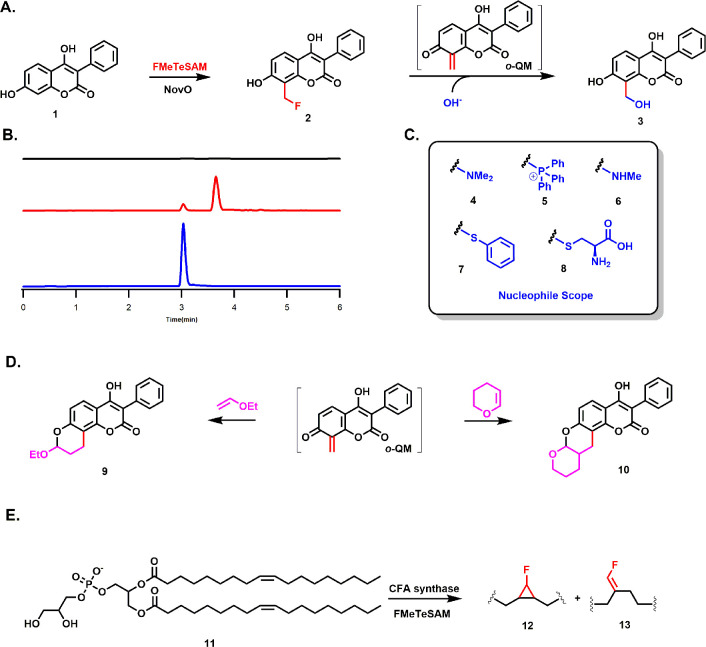
(A) NovO catalyzes *C*-fluoromethylation using FMeTeSAM on coumarin **1** to yield an *o*-QM intermediate, which is trapped by a water molecule. (B) EIC trace (black) of reaction conducted in the absence of NovO, showing no fluoromethylated product **2** (*m*/*z* 285); EIC trace (red) of reaction in the presence of NovO and FMeTeSAM, showing a fluoromethylated product **2** (*m*/*z* 285); EIC trace (blue) of hydroxymethylated product **3** (*m*/*z* 283) from reaction of NovO using FMeTeSAM. (C) Nucleophiles tested in the NovO reaction. Other nucleophiles (triethylamine, ethanolamine, potassium cyanide, sodium iodide) failed to give corresponding adducts. (D) Dienophiles tested with the *o*-QM intermediate. (E) Fluoromethylation of dOPG **11** catalyzed by CFA synthase using FMeTeSAM as the methyl donor. The reaction gave two products, including a fluorocyclopropane species **12** and a terminal fluoroalkene **13***via* rearrangement.

Studies with NovO and CFA synthase were more successful. NovO methylates C8 of the coumarin scaffold during the biosynthesis of novobiocin, an antibiotic that targets bacterial DNA gyrase. NovO has been reported to exhibit a broad substrate scope. Therefore, the commercially available compound **1** was used as our test substrate.^98^ In the absence of NovO, the fluoromethylated product **2** (*m*/*z* 285) is not observed (Figure 4B, black trace). By contrast, when NovO is added to the reaction, a product exhibiting *m*/*z* 285 is detected at a retention time of 3.8 min (Figure 4B, red trace). The exact mass of the fluoromethylated product **2** (observed *m*/*z* 287.0718, calculated *m*/*z* 287.0714) was verified by HRMS (Figure S9). Interestingly, a second peak (*m*/*z* 283) is observed at a retention time of 3 min (Figure 4B, blue trace), which corresponds to the hydroxymethylated product **3** (Figure 4A) as verified by HRMS (Figure S9).

The NovO reaction mechanism involves an active site Arg–His dyad that deprotonates the 7-OH group of the substrate, which activates the substrate for the rate-limiting methyl transfer (Figure S10).^99^ We believe that the active site-assisted deprotonation combined with the leaving group ability of fluorine in the fluoromethylated product **2** induces the formation of an *o*-quinone methide (*o*-QM) intermediate, which can be attacked by a water molecule to give the hydroxymethylated product **3**. *o*-QM has been implicated in the biosynthesis of several families of natural products as well as in the chemical synthesis of bioactive compounds as an important precursor.^100^ To provide additional evidence for the *o*-QM intermediate and to further derivatize this species, we tested other types of nucleophiles, as shown in Figure 4C. Reactions with thiol and phosphine nucleophiles proceed smoothly to the corresponding adducts, which were confirmed by HRMS (Figure S9, panels 1, 4, and 5). Amino nucleophiles, including methylamine and dimethylamine (Figure S9, panels 2 and 3, respectively), give only low amounts of adducts due to their lower nucleophilicities. The *o*-QM is known to react with dienophiles in a Diels–Alder fashion.^101,102^ Therefore, we assessed whether the *o*-QM species generated via fluoromethylation reacts with ethyl vinyl ether and tetrahydropyran, as shown in Figure 4D. The corresponding adducts were detected and verified by HRMS as shown in Figure S9 (panels 6 &7).

CFA synthase was employed to ascertain whether a less activated carbon nucleophile could be fluoromethylated using FMeTeSAM, as shown in Figure 4E. CFA synthase catalyzes the SAM-dependent cyclopropanation of isolated fatty acid olefinic bonds in membrane phospholipids, yielding SAH and a proton as secondary products.^103,104^ As shown in Figures S11–S13, *E. coli* CFA synthase can use FMeTeSAM to catalyze cyclopropanation on a 1,2-dioleoyl-*sn*-glycero-3-phospho-(1′-*rac*-glycerol) (dOPG) substrate **11**, yielding a fluorinated cyclopropane phospholipid product **12**, which was observed by HRMS and confirmed using MS/MS and ^19^F-NMR. Interestingly, two peaks with different retention times (4.60 and 5.00 min) but identical *m*/*z* values and mass spectra are observed by electrospray ionization in negative mode (ESI^–^) (Figure S11), both of which correspond to the addition of 1 CHF (32.0062 Da) moiety to dOPG (*m*/*z* 805.5429) substrate **11**. However, the peaks display distinct MS/MS spectra (Figure S12). The peak at 4.6 min contains a fragment ion from the loss of HF, observed both in ESI^–^ and in ESI positive mode (ESI^+^), while the peak at 5.00 min does not. This behavior implies that the two peaks correspond to different compounds. Based on this data, we identified these two compounds as **12** and **13** (Figure 4E). Compound **12** is the expected fluorinated cyclopropane product, while compound **13**, the identity of which was confirmed by LC–MS/MS and ^19^F-NMR (Figures S11–S13), presumably arises from a 1,2-hydride shift after methyl transfer. A 1,2-hydride shift after methyl transfer to an isolated alkene to stabilize the secondary carbocation intermediate has been observed previously in the biosynthesis of tuberculostearic acid.^105^ Taken together, our studies demonstrate the versatility of the FMeTeSAM as a methyl donor by showing that it can be used to fluoromethylate unactivated carbon nucleophiles.

## Late-Stage Fluoromethylation on Natural Products

**Figure 5:**
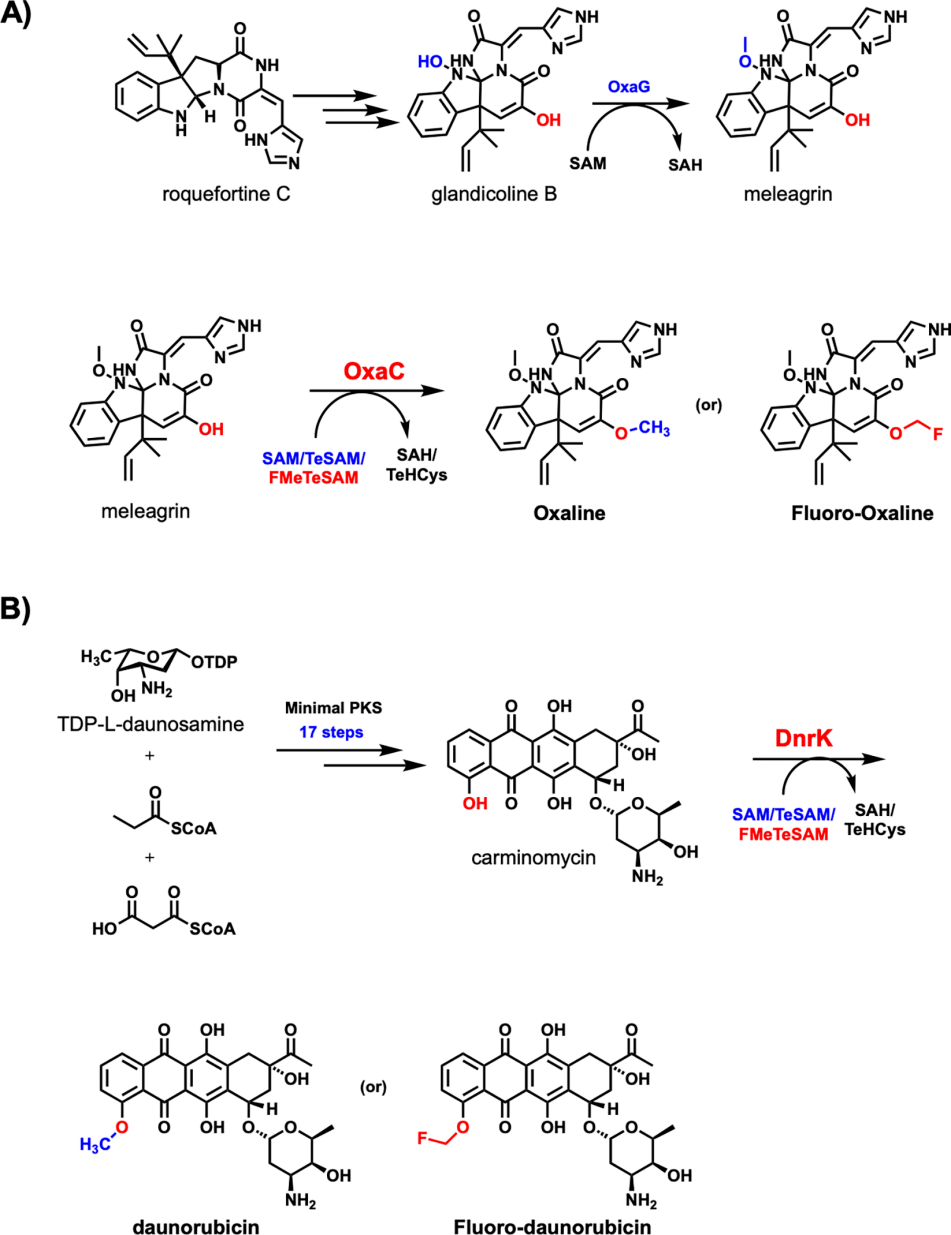
(A) OxaC and (B) DnrK are applied for the late-stage fluoromethylation.

The complicated chemical structures of natural products often render them difficult to modify in a selective way. However, late-stage derivatization strategies may provide an efficient means for the diversification of natural products. Therefore, the enzymes OxaC and DnrK were used to demonstrate that they can use FMeTeSAM to transfer fluoromethyl groups onto more complex scaffolds. OxaC catalyzes the penultimate step in the biosynthesis of oxaline, while DnrK catalyzes the penultimate step in the biosynthesis of daunorubicin (Figure 5A,B). Oxaline is a fungal alkaloid that exhibits anticancer activity *in vitro*, arresting the cell cycle in M phase by inhibition of tubulin polymerization.^106^ By contrast, daunorubicin is used as a chemotherapeutic agent to treat various types of leukemias and Kaposi’s sarcoma.^107^

End point assay analysis shows that both oxaline and daunorubicin are fluoromethylated by OxaC (Figure 5A) and DnrK (Figure 5B), respectively, with detection and verification by UPLC–MS and HRMS (Figures S12 and S13). Using FMeTeSAM as fluoromethylating reagent, 5 μM OxaC converts approximately 90% of an 0.15 mM substrate to product in 1 min, and 50 μM DnrK converts ∼99% of an 0.2 mM substrate to product in 1 min. This work shows that FMeTeSAM can serve as a regioselective fluoromethylating reagent on complex natural products, highlighting its potential use in late-stage derivatization processes.

## Summary and Conclusions

In this work, we reported the design and synthesis of FMeTeSAM, the only known stable and isolable SAM analog that bears a fluoromethyl group. Indeed, we showed that several SAM-dependent methyltransferases can use FMeTeSAM to transfer fluoromethyl groups to biologically relevant nucleophiles, such as *O*-, *N*-, *S*-, and some nucleophilic carbon atoms. The kinetic properties of the enzymes when using FMeTeSAM were robust; in most instances where kinetic parameters were determined, the *k*_cat_ of the reaction was within a factor of 2 of that in the presence of SAM, although the *K*_m_ value, understandably, due to the increased size of the tellurium atom, increased by up to a factor of 10, driving the second-order rate constants for some of the reactions (*k*_cat_·*K*_m_^–1^) downward. Although the fluoromethylation of *O*-, *S*-, and *C*- atoms gave stable and isolable products, the fluoromethylation of *N*- atoms often gave unstable adducts that presumably were hydrolyzed to formaldehyde and fluoride ion. However, some *N*- atoms as constituents of aromatic heterocycles did indeed support the formation of stable *N*-fluoromethylated products. OxaC and DnrK, two MTases that are involved in the biosynthesis of oxaline and daunorubicin, were active in the fluoromethylation of natural product scaffolds, demonstrating the potential application for late-stage fluoromethylation on more complicated molecules. FMeTeSAM could potentially be utilized in biocatalytic strategies to derivatize natural products or synthetic scaffolds whose structures are similar to those of the parent natural product, thereby generating novel fluorinated leads in a regioselective manner.

## Supporting Information Available

Kinetic plots, high-resolution liquid chromatography–mass spectrometry (LC–MS) spectra and chromatograms, reaction schemes, HPLC chromatograms (Figures S1–S17, Schemes S1–S5, Tables S1–S2), DNA and protein sequences, experimental procedures for cloning, protein expression and purification, and kinetic and enzyme assays, LC–MS quantification conditions, chemical synthetic procedures, and NMR spectra (PDF)

## Abbreviations

COMT: catechol *O*-methyltransferase
DHBA: dihydroxybenzoic acid
DMAP: 4-dimethylaminopyridine
FMeTeSAM: *Te*-adenosyl-*L*-(fluoromethyl)homotellurocysteine
HRMS: high-resolution mass spectrometry
LC–MS: liquid chromatography–mass spectrometry
MS: mass spectrometry
MTase: methyltransferase
MTA: methylthioadenosine
NMR: nuclear magnetic resonance
NNMT: nicotinamide *N*-methyltransferase
PNMT: phenylethanolamine *N*-methyltransferase
SAH: *S*-adenosylhomocysteine
SAM: *S*-adenosylmethionine
SeSAM: *Se*-adenosylselenomethionine
TeHCys: *Te*-adenosyl-*L*-homotellurocysteine
TPMT: thiopurine methyltransferase
TeSAM: *Te*-adenosyltelluromethionine
