# Supplementary material for: Enzymatic Fluoromethylation Enabled by the S-Adenosylmethionine Analog Te-Adenosyl-L-(fluoromethyl)homotellurocysteine
Source: ACS Cent Sci. 2023 May 8;9(5):905–14. doi: 10.1021/acscentsci.2c01385 (PMC10214534; doi:10.1021/acscentsci.2c01385)
Supplement: Supplementary file 1 — oc2c01385_si_001.pdf [file oc2c01385_si_001.pdf]

Supporting Information for:

Enzymatic Fluoromethylation Enabled by the *S*-adenosylmethionine analog *Te*-Adenosyl-L-(fluoromethyl)homotellurocysteine

Syam Sundar Neti,[1,3] Bo Wang[1,3], David F. Iwig,[1,3] Elizabeth Onderko,[1] and Squire J. Booker[1,2,3]**

Departments of [1]Chemistry and [2]Biochemistry and Molecular Biology, and the [3]Howard Hughes Medical Institute, The Pennsylvania State University, University Park, Pennsylvania 16802, USA.

*To whom correspondence should be addressed: Squire J. Booker (squire@psu.edu) or Bo Wang (bzw10@psu.edu).

TABLE of CONTENTS

Page S2. Table of contents

Page S3. Figure S1. Three degradation pathways of SAM

Page S4. Figure S2. Kinetic Parameters of COMT

Page S5. Figure S3. Kinetic Parameters of PNMT

Page S6. Figure S4. Kinetic Parameters of NNMT

Page S7. Figure S5. tRNA methylase TrmD

Page S8. Figure S6. Kinetic Parameters of TPMT

Page S9. Figure S7. Carbon methylase, SgvM

Page S10. Figure S8. DNA methyl transferase (M. SssI)

Pages S11-S12. Figure S9. Carbon methylase, NovO

Pages S13. Figure S10. Formation of *o*-QM by NovO

Pages S14-S15. Figure S11. CFA synthase reaction with FMeTeSAM

Page S16. Figure S12. MSMS spectra CFAS reaction with FMeTeSAM

Page S17. Figure S13. 19F NMR of lipid products from CFAS reaction with FMeTeSAM

Page S18. Figure S14. Natural product methyltransferase, DnrK reaction with FMeTeSAM

Page S19. Figure S15. Natural product methyltransferase, OxaC reaction with FMeTeSAM

Pages S20-S53. Experimental Procedures, synthesis of FMeTeSAM and product standards

Page S54. References

Pages S55-S79. NMR spectra

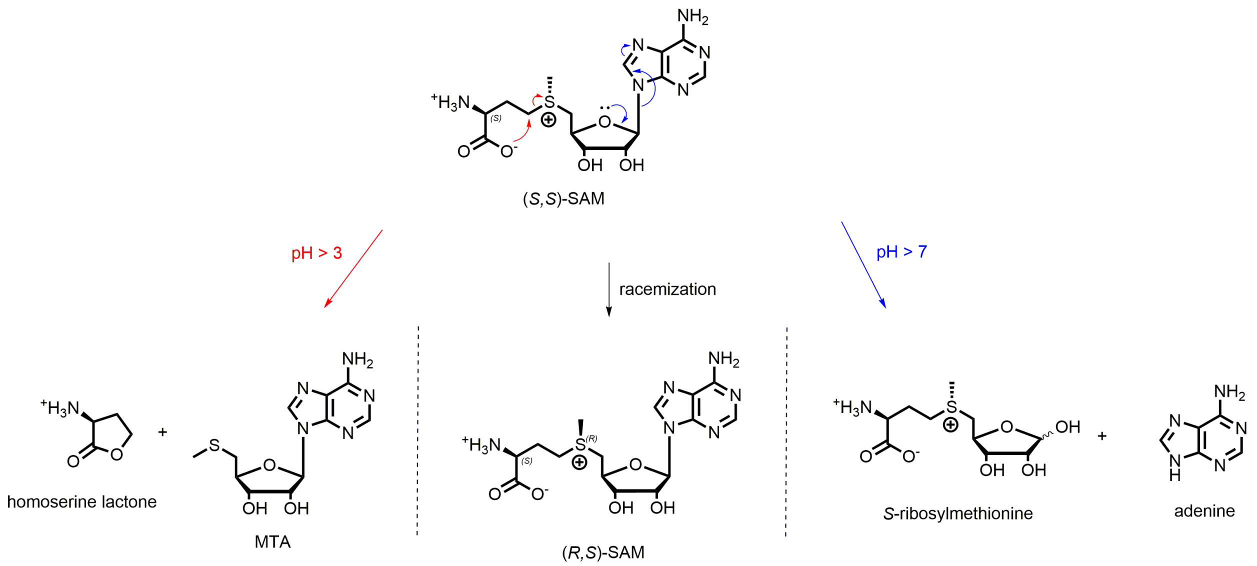

Figure S1. Three degradation pathways of SAM.

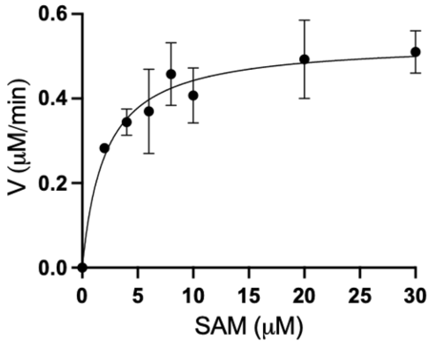

k_{cat} = 1.34 ± 0.08 min^{-1}
K_m = 2.06 ± 0.52 μM
k_{cat}/K_m = 0.651 ± 0.169 ($μM^{-1}$ min^{-1})

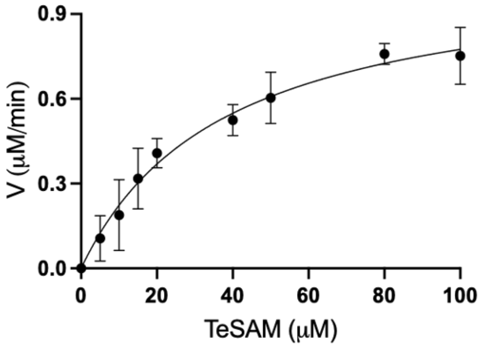

k_{cat} = 0.712 ± 0.058 min^{-1}
K_m = 37.9 ± 7.1 μM
k_{cat}/K_m = 0.0188 ± 0.0038 ($μM^{-1}$ min^{-1})

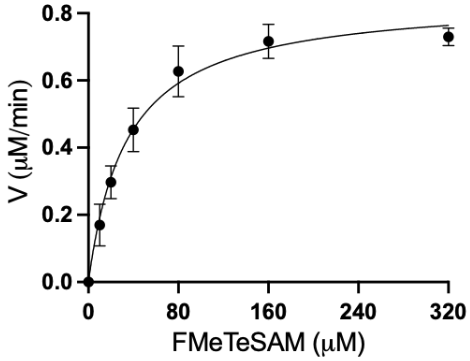

k_{cat} = 0.281 ± 0.011 min^{-1}
K_m = 34.3 ± 4.5 μM
k_{cat}/K_m = 0.00819 ± 0.00113 ($μM^{-1}$ min^{-1})

Figure S2. Steady-state kinetic analysis of COMT with SAM, TeSAM, and FMeTeSAM

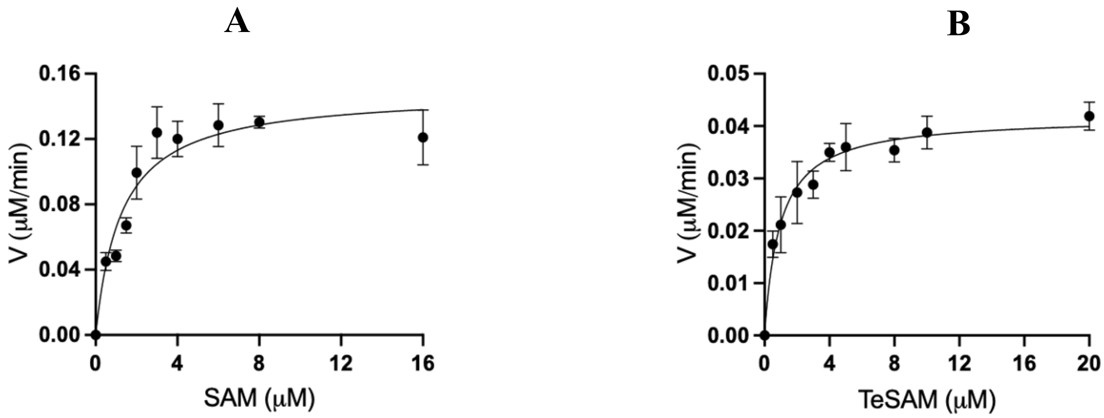

k_{cat} = 1.86 ± 0.1 min^{-1}
K_m = 1.26 ± 0.24 μM
k_{cat}/K_m = 1.47 ± 0.29 (μM^{-1} min^{-1})

k_{cat} = 0.172 ± 0.006 min^{-1}
K_m = 0.941 ± 0.149 μM
k_{cat}/K_m = 0.182 ± 0.030 (μM^{-1} min^{-1})

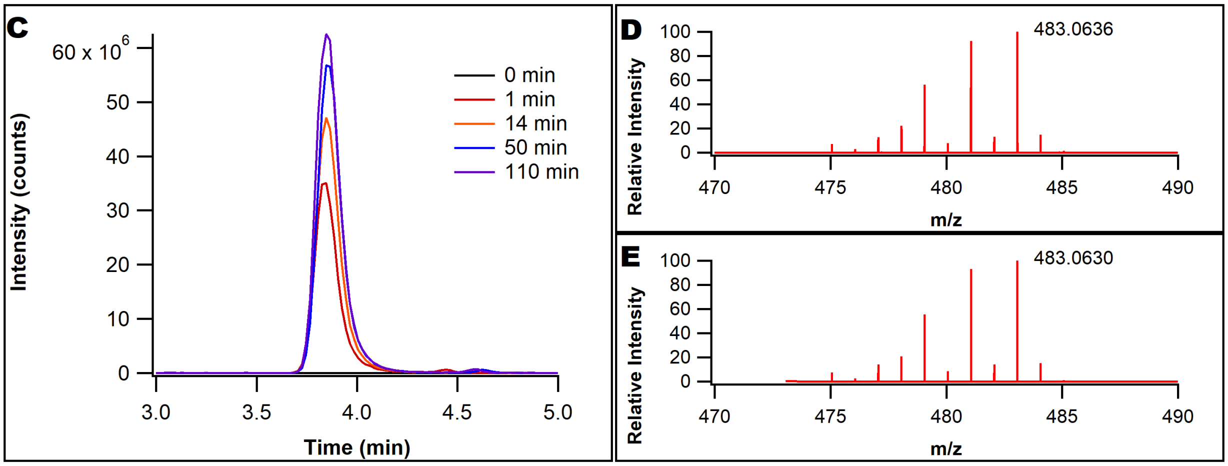

Figure S3. Steady-state kinetic analysis of PNMT with A) SAM, and B) TeSAM; C) High resolution-extracted ion chromatograms (HR-EIC) for TeHCys (*m/z* 483.0630) formed from the PNMT reaction using FMeTeSAM as the methyl donor. D) Observed mass spectrum of TeHCys displaying expected Te isotopic pattern, ^{130}Te (100%), ^{128}Te (93.1%), ^{126}Te (55.3%), ^{125}Te (20.7%), ^{124}Te (13.9%), ^{122}Te (7.48%), and ^{123}Te (2.61%). E) Simulated mass spectrum of TeHCys, $C_{14}H_{21}N_6O_5Te^+$ (Thermo Scientific Xcalibur 4.2.47).

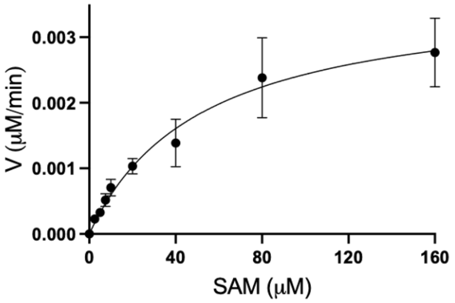

k_{cat} = 7.41 (x10^{-3}) ± 0.69 (x10^{-3}) min^{-1}
K_m = 52.2 ± 11.3 μM
k_{cat}/K_m = 0.142 (x10^{-3}) ± 0.061 (x10^{-3}) μM^{-1} min^{-1}

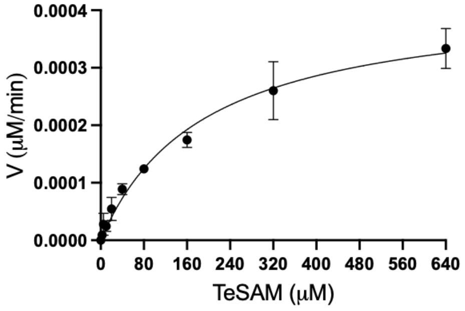

k_{cat} = 0.843 (x10^{-3}) ± 0.051 (x10^{-3}) min^{-1}
K_m = 190 ± 27.7 μM
k_{cat}/K_m = 0.00443 (x10^{-3}) ± 0.0018 (x10^{-3}) μM^{-1} min^{-1}

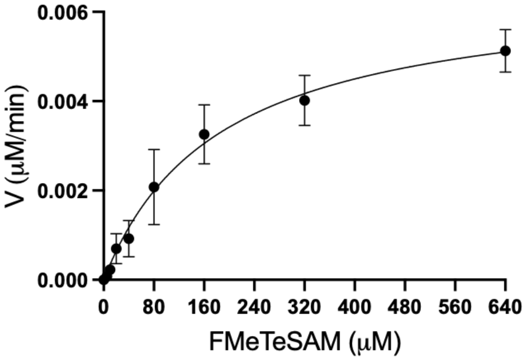

k_{cat} = 6.57 (x10^{-3}) ± 0.501 (x10^{-3}) min^{-1}
K_m = 184.4 ± 34.9 μM
k_{cat}/K_m = 0.0356 (x10^{-3}) ± 0.014 (x10^{-3}) μM^{-1} min^{-1}

Figure S4. Steady-state kinetic analysis of NNMT with SAM, TeSAM and FMeTeSAM

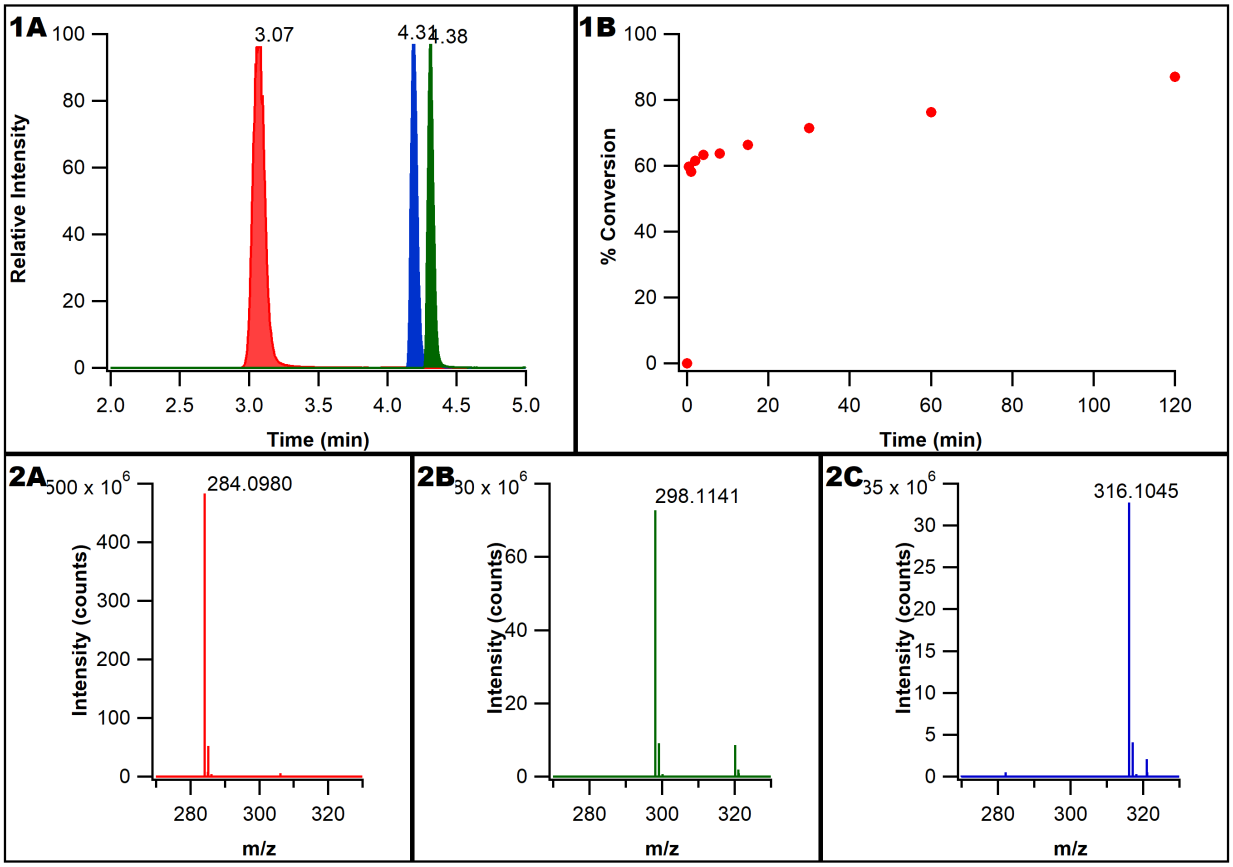

Figure S5. tRNA G37 methylation/fluoromethylation by TrmD using SAM or FMeTeSAM as the methyl donor. 1A) Normalized merged HR-EIC for guanosine (red, *m/z* 284.0980), m[1]-methylguanosine (green, *m/z* 298.1141), and m[1]-fluoromethylguanosine (blue, *m/z* 316.1045). 1B) Formation of m[1]-fluoromethylguanosine when FMeTeSAM was used as the methyl donor. Assays were performed as described in the text. % Conversion was calculated as the area response of the product divided by the total response of substrate and product. 2A-2C) Averaged high resolution mass spectra of guanosine (2A, red, 284.0990 calculated), m[1]-methylguanosine (2B, green, 298.1146 calculated), and m[1]-fluoromethylguanosine (2C, blue, 316.1052 calculated).

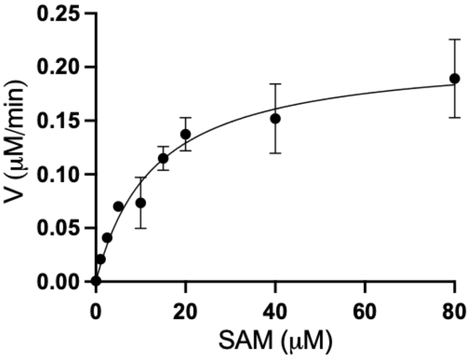

k_{cat} = 0.533 ± 0.037 min^{-1}
K_m = 13.0 ± 2.5 μM
k_{cat}/K_m = 0.041 ± 0.0083 (μM^{-1} min^{-1})

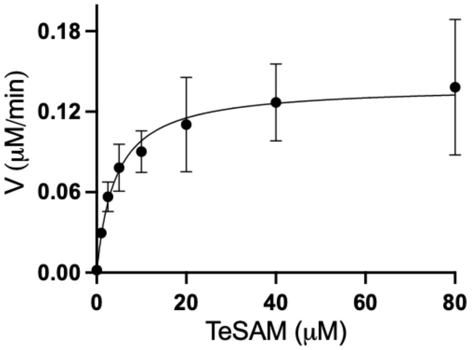

k_{cat} = 0.310 ± 0.024 min^{-1}
K_m = 4.17 ± 1.30 μM
k_{cat}/K_m = 0.0742 ± 0.0238 (μM^{-1} min^{-1})

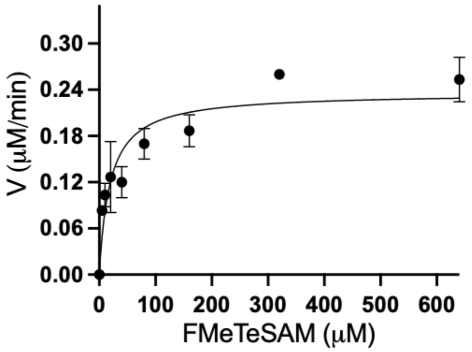

k_{cat} = 0.391 ± 0.023 min^{-1}
K_m = 18.6 ± 4.4 μM
k_{cat}/K_m = 0.0210 ± 0.0051 (μM^{-1} min^{-1})

Figure S6. Steady-state kinetic analysis of TPMT with SAM, TeSAM, FMeTeSAM

A.

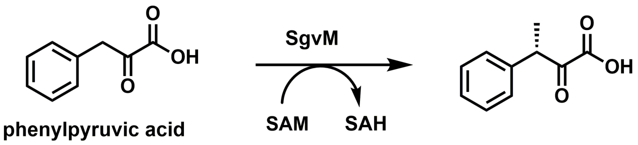

B.

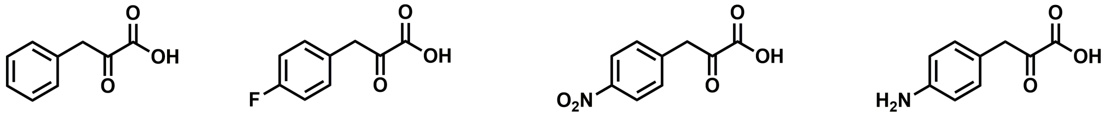

	phenylpyruvic acid	4-fluorophenylpyruvic acid	4-nitrophenylpyruvic acid	4-aminophenylpyruvic acid
SAM	+	+	-	-
TeSAM	-	+	-	-
FMeTeSAM	-	-	-	-

Figure S7. A) SgvM catalyzes C-methylation on phenylpyruvic acid to yield (S)-3-methylphenylpyruvic acid; B) Substrate scope tested for the C-methylation of SgvM. Positive sign (+) indicates methylated or fluoromethylated product was detected by HRMS. Minus sign (–) indicates no methylated or fluoromethylated product was detected by HRMS.

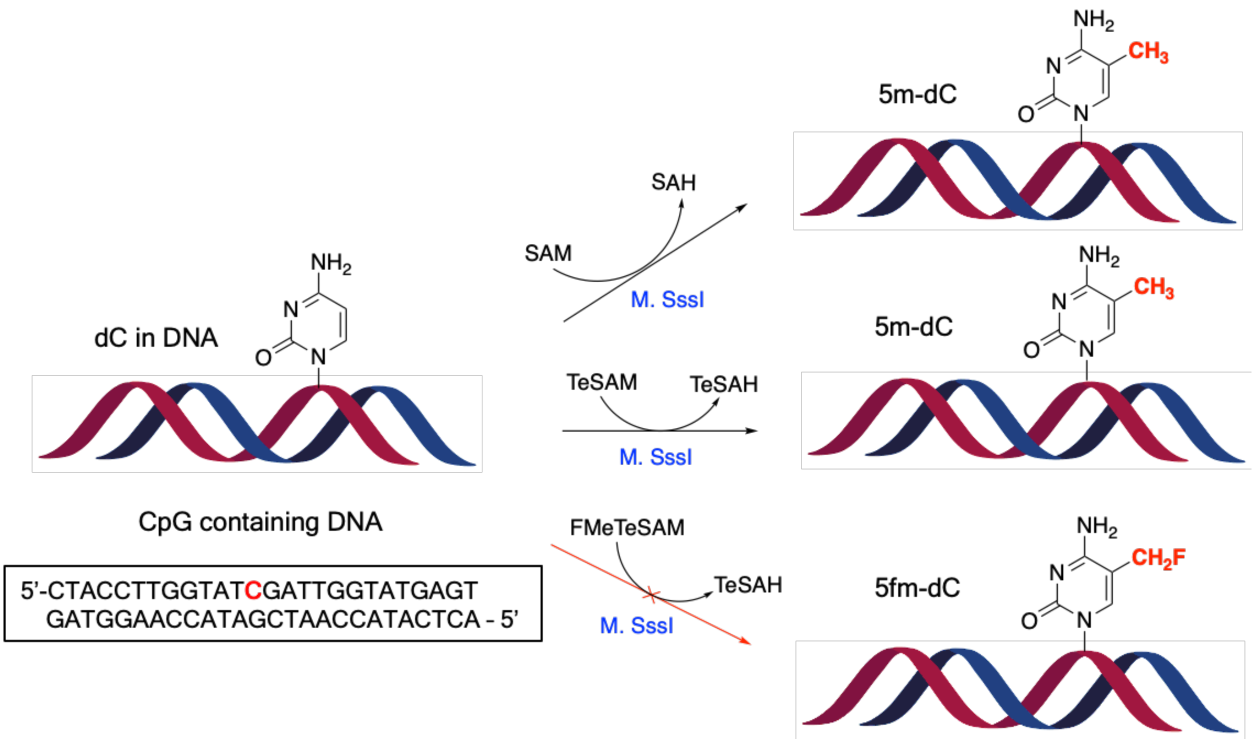

Figure S8. M. SssI catalyzed DNA-methylation.

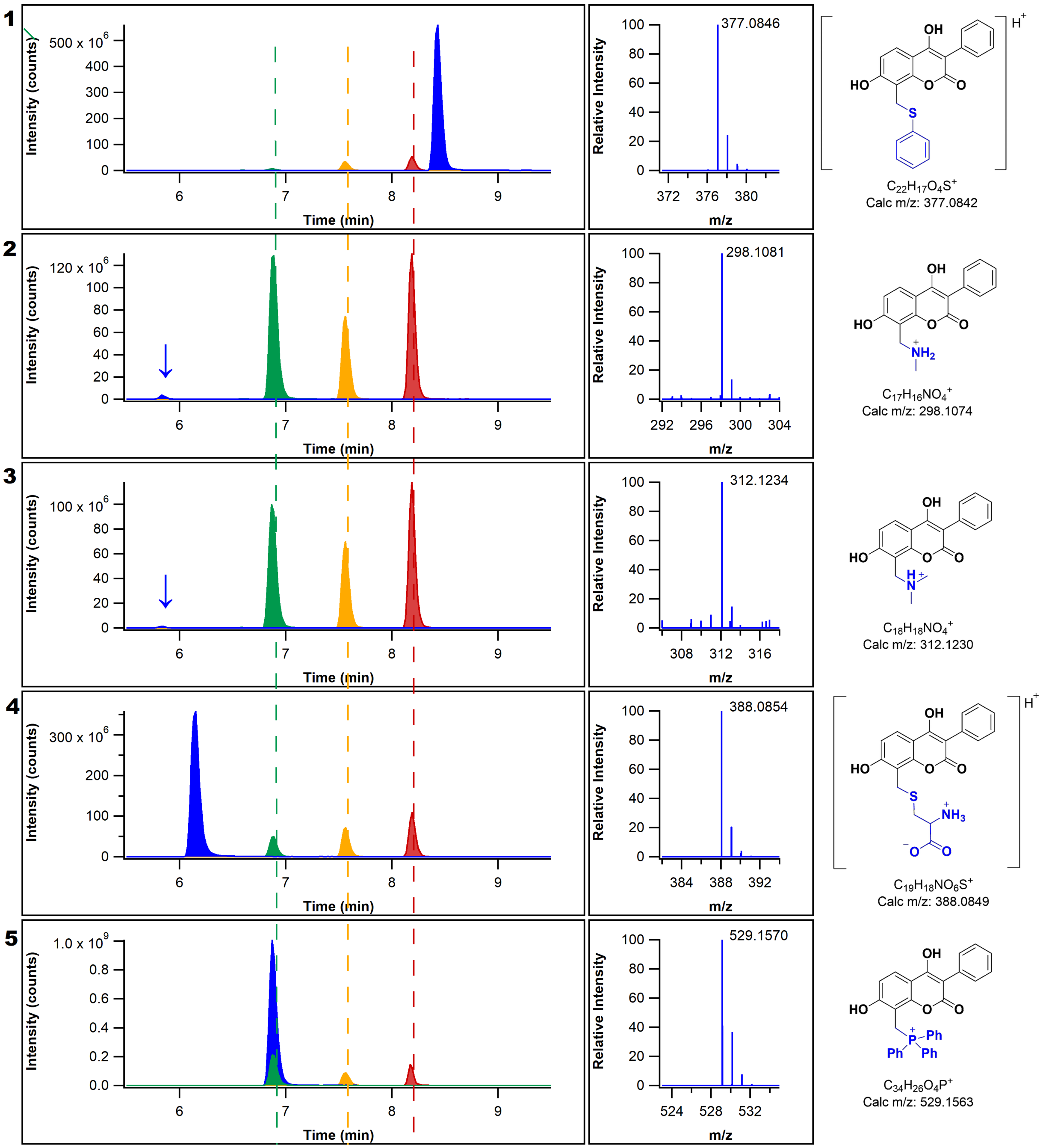

1
500 x 10^6
Intensity (counts)
Time (min)
377.0846
Relative Intensity
m/z
C22H17O4S+
Calc m/z: 377.0842
2
120 x 10^6
298.1081
C17H16NO4+
Calc m/z: 298.1074
3
100 x 10^6
312.1234
C18H18NO4+
Calc m/z: 312.1230
4
300 x 10^6
388.0854
C19H18NO6S+
Calc m/z: 388.0849
5
1.0 x 10^9
529.1570
C34H26O4P+
Calc m/z: 529.1563

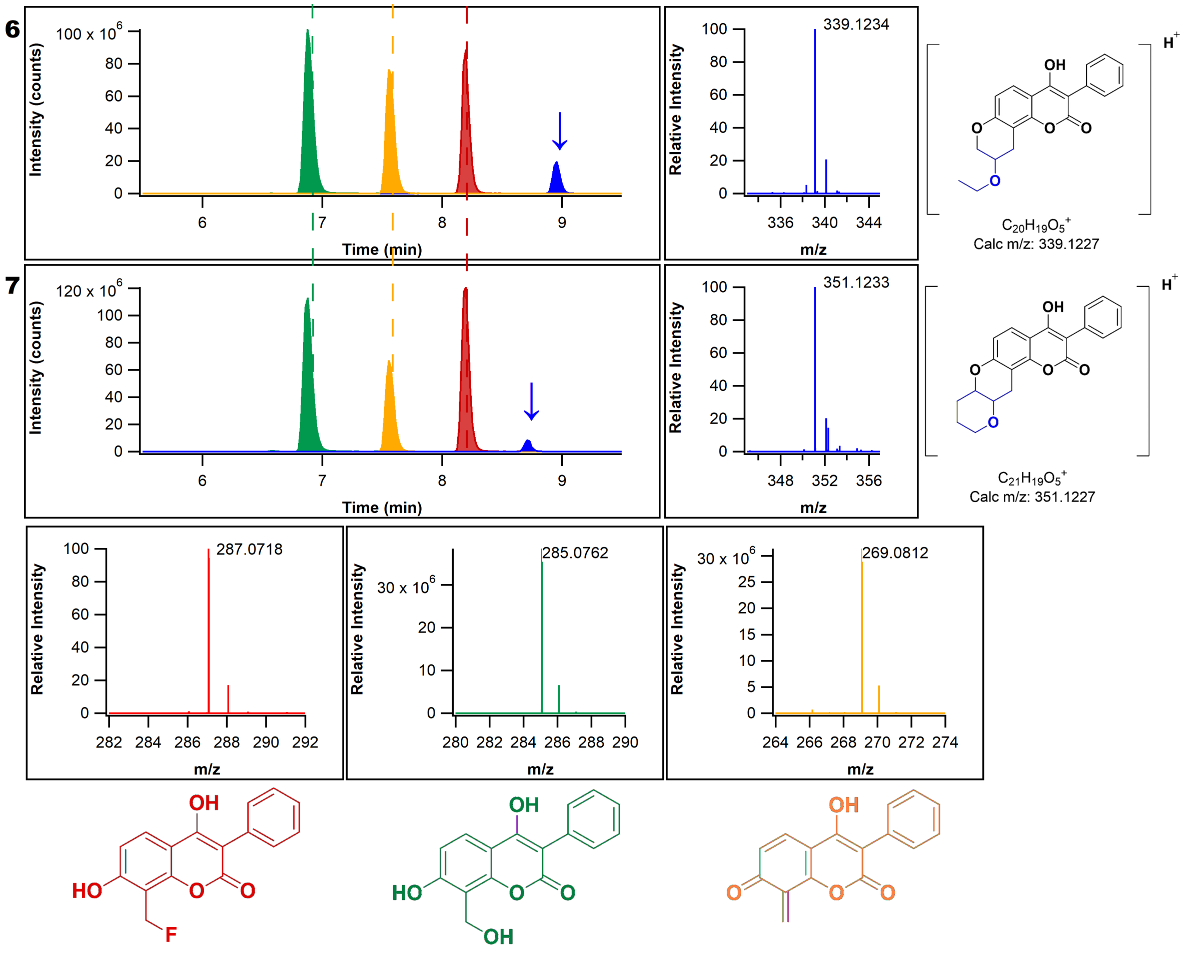

Figure S9. Reactions catalyzed by NovO using FMeTeSAM in the presence of nucleophiles or cycloaddition reagents. **Left Panel:** Merged HR-EICs of fluoromethylated product (red), hydroxylated byproduct (green), dehydration product (orange), and nucleophilic or cycloaddition products (blue traces and arrows); **Center Panel**: High resolution mass spectra of nucleophilic or cycloaddition adducts; **Right Panel:** Structures and calculated *m/z* (ESI^+) of nucleophilic (1: thiophenol, 2: methylamine, 3: dimethylamine, 4: cysteine, and 5: triphenylphosphine) or cycloaddition (6: ethyl vinyl ether and 7: 3,4-dihydro-2*H*-pyran) products; **Bottom Panel:** High resolution mass spectra of fluoromethylated product (red, observed *m/z* 287.0718, calculated *m/z* 287.0714), hydroxylated byproduct (green, observed *m/z* 285.0762, calculated *m/z* 285.0758), and dehydration product (orange, observed *m/z* 269.0812, calculated *m/z* 269.0808).

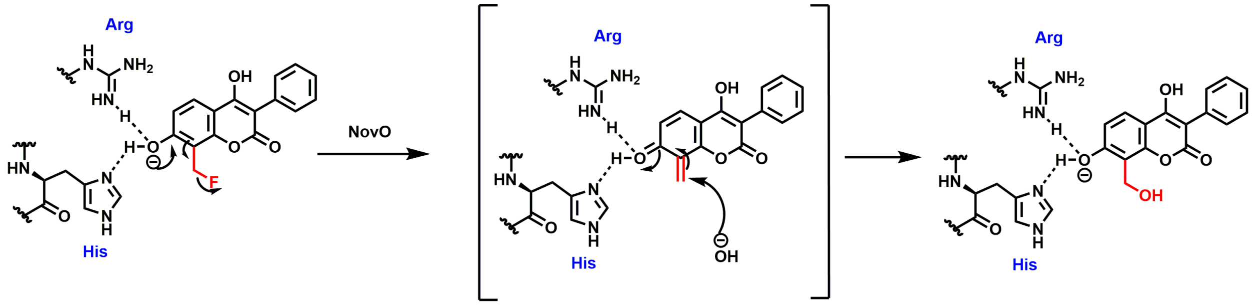

Figure S10. Formation of *o*-QM assisted by NovO.

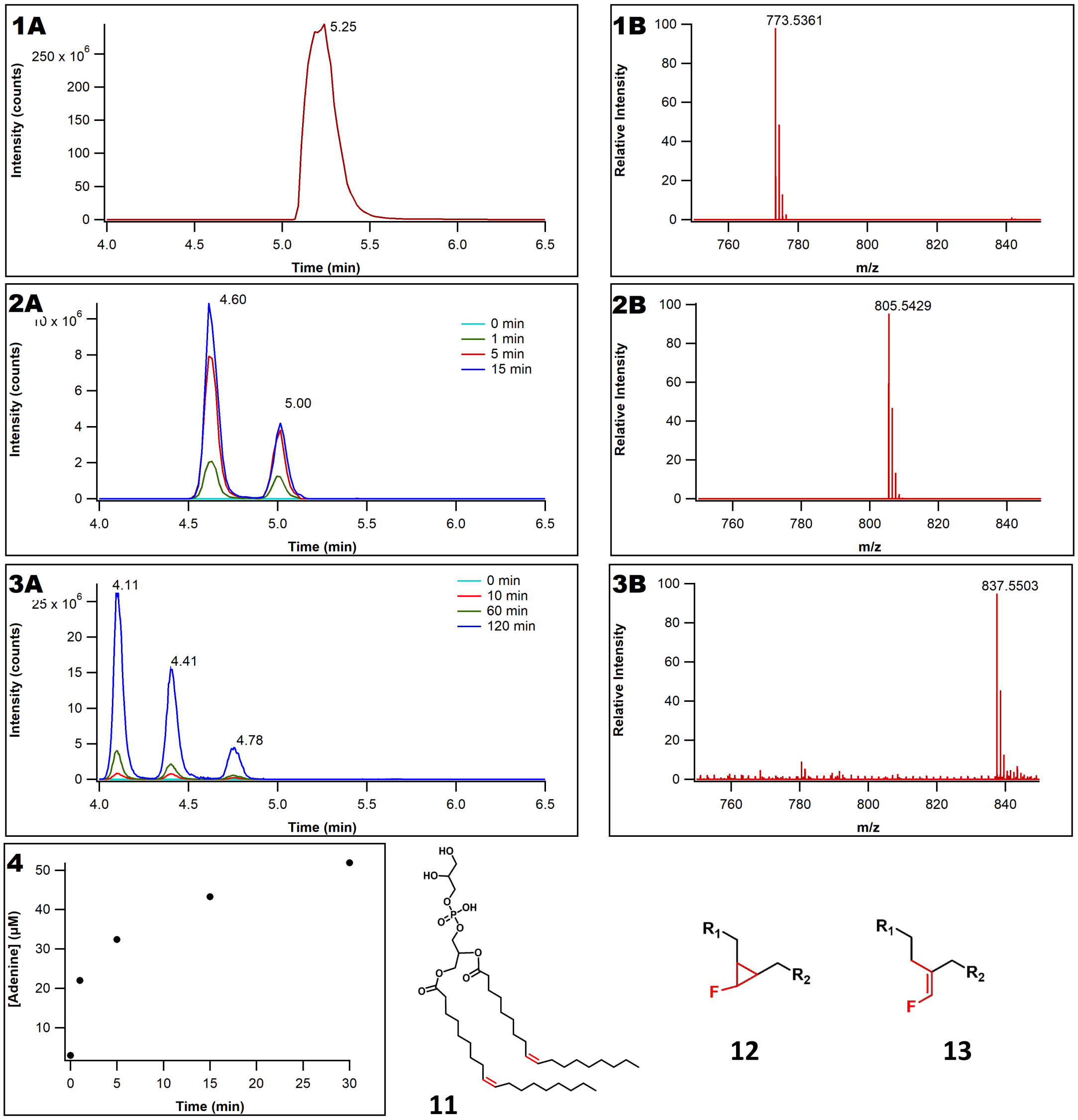

Figure S11. Reaction catalyzed by CFA synthase using FMeTeSAM as the methyl donor. Reactions were performed at 37 °C as described previously[4] and contained 7 µM CFAS, 1 µM SAH nucleosidase, 50 mM HEPES, pH 7.5, 5 mM dOPG, 5 mM $NaHCO_3$, and 500 µM FMeTeSAM. **1A-1B**: HR-EIC (ESI-, *m/z* 773.5361) (**1A**) and high-resolution mass spectrum (**1B**) of phospholipid methyl acceptor, dOPG (**11**). **2A.** HR-EIC for mono-fluorocyclopropane product (ESI-, *m/z* 805.5429) after 1 minute (green trace), 5 minutes (red trace), and 15 minutes (blue trace) reaction catalyzed by CFAS. The peak at 4.60 minutes corresponds to one CHF addition yielding the fluorocyclopropane (**12**); the peak at 5.00 corresponds to the fluorine rearrangement product (**13**). **2B**. HR mass spectrum of the mono-fluorocyclopropane products displaying a shift of 32.0068 from the substrate consistent with HCF (calculated 32.0062). **3A.** HR-EIC for di-fluorocyclopropane product (ESI-, *m/z* 837.5503) after 10 minute (red trace), 60 minutes (green trace), and 120 minutes (blue trace) reaction catalyzed by CFAS. The peak at 4.11 min corresponds to two CHF additions yielding the di-fluorocyclopropane product (**12**), the peak at 4.41 min corresponds to one chain containing the fluorocyclopropane (**12**) and one chain containing

the fluorine rearrangement product (**13**), and the peak at 4.78 min corresponds with the product containing two rearrangement products (**13**). **3B**. HR mass spectrum of the di-fluorocyclopropane products displaying a shift of 64.0142 from the substrate consistent with two equivalents of HCF (calculated 64.0124). **4**. Time dependent formation of adenine from the reaction catalyzed by CFAS using FMeTeSAM as the methyl donor when SAHN was included in the reaction mixture.

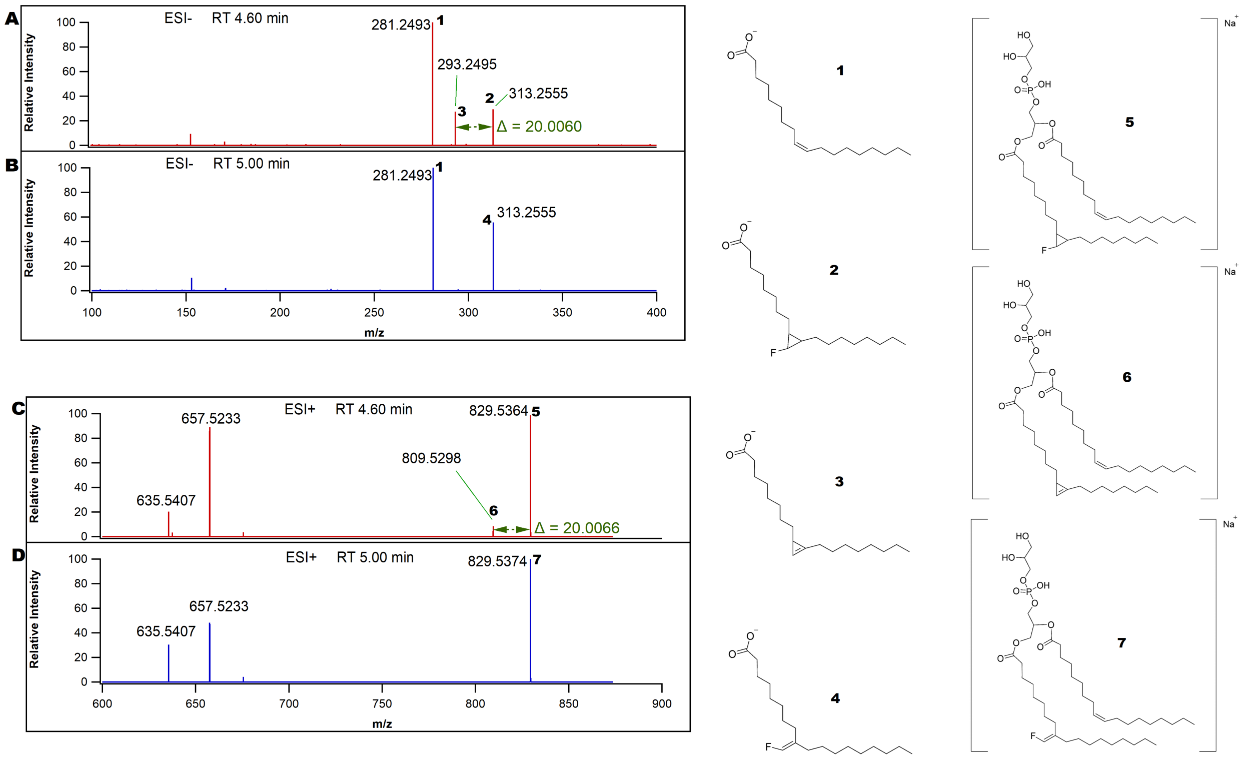

Figure S12. MSMS spectra of reaction products from the reaction catalyzed by CFAS between dOPG and FMeTeSAM. (**A**). Data-dependent MSMS spectrum of peak at 4.60 (ESI-, *m/z* 805.5432 parent ion) from *m/z* 100-400 collected with HCD fragmentation (nCE 35 eV). Fragment at *m/z* 281.2493 corresponds to unreacted oleyl fatty acid chain (**1**); fragment at *m/z* 313.2555 corresponds with oleyl chain with addition of CHF from FMeTeSAM to afford the fluorocyclopropane lipid (**2**). The fragment at *m/z* 293.2495 corresponds with the gas-phase hydrogen fluoride elimination cyclopropene product (**3**). (**B**). Data-dependent MSMS spectrum of peak at 5.00 (ESI-, *m/z* 805.5432 parent ion) from *m/z* 100-400 collected with HCD fragmentation (nCE 35 eV). Fragment at *m/z* 281.2493 corresponds to unreacted oleyl fatty acid chain (**1**); fragment at *m/z* 313.2555 corresponds with oleyl chain with addition of CHF from FMeTeSAM to afford a rearrangement product (**4**). (**C**). Data-dependent MSMS spectrum of peak at 4.60 (ESI+, *m/z* 829.5364 parent ion) from m/z 600-900 collected with HCD fragmentation (nCE 20 eV). Fragment at *m/z* 829.5364 corresponds to dOPG sodium adduct with addition of CHF from FMeTeSAM to afford the fluorocyclopropane lipid (**5**). The fragment at *m/z* 809.5298 corresponds to the gas-phase hydrogen fluoride elimination cyclopropene product (**6**). (**D**). Data-dependent MSMS spectrum of peak at 5.00 (ESI+, *m/z* 829.5374 parent ion) from *m/z* 600-900 collected with HCD fragmentation (nCE 20 eV). Fragment at *m/z* 829.5364 corresponds to dOPG sodium adduct with addition of CHF from FMeTeSAM to afford the rearrangement product (**7**). Position of the modifications are shown at the *sn*1 position but are present at either position.

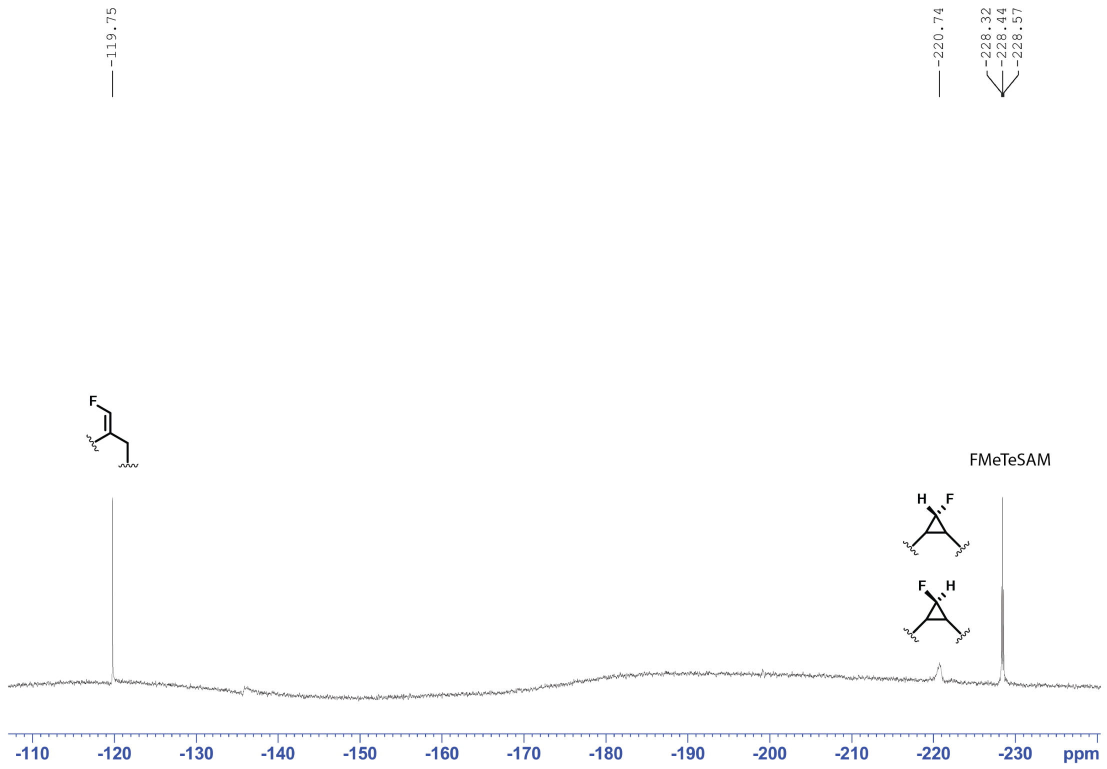

Figure S13. ^{19}F-NMR of CFA reaction. The reaction contains 50 mM Hepes pH 7.5, 20 μM CFAS, 5 mM dOPG, 700 μM FMeTeSAM, 5 mM ammonium bicarbonate, and 1.9 μM SAHN in a total volume of 600 uL. After incubating at 37 °C for 12h, the reaction mixture was transferred into an NMR tube. An insert tube containing 200 uL D_2O was used for locking the field. ^{19}F-NMR was collected on a 400 MHz spectrometer (376.4MHz ^{19}F) employing the spin echo sequence with the total echo time being set to 300 μs. ^{19}F-NMR was collected at 37 °C. Other experimental parameters are number of scans 25K, recycle delay 1s and spectral width 492ppm"

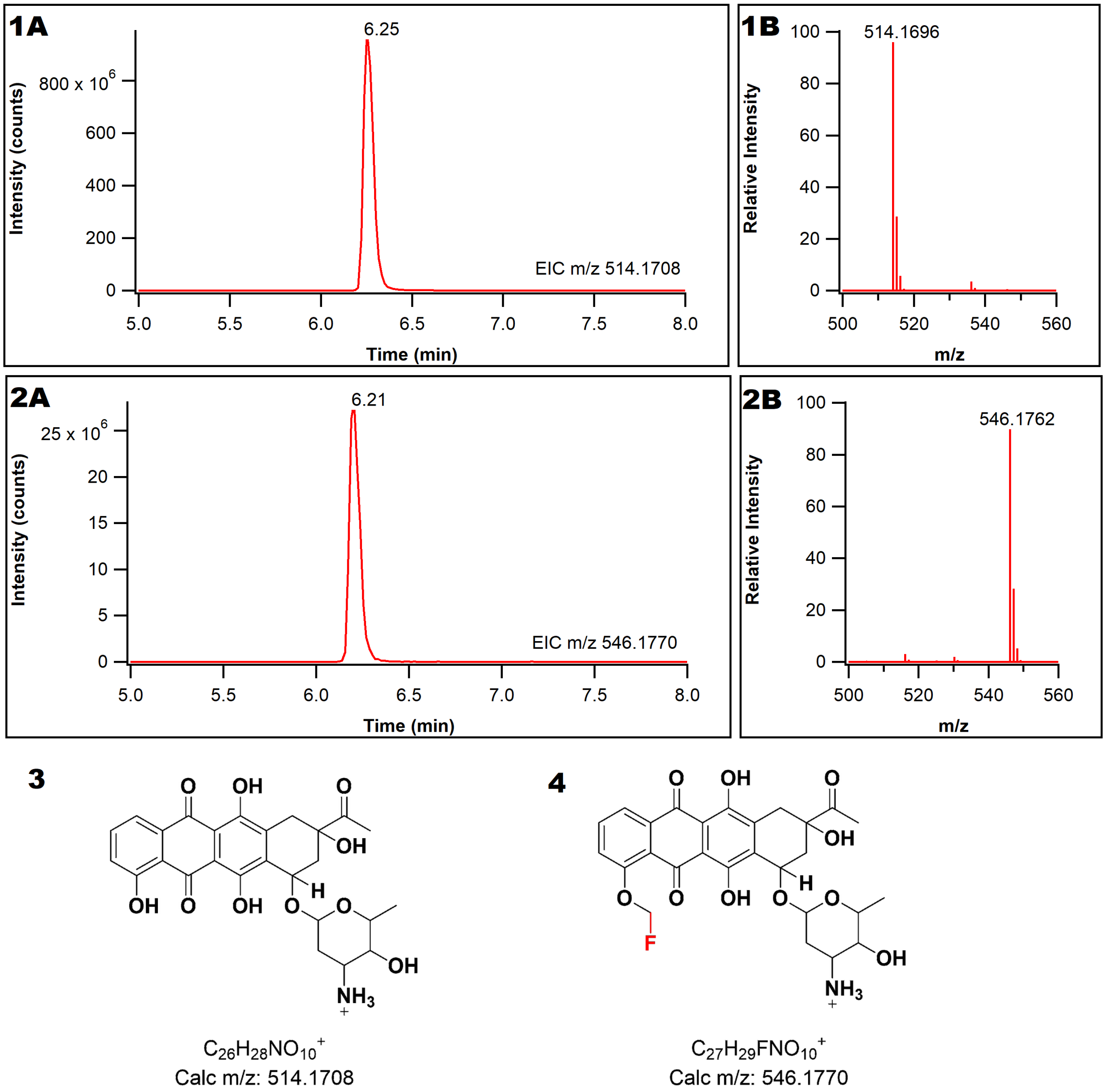

Figure S14. Reaction catalyzed by DnrK using FMeTeSAM as the methyl donor. **1A-1B**. EIC (ESI+) and HRMS of carminomycin (**3**) substrate of DnrK. **2A-2B**. EIC (ESI+) and HRMS of fluoromethylated product (**4**) from the DnrK reaction. A mass shift of 32.0066 was observed, consistent with a mass shift of HCF (32.0062).

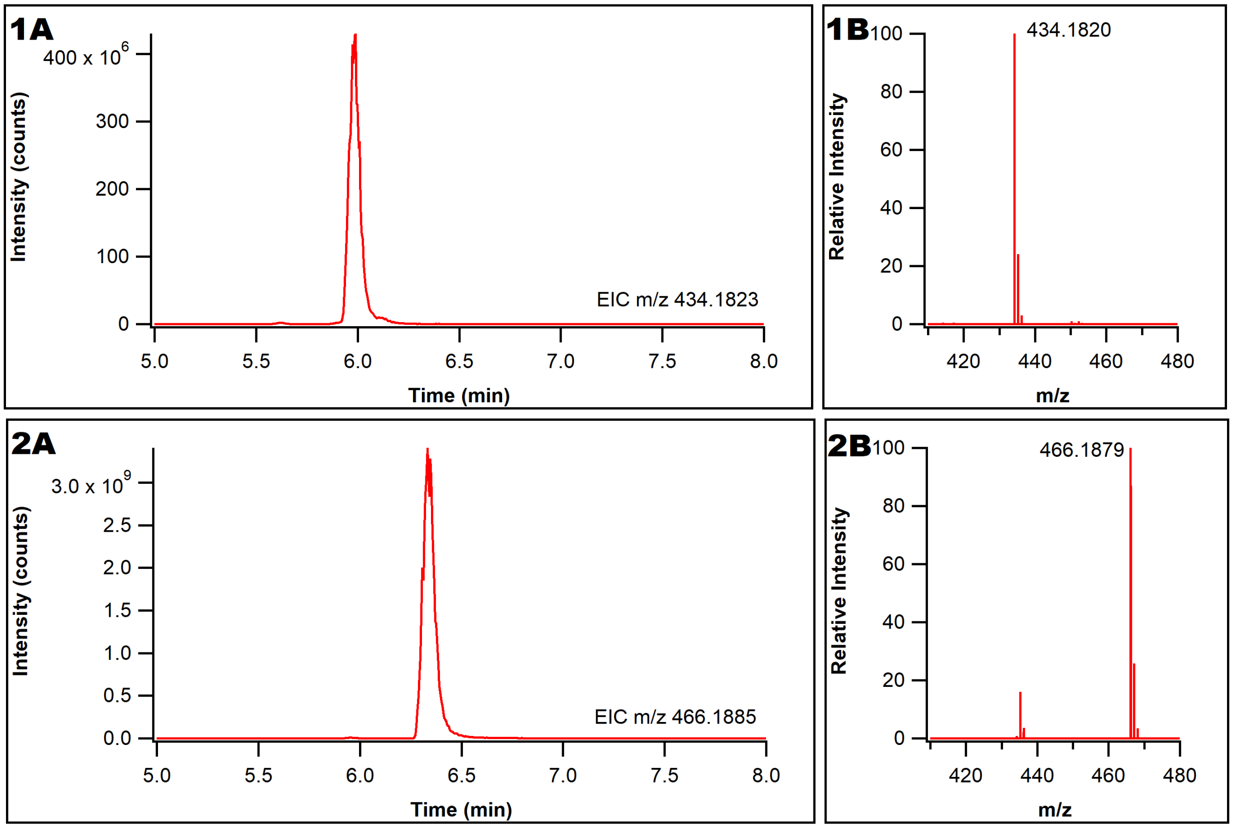

3

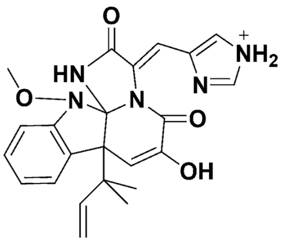

$C_{23}H_{24}N_5O_4^+$
Calc m/z: 434.1823

4

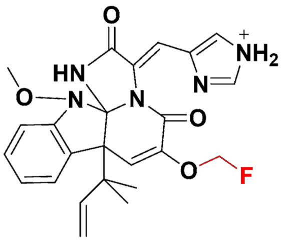

$C_{24}H_{25}FN_5O_4^+$
Calc m/z: 466.1885

Figure S15. Reaction catalyzed by OxaC using FMeTeSAM as the methyl donor. **1A-1B**. EIC (ESI+) and HRMS of meleagrin (**3**) substrate of OxaC. **2A-2B**. EIC (ESI+) and HRMS of fluoromethylated product (**4**) from the OxaC reaction. A mass shift of 32.0059 was observed, consistent with a mass shift of HCF (32.0062).

Experimental Procedures

Materials. All commercial materials were used as received unless otherwise noted. *N*-(2-Hydroxyethyl)-piperazine-*N*'-(2-ethanesulfonic acid) (HEPES), 2-amino-2-hydroxymethyl-1,3-propanediol (also known as tris(hydroxymethyl)aminomethane (Tris base)) were purchased from Fisher Scientific. Imidazole was purchased from J. T. Baker Chemical Co. Potassium chloride and glycerol were purchased from EMD Chemicals. 2-Mercaptoethanol, and *S*-adenosylhomocysteine (SAH) were purchased from MilliporeSigma. Kanamycin, ampicillin, Isopropyl β-D-1-thiogalactopyranoside (IPTG) and dithiothreitol (DTT) were purchased from Gold Biotechnology. EDTA-free protease-inhibitor cocktail tablets were purchased from Roche. Ni-NTA resin was purchased from Qiagen. Plasmid DNA isolation kits were purchased from Macherey-Nagel (Dueren, Germany). Tetrahydrofuran, dichloromethane, acetonitrile, and DMF were obtained from a JC Meyer solvent dispensing system. SiliaFlash 60 silica gel (230-400 mess) for flash chromatography was obtained from Silicycle Inc. SAM was synthesized and purified as described previously.[1] Carminomycin was purchased from MuseChem and Meleagrin was purchased from Cayman chemical. All other chemicals and materials were of the highest grade available and were from purchased from MilliporeSigma.

General Methods. UV-visible spectra were recorded on a Cary 50 spectrometer from Varian (Agilent Technologies, Santa Clara, CA) using the WinUV software package to control the instrument. High-performance liquid chromatography (HPLC) with detection by tandem mass spectrometry (LC-MS/MS) was conducted on an Agilent Technologies 1200 system coupled to an Agilent Technologies 6410 QQQ mass spectrometer. The system was operated with the associated MassHunter software package, which was also used for data collection and analysis. HPLC was also conducted on an Agilent 1100 Series system coupled to an Agilent 1100 Series variable

wavelength detector and quaternary pump. High resolution mass spectrometry (HRMS) was conducted on a Thermo Scientific Vanquish UPLC in line with a Q Exactive HF-X hybrid quadrupole-Orbitrap mass spectrometer. Data were collected and processed using Thermo Scientific Xcalibur 4.2.47. NMR spectra of all compounds were collected on a Bruker AV-3-HD-500 instrument. Kinetic data were fit with GraFit, and the error represented as standard deviation.

Codon-optimized gene and protein sequences

Codon optimized *Homo sapiens* COMT DNA

CATATGGGCGATACCAAAGAACAGCGTATTCTGAATCATGTTCTGCAGCATGCCGAA
CCGGGTAATGCACAGAGCGTTCTGGAAGCAATTGATACCTATTGTGAACAGAAAGA
ATGGGCCATGAATGTGGGTGATAAAAAGGGTAAAATTGTGGGTGCCGTGATTCAAG
AACATCAGCCGTTTGTTCTGCTGGAACTGGGTGCATATTGTGGTTATAGCGCAGTTC
GTATGGCACGTCTGCTGAGTCCGGGTGCACGTCTGATTACCATTGAAATTAACCCGG
ATTGTGCAGCAATTACCCAGCGTATGGTTGATTTTGCCGGTGTTAAAGATAAAGTTA
CCCTGGTTGTTGGTGCAAGCCAGGATATTATTCCGCAGCTGAAAAAGAAATATGATG
TGGATACCCTGGATATGGTGTTTCTGGATCATTGGAAAGATCGTTATCTGCCGGATA
CACTGCTGCTGGAAGAATGTGGTCTGCTGCGTCGTGGCACCGTGCTGCTGGCAGATA
ATGTTATTTGTCCTGGTGCACCGGATTTTCTGGCACATGTTCGTGGTAGCAGCTGTTT
TGAATGTACCCATTATCAGTCCTTTCTGGAATATCGTGAAGTTGTTGATGGTCTGGA
AAAGGCCATCTATAAAGGTCCGGGTAGCGAAGCAGGTCCGTAAGAATTC

*Nde*I
Stop
EcoRI

COMT Protein Sequence with N-terminal His$_6$-tag

MGSSHHHHHHSSGLVPRGSHMGDTKEQRILNHVLQHAEPGNAQSVLEAIDTYCEQKEW
AMNVGDKKGKIVGAVIQEHQPFVLLELGAYCGYSAVRMARLLSPGARLITIEINPDCAAI
TQRMVDFAGVKDKVTLVVGASQDIIPQLKKKYDVDTLDMVFLDHWKDRYLPDTLLLE
ECGLLRRGTVLLADNVICPGAPDFLAHVRGSSCFECTHYQSFLEYREVVDGLEKAIYKG
PGSEAGP

Codon optimized *Homo sapiens* PNMT DNA

CATATGAGCGGTGCAGATCGTAGCCCGAATGCCGGTGCAGCTCCGGATAGCGCACC
TGGTCAGGCAGCAGTTGCAAGCGCATATCAGCGTTTTGAACCGCGTGCATATCTGCG
TAATAACTATGCACCGCCTCGTGGTGATCTGTGTAATCCGAATGGTGTTGGTCCGTG
GAAACTGCGTTGTCTGGCACAGACCTTTGCAACCGGTGAAGTTAGCGGTCGTACCCT
GATTGATATTGGTAGCGGTCCGACCGTTTATCAGCTGCTGAGCGCATGTAGCCATTT
TGAAGATATTACCATGACCGACTTTCTGGAAGTGAATCGTCAAGAATTAGGTCGTTG
GCTGCAAGAGGAACCGGGTGCATTTAATTGGAGCATGTATAGCCAGCATGCATGTCT
GATTGAAGGTAAAGGTGAATGTTGGCAGGATAAAGAACGTCAGCTGCGTGCACGTG
TTAAACGTGTTCTGCCGATTGATGTTCATCAGCCGCAGCCGTTAGGTGCAGGTAGTC
CGGCACCGCTGCCTGCAGATGCACTGGTTAGCGCATTTTGTCTGGAAGCAGTTAGTC
CGGATCTGGCAAGCTTTCAGCGTGCACTGGATCATATTACCACACTGCTGCGTCCTG
GTGGTCATCTGCTGCTGATTGGTGCCCTGGAAGAAAGCTGGTATCTGGCAGGCGAAG
CACGTCTGACCGTTGTTCCGGTTAGCGAAGAAGAAGTTCGCGAAGCACTGGTTCGTA
GCGGTTATAAAGTTCGTGATCTGCGTACCTATATTATGCCTGCACATCTGCAGACCG
GTGTTGATGATGTTAAAGGTGTTTTCTTTGCATGGGCACAGAAAGTTGGTCTGTAAG
AATTC

NdeI
Stop
EcoRI

PNMT Protein Sequence with N-terminal His$_6$-tag

MGSSHHHHHHSSGLVPRGSHMSGADRSPNAGAAPDSAPGQAAVASAYQRFEPRAYLR
NNYAPPRGDLCNPNGVGPWKLRCLAQTFATGEVSGRTLIDIGSGPTVYQLLSACSHFEDI
TMTDFLEVNRQELGRWLQEEPGAFNWSMYSQHACLIEGKGECWQDKERQLRARVKRV
LPIDVHQPQPLGAGSPAPLPADALVSAFCLEAVSPDLASFQRALDHITTLLRPGGHLLLIG
ALEESWYLAGEARLTVVPVSEEEVREALVRSGYKVRDLRTYIMPAHLQTGVDDVKGVF
FAWAQKVGL

Codon optimized Human TPMT DNA

CATATGGATGGCACCCGTACCAGCCTGGATATTGAAGAATATAGCGATACCGAAGT
GCAGAAAAATCAGGTTCTGACCCTGGAAGAATGGCAGGATAAATGGGTTAATGGTA
AAACCGCCTTTCATCAAGAACAGGGTCATCAGCTGCTGAAAAACATCTGGATACCT

TTCTGAAAGGCAAAAGCGGTCTGCGTGTTTTTTTCCGCTGTGTGGTAAAGCAGTTG
AGATGAAATGGTTTGCAGATCGTGGTCATAGCGTTGTTGGTGTTGAAATTAGCGAAC
TGGGTATCCAAGAATTTTTCACCGAACAGAATCTGAGCTATAGCGAAGAACCGATTA
CCGAAATTCCGGGTACAAAAGTGTTTAAAAGCAGCAGCGGTAACATTAGCCTGTATT
GTTGTAGCATTTTTGATCTGCCTCGTACCAACATCGGCAAATTTGATATGATTTGGGA
TCGTGGTGCACTGGTTGCAATTAATCCGGGTGATCGTAAATGTTATGCCGATACCAT
GTTTAGCCTGCTGGGCAAAAAATTCCAGTATCTGCTGTGTGTGCTGAGCTATGATCC
GACCAAACATCCGGGTCCGCCTTTTTATGTTCCGCATGCAGAAATTGAACGCCTGTT
TGGTAAAATTTGCAATATTCGCTGCCTGGAAAAAGTGGATGCATTTGAAGAACGTCA
TAAAAGCTGGGGTATTGATTGCCTGTTTGAGAAACTGTACCTGCTGACCGAAAAATA
AGAATTC

NdeI
Stop
EcoRI

TPMT Protein Sequence with N-terminal His$_6$-tag

MGSSHHHHHHSSGLVPRGSHMDGTRTSLDIEEYSDTEVQKNQVLTLEEWQDKWVNGK
TAFHQEQGHQLLKKHLDTFLKGKSGLRVFFPLCGKAVEMKWFADRGHSVVGVEISELG
IQEFFTEQNLSYSEEPITEIPGTKVFKSSSGNISLYCCSIFDLPRTNIGKFDMIWDRGALVAI
NPGDRKCYADTMFSLLGKKFQYLLCVLSYDPTKHPGPPFYVPHAEIERLFGKICNIRCLE
KVDAFEERHKSWGIDCLFEKLYLLTEK

Codon optimized *Streptomyces peucetius* DnrK DNA

CATATGACCGCAGAACCGACCGTTGCAGCACGTCCGCAGCAGATTGATGCACTGCG
TACCCTGATTCGTCTGGGTAGCCTGCATACCCCGATGGTTGTTCGTACCGCAGCAAC
CCTGCGTCTGGTTGATCATATTCTGGCAGGCGCACGTACCGTTAAAGCACTGGCAGC
ACGTACAGATACCCGTCCGGAAGCACTGCTGCGTCTGATTCGCCATCTGGTTGCAAT
TGGTCTGCTGGAAGAGGATGCACCGGGTGAATTTGTTCCGACCGAAGTTGGTGAACT
GCTGGCAGATGACCATCCGGCAGCACAGCGTGCATGGCATGATCTGACCCAGGCAG
TTGCACGTGCCGATATTAGCTTTACCCGTCTGCCTGATGCAATTCGTACCGGTCGTCC
GACCTATGAAAGCATTTATGGTAAACCGTTTTATGAGGATCTGGCAGGTCGTCCGGA
TCTGCGTGCAAGCTTTGATAGCCTGCTGGCATGTGATCAGGATGTTGCATTTGATGC
CCCTGCAGCAGCCTATGATTGGACCAATGTTCGTCATGTTCTGGATGTTGGTGGTGG
TAAAGGTGGTTTTGCAGCAGCAATTGCACGTCGTGCACCGCATGTTAGCGCAACCGT
TCTGGAAATGGCAGGCACCGTTGATACCGCACGTAGCTATCTGAAAGATGAAGGTC
TGAGCGATCGTGTTGATGTTGTTGAAGGTGATTTTTTTGAACCGCTGCCTCGTAAAG
CAGATGCAATTATTCTGAGCTTTGTTCTGCTGAATTGGCCTGATCATGATGCCGTTCG

TATTCTGACCCGTTGTGCCGAAGCACTGGAACCTGGTGGTCGCATTCTGATTCATGA
ACGTGATGATCTGCATGAGAACAGCTTTAATGAACAGTTTACCGAACTGGATCTGCG
CATGCTGGTTTTTTAGGTGGTGCCCTGCGCACCCGTGAAAAATGGGATGGTCTGGC
AGCAAGCGCAGGTCTGGTTGTGGAAGAAGTTCGTCAGCTGCCGAGTCCGACCATTCC
GTATGATCTGAGTCTGCTGGTTCTGGCACCGGCAGCAACCGGTGCATAACTCGAG

NdeI
Stop
XhoI

dwpSUMO-pET28(+)-DnrK protein sequence

MGHHHHHHGSDSEVNQEAKPEVKPEVKPETHINLKVSDGSSEIFFKIKKTTPLRRLMEAF
AKRQGKEMDSLRFLYDGIRIQADQAPEDLDMEDNDIIEAHREQIGGGHMTAEPTVAARP
QQIDALRTLIRLGSLHTPMVVRTAATLRLVDHILAGARTVKALAARTDTRPEALLRLIRH
LVAIGLLEEDAPGEFVPTEVGELLADDHPAAQRAWHDLTQAVARADISFTRLPDAIRTG
RPTYESIYGKPFYEDLAGRPDLRASFDSLLACDQDVAFDAPAAAYDWTNVRHVLDVGG
GKGGFAAAIARRAPHVSATVLEMAGTVDTARSYLKDEGLSDRVDVVEGDFFEPLPRKA
DAIILSFVLLNWPDHDAVRILTRCAEALEPGGRILIHERDDLHENSFNEQFTELDLRMLVF
LGGALRTREKWDGLAASAGLVVEEVRQLPSPTIPYDLSLLVLAPAATGA

dwpSUMO tag has been cleaved by Ulp1 protease as described in materials and methods.

***E. coli* TrmD was amplified from *E. coli* genomic DNA using the primers containing NdeI and XhoI restriction sites as shown below and cloned into dwpSUMO-pET-28a(+) vector containing the same restriction sites.**

Forward primer:

5'-3': CGGAAACATATGTGGATTGGCATAATTAG

Reverse primer:

5'-3': CGGAAACTCGAGTTACGCCATCCCATCATGTT

DNA sequence of *Ec*TrmD

ATGGGTCATCACCATCATCATCACGGGTCGGACTCAGAAGTCAATCAAGAAGCTAA
GCCAGAGGTCAAGCCAGAAGTCAAGCCTGAGACTCACATCAATTTAAAGGTGTCCG

ATGGATCTTCAGAGATCTTCTTCAAGATCAAAAGACCACTCCTTTAAGAAGGCTGA
TGGAAGCGTTCGCTAAAAGACAGGGTAAGGAAATGGACTCCTTAAGATTCTTGTAC
GACGGTATTAGAATTCAAGCTGATCAGGCCCCTGAAGATTTGGACATGGAGGATAA
CGATATTATTGAGGCTCACCGCGAACAGATTGGAGGTGGCCATATGTGGATTGGCAT
AATTAGCCTGTTTCCTGAAATGTTCCGCGCAATTACCGATTACGGGGTAACTGGCCG
GGCAGTTAAAAATGGCCTGCTGAGCATCCAGAGCTGGAGTCCTCGCGACTTCACGC
ATGACCGGCACCGTACCGTGGACGATCGTCCTTACGGCGGCGGACCGGGGATGTTA
ATGATGGTGCAACCCTTGCGGGACGCCATTCATGCAGCAAAAGCCGCGGCGGGTGA
AGGCGCAAAGGTGATTTATCTGTCACCACAGGGACGCAAGCTTGATCAAGCGGGCG
TCAGCGAACTGGCAACGAATCAAAAATTGATTCTGGTGTGCGGTCGCTACGAAGGT
ATAGATGAGCGCGTGATCCAAACCGAAATTGACGAAGAATGGTCAATCGGCGATTA
CGTTCTCAGTGGTGGTGAGTTACCAGCAATGACGCTGATTGACTCCGTTTCCCGGTTT
ATTCCGGGAGTACTGGGACATGAAGCCTCGGCAACGGAAGATTCCTTTGCTGAAGG
ATTGCTGGATTGCCCGCACTATACGCGGCCTGAGGTGTTAGAAGGGATGGAAGTTCC
GCCAGTGTTACTGTCGGGCAACCATGCCGAGATACGTCGCTGGCGTTTGAAACAGTC
GCTGGGCCGTACCTGGCTTAGAAGACCTGAACTTCTGGAAAACCTGGCTCTGACTGA
AGAGCAAGCAAGGTTGCTGGCGGAGTTCAAAACGGAACACGCACAACAGCAACAT
AAACATGATGGGATGGCGTAACTCGAG

dwpSUMO tag
NdeI
XhoI

dwpSUMO-pET28(+)-TrmD protein sequence

MGHHHHHHGSDSEVNQEAKPEVKPEVKPETHINLKVSDGSSEIFFKIKKTTPLRRLMEAF
AKRQGKEMDSLRFLYDGIRIQADQAPEDLDMEDNDIIEAHREQIGGGHMWIGIISLFPEM
FRAITDYGVTGRAVKNGLLSIQSWSPRDFTHDRHRTVDDRPYGGGPGMLMMVQPLRD
AIHAAKAAAGEGAKVIYLSPQGRKLDQAGVSELATNQKLILVCGRYEGIDERVIQTEIDE
EWSIGDYVLSGGELPAMTLIDSVSRFIPGVLGHEASATEDSFAEGLLDCPHYTRPEVLEG
MEVPPVLLSGNHAEIRRWRLKQSLGRTWLRRPELLENLALTEEQARLLAEFKTEHAQQQ
HKHDGMA-

dwpSUMO tag has been cleaved by Ulp1 protease as described in materials and methods.

Codon optimized pET28a(+)-NNMT was a generous gift from the Thompson group at the University of Massachusetts Medical School, USA.

Codon optimized pET28a(+)-SgvM was a generous gift from the Mueller group at the University of Freiburg, Germany.

Codon optimized pET-26b(+)-NovO was a generous gift from the Burley group at the University of Strathclyde, Glasgow, UK.

Codon optimized pET-28a(+)-OxaC was a generous gift from the Sherman group at the University of Michigan, Ann Arbor, USA.

Overexpression and purification of catechol *O*-methyltransferase (COMT). Gene encoding human COMT was codon-optimized using GeneArt software (Thermo Fisher) for expression in *E. coli.* The gene was subcloned into pET-28a(+) (NE Biolabs) vector using *Nde*I and *Eco*RI restriction sites. The resulting plasmid pET-28a(+)-COMT was used to transform *E. coli* BL21 (DE3) competent cells. Starting from a single colony, a 200 mL starter culture containing 50 mg/L kanamycin was shaken at 37 °C and 250 rpm for 12 h. 10 mL of the starter culture was used to inoculate 4 L of LB medium containing 50 mg/L kanamycin and incubated at 37 °C and 180 rpm until an optical density at 600 nm (OD_{600}) of 0.5 to 0.7 was reached. Protein expression was induced by addition of isopropylthio-β-D-galactoside (IPTG) to a final concentration of 0.2 mM. The culture was incubated at 18 °C and 180 rpm for an additional 18 h. The cells were harvested by centrifugation (4 °C, 6000 × *g*, 15 min), flash-frozen in liquid nitrogen and stored at -80 °C until use.

For protein purification, the cells were resuspended in 140 mL lysis buffer (50 mM Tris-HCl, 300 mM NaCl, pH 8.0) containing a protease-inhibitor cocktail tablet, lysozyme (75 mg) and DNaseI (10 mg), and the resulting suspension was disrupted by sonication with an ultrasonic cell disruptor (Branson Sonifier II "Modell W- 250", Heinemann). The lysates were then clarified by centrifugation (4 °C, 45000 × *g*, 30 min). N-terminally His_6-tagged COMT was purified via Ni-NTA affinity chromatography. The Ni-NTA resin was pre-equilibrated with lysis buffer. After

loading the supernatant onto the Ni-NTA column, the resin was washed with 120 mL wash buffer (50 mM Tris-HCl, 300 mM NaCl, 20 mM imidazole, pH 8.0) before eluting the protein with 50 mL of elution buffer (50 mM Tris-HCl, 300 mM NaCl, 250 mM imidazole, pH 8.0). The protein was concentrated using Amicon® Ultra Centrifugal Filter (Millipore, 10K MWCO) and buffer exchanged into the storage buffer (50 mM Tris-HCl, 300 mM NaCl, 2 mM $MgCl_2$, 10 mM β-mercaptoethanol, 20% glycerol (v/v) pH 8.0) using a PD-10 column (GE Healthcare). The enzyme solution was snap-frozen in liquid N_2 and stored at −80 °C until use.

Overexpression and purification of phenylethanolamine *N*-methyltransferase (PNMT). Gene encoding human PNMT was codon-optimized using GeneArt software (Thermo Fisher) for expression in *E. coli.* The gene was sub-cloned into pET-28a(+) (NE Biolabs) vector using *Nde*I and *Eco*RI restriction sites. The resulting plasmid pET-28a(+)-PNMT was used for expression and purification and performed as described for COMT.

Overexpression and purification of nicotinamide *N*-methyltransferase (NNMT). A plasmid encoding N-terminally His_6-tagged NNMT in a pET-28a(+) vector was a generous gift from the Thompson group at the University of Massachusetts, USA. *E. coli* BL21 (DE3) was transformed with the plasmid pET-28a(+)-NNMT. Starting from a single colony, a 200 mL starter culture containing 50 mg/L kanamycin was shaken at 37 °C and 250 rpm for 12 h. 10 mL of the starter culture was used to inoculate 4 L of LB medium containing 50 mg/L kanamycin. The culture was incubated at 37 °C and 180 rpm until an optical density at 600 nm (OD_{600}) of ~0.6 was reached. Protein expression was induced by addition of IPTG to a final concentration of 1 mM, and incubation was continued at 18 °C at 180 rpm for an additional 18 h. The cells were harvested by centrifugation (4 °C, 6000 × *g*, 15 min), flash-frozen in liquid nitrogen and stored at -80 °C until

use. Purification of the protein was performed as described for COMT with the exception that the wash buffer contained 40 mM imidazole. The protein was concentrated using Amicon® Ultra Centrifugal Filter (Millipore, 10K MWCO) and buffer exchanged into the storage buffer (50 mM Tris-HCl, 300 mM NaCl, 10% glycerol (v/v), pH 8.0) using a PD-10 column (GE Healthcare). The enzyme solution was snap-frozen in liquid N_2 and stored at −80 °C until use.

Overexpression and purification of G37 guanine RNA methylase (TrmD). TrmD was amplified from *E. coli* genomic DNA using primers (described above) containing *Nde*I and *Xho*I restriction sites and cloned into dwpSUMO-pET-28a(+) using *Nde*I and *Xho*I restriction sites to yield dwpSUMO-pET-28a(+)-TrmD. *E. coli* BL21 (DE3) was transformed with plasmid dwpSUMO-pET-28a(+)-TrmD. Starting from a single colony, a 200 mL starter culture containing 50 mg/L kanamycin was shaken at 37 °C and 250 rpm for 12 h. 10 mL of the starter culture was used to inoculate 3 L of LB medium containing 50 mg/L kanamycin and incubated at 37 °C and 180 rpm until an optical density at 600 nm (OD_{600}) of ~0.6 was reached. Protein expression was induced by addition of IPTG to a final concentration of 0.5 mM. The culture was incubated at 18 °C and 180 rpm for an additional 18 h. The cells were harvested by centrifugation (4 °C, 6000 × *g*, 15 min), flash-frozen in liquid nitrogen and stored at -80 °C until use.

For protein purification, the cells were resuspended in 140 mL lysis buffer (50 mM Tris-HCl, 300 mM NaCl, 10 mM imidazole, 2 mM β–mercaptoethanol (βME), 10% (v/v) glycerol, pH 8.0) containing a protease-inhibitor cocktail tablet, lysozyme (75 mg) and DNaseI (10 mg), and the resulting suspension was disrupted by sonication with an ultrasonic cell disruptor (Branson Sonifier II "Modell W- 250", Heinemann). The lysate was clarified by centrifugation (4 °C, 45000 × *g*, 30 min). The N-terminally His_6-SUMO-tagged TrmD was purified via Ni-NTA affinity chromatography. The Ni-NTA resin was pre-equilibrated with 150 mL of lysis buffer. After

loading supernatant onto the Ni-NTA column, the resin was washed with 120 mL of wash buffer (50 mM Tris-HCl, 300 mM NaCl, 30 mM imidazole, 2 mM βME, 5% (v/v) glycerol, pH 8.0). The protein was eluted from the Ni-NTA column with 50 mL of elution buffer (50 mM Tris-HCl, 300 mM NaCl, 300 mM imidazole, 2 mM βME, 5% (v/v) glycerol, pH 8.0) and concentrated using Amicon® Ultra Centrifugal Filter (Millipore, 10K MWCO). Buffer exchange was performed on the concentrated protein with wash buffer using a PD-10 column (GE Healthcare). Ulp1 protease (50 µg per mg of protein to be cleaved) was added to the His_6-SUMO-tagged protein to cleave the His_6-SUMO tag and the reaction mixture was incubated on ice overnight. The protein mixture was then reloaded onto the Ni-NTA column which was pre-equilibrated in wash buffer. The protein was collected in flow-through, concentrated and buffer exchanged into storage buffer (50 mM Tris-HCl, 300 mM NaCl, 1 mM β-mercaptoethanol, 10% glycerol (v/v) pH 8.0). The enzyme solution was snap-frozen using liquid N_2 and stored at −80 °C until use.

Overexpression and purification of thiopurine methyltransferase (TPMT). Gene encoding human TPMT was codon-optimized using GeneArt software (Thermo Fisher) for expression in *E. coli.* The gene was sub-cloned into pET-28a(+) (NE Biolabs) vector using *Nde*I and *Eco*RI restriction sites. The resulting plasmid pET-28a(+)-TPMT was used to transform *E. coli* BL21 (DE3) competent cells. Starting from a single colony, a 200 mL starter culture containing 50 mg/L kanamycin was shaken at 37 °C and 250 rpm for 12 h. 10 mL of an overnight culture was used to inoculate 4 L of LB medium containing 50 mg/L kanamycin and incubated at 37 °C and 180 rpm until an optical density at 600 nm (OD_{600}) of ~0.8 was reached. Protein expression was induced by adding IPTG to a final concentration of 0.4 mM, and incubation was continued at 24 °C and 180 rpm for an additional 18 h. The cells were harvested by centrifugation (4 °C, 6000 × *g*, 15 min), flash-frozen in liquid nitrogen and stored at -80 °C until use.

For protein purification, the cells were resuspended in 140 mL lysis buffer (50 mM Tris-HCl, 300 mM NaCl, 10 mM imidazole, 10 % glycerol (v/v), pH 7.9) containing a protease-inhibitor cocktail tablet, lysozyme (75 mg) and DNaseI (10 mg), and the resulting suspension was disrupted by sonication with an ultrasonic cell disruptor (Branson Sonifier II "Modell W- 250", Heinemann). The lysates were clarified by centrifugation (4 °C, 45000 × *g*, 30 min). N-terminally His_6-tagged TPMT was purified by Ni-NTA affinity chromatography. The Ni-NTA resin was pre-equilibrated with 150 mL of lysis buffer. After loading supernatant onto the Ni-NTA column, the resin was washed with 120 mL of wash buffer (50 mM Tris-HCl, 300 mM NaCl, 30 mM imidazole, 5 mM βME, pH 7.9). The protein bound to the Ni-NTA resin was eluted with 50 mL of elution buffer (50 mM Tris-HCl, 300 mM NaCl, 250 mM imidazole, 5 mM βME, pH 7.9). The protein was concentrated using Amicon® Ultra Centrifugal Filter (Millipore, 10K MWCO) and buffer exchanged into the storage buffer (50 mM Tris-HCl, 2 mM DTT, 20% glycerol (v/v), pH 7.9) using a PD-10 column (GE Healthcare). The enzyme solution was snap-frozen using liquid nitrogen and stored at −80 °C until use.

Overexpression and purification of SgvM. A plasmid containing the *SgvM* gene in pET-28a(+) vector containing the *N*-terminal His_6-tag was a generous gift from the Mueller group at the University of Freiburg, Germany. *E. coli* BL21 (DE3) was transformed with the plasmid pET-28a(+)-SgvM. Starting from a single colony, a 200 mL starter culture containing 50 mg/L kanamycin was shaken at 37 °C and 250 rpm for 12 h. 15 mL of starter culture was used to inoculate 3 L of LB medium containing 50 mg/L kanamycin and incubated at 37 °C and 180 rpm until an optical density at 600 nm (OD_{600}) of ~0.6 was reached. Protein expression was induced by addition of IPTG to a final concentration of 0.2 mM. The culture was incubated at 18 °C and 180 rpm for an additional 18 h. The cells were harvested by centrifugation (4 °C, 6000 × *g*, 15 min),

flash-frozen in liquid nitrogen and stored at -80 °C until use.

For protein purification, the cells were resuspended in 140 mL lysis buffer (50 mM Tris-HCl, 300 mM NaCl, pH 8.0) containing a protease-inhibitor cocktail tablet, lysozyme (75 mg) and DNaseI (10 mg), and the resulting suspension was disrupted by sonication with an ultrasonic cell disruptor (Branson Sonifier II "Modell W- 250", Heinemann). The lysates were then clarified by centrifugation (4 °C, 45000 × *g*, 30 min). N-terminally His_6-tagged SgvM was purified via Ni-NTA affinity chromatography. The Ni-NTA resin was pre-equilibrated with 150 mL of lysis buffer. After loading supernatant onto the Ni-NTA column, the resin was washed with 120 mL of wash buffer (50 mM Tris-HCl, 300 mM NaCl, 20 mM imidazole, pH 8.0), followed by protein elution from the Ni-NTA resin with 50 mL of elution buffer (50 mM Tris-HCl, 300 mM NaCl, 250 mM imidazole, pH 8.0). The protein was concentrated using Amicon® Ultra Centrifugal Filter (Millipore, 10K MWCO) and buffer exchanged into the storage buffer (50 mM Tris-HCl, 300 mM NaCl, 10% glycerol (v/v) pH 8.0) using a PD-10 column (GE Healthcare). The enzyme solution was snap-frozen using liquid nitrogen and stored at −80 °C until use.

Overexpression and purification of NovO. A plasmid containing the *novO* gene in pET-26b(+) was a generous gift from the Burley group at the University of Strathclyde, Glasgow, UK. *E. coli* BL21 (DE3) was transformed with plasmid pET-26b(+)-NovO. Starting from a single colony, a 200 mL starter culture containing 50 mg/L kanamycin was shaken at 37 °C and 250 rpm for 12 h. 10 mL of an overnight culture was used to inoculate 3 L of LB medium containing 50 mg/L kanamycin and incubated at 37 °C and 250 rpm until an optical density at 600 nm (OD_{600}) of ~0.6 was reached. Protein expression was induced by addition of IPTG to a final concentration of 0.3 mM. The culture was incubated at 18 °C and 180 rpm for an additional 18 h. The cells were harvested by centrifugation (4 °C, 6000 × *g*, 15 min), flash-frozen in liquid nitrogen and stored at

-80 °C until use.

For protein purification, the cells were resuspended in 140 mL lysis buffer (100 mM Tris-HCl, 150 mM NaCl, 10 mM imidazole, pH 8.0) containing a protease-inhibitor cocktail tablet, lysozyme (75 mg) and DNaseI (10 mg), and the resulting suspension was disrupted by sonication with an ultrasonic cell disruptor (Branson Sonifier II "Modell W- 250", Heinemann). The lysates were clarified by centrifugation (4 °C, 45000 × *g*, 30 min). C-terminally His_6-tagged NovO was purified via Ni-NTA affinity chromatography. The Ni-NTA resin was pre-equilibrated with 150 mL of lysis buffer. After loading supernatant onto the Ni-NTA column, the resin was washed with 120 mL of wash buffer (100 mM Tris-HCl, 150 mM NaCl, 20 mM imidazole, pH 8.0), followed by protein elution with 50 mL of elution buffer (100 mM Tris-HCl, 150 mM NaCl, 250 mM imidazole, pH 8.0). The protein was concentrated using Amicon® Ultra Centrifugal Filter (Millipore, 10K MWCO) and buffer exchanged into the storage buffer (100 mM Tris-HCl, 150 mM NaCl, 10% glycerol pH 8.0) using a PD-10 column (GE Healthcare). The enzyme solution was snap-frozen using liquid nitrogen and stored at −80 °C until use.

Overexpression and purification of OxaC. A plasmid containing the *oxaC* gene in pET-28a(+) was a generous gift from Sherman group at the University of Michigan. *E. coli* BL21 (DE3) was transformed with the plasmid pET-28a(+)-OxaC. Enzyme expression and purification of OxaC were performed according to the procedure outlined by Sherman & coworkers with a minor modification.[2] We used β-mercaptoethanol (βME) as a reducing agent during protein purification instead of TCEP.

Overexpression and purification of DnrK. Gene encoding *Streptomyces peucetius* DnrK was codon-optimized using GeneArt software (Thermo Fisher) for expression in *E. coli.* The gene

was sub-cloned into dwpSUMO-pET-28a(+) (NE Biolabs) vector using *Nde*I and *Xho*I restriction sites. The resulting plasmid dwpSUMO-pET-28a(+)-DnrK was used to transform *E. coli* BL21 (DE3) competent cells. Starting from a single colony, a 200 mL starter culture containing 50 mg/L kanamycin was shaken at 37 °C and 250 rpm for 12 h. 10 mL of an overnight culture was used to inoculate 3 L of LB medium containing 50 mg/L kanamycin and incubated at 37 °C and 180 rpm until an optical density at 600 nm (OD_{600}) of ~0.6 was reached. Protein expression was induced by addition of IPTG to a final concentration of 0.25 mM. The culture was incubated at 18 °C and 180 rpm for an additional 18 h. The cells were harvested by centrifugation (4 °C, 6000 × *g*, 15 min), flash-frozen in liquid nitrogen and stored at -80 °C until use.

For protein purification, the cells were resuspended in 140 mL lysis buffer (50 mM Tris-HCl, 250 mM NaCl, 10 mM imidazole, 2 mM βME, pH 8.0) containing a protease-inhibitor cocktail tablet, lysozyme (75 mg) and DNaseI (10 mg), and the resulting suspension was disrupted by sonication with an ultrasonic cell disruptor (Branson Sonifier II "Modell W- 250", Heinemann). The lysates were clarified by centrifugation (4 °C, 45000 × *g*, 30 min). N-terminally His_6-tagged DnrK was purified via Ni-NTA affinity chromatography. The Ni-NTA resin was pre-equilibrated with 150 mL of lysis buffer. After loading supernatant onto the Ni-NTA column, the resin was washed with 120 mL of wash buffer (50 mM Tris-HCl, 250 mM NaCl, 30 mM imidazole, 2 mM βME, pH 8.0), followed by elution of the protein from the Ni-NTA resin with 50 mL of elution buffer (50 mM Tris-HCl, 250 mM NaCl, 250 mM imidazole, 2 mM βME, pH 8.0). The protein was concentrated using Amicon® Ultra Centrifugal Filter (Millipore, 10K MWCO). Buffer exchange was performed on the concentrated protein with wash buffer using a PD-10 column (GE Healthcare). Ulp1 protease (50 μg per mg of protein to be cleaved) was added to the His_6-SUMO-tagged protein to cleave the His_6-SUMO tag, and the reaction mixture was incubated on ice

overnight. The protein mixture was then reloaded onto the Ni-NTA column which was pre-equilibrated in wash buffer. The protein was collected in flow-through, concentrated and buffer exchanged into storage buffer (50 mM Tris-HCl, 250 mM NaCl, 10% glycerol, 2 mM βME, pH 8.0). The enzyme solution was snap-frozen using liquid nitrogen and stored at −80 °C until use.

Kinetic assays of COMT and quantification of products by LC-MS.

General procedure for COMT kinetic assays: Reactions were conducted in a volume of 30 µL and contained 50 mM HEPES pH 7.5, 0.3 mM DHBA,5 mM $MgCl_2$, and 100 µM Niacin as an internal standard with varying concentrations of SAM or SAM analog. Enzyme assays were optimized for less than 10% formation of the product. Reactions were initiated with DHBA and were incubated at room temperature for 6 min for SAM/TeSAM substrates, 7.5 min for FMeTeSAM, and quenched with an equal volume of 100 mM H_2SO_4. For COMT reaction using SAM, the concentration of SAM varied between 0 – 30 µM (0, 2, 4, 6, 8, 10, 20, 30 µM), and 0.398 µM COMT was used. For COMT reaction using Te SAM, the concentration of TeSAM varied between 0 – 100 µM (0, 5, 10, 15, 20, 40, 50, 80, 100 µM), and 1.5 µM COMT was used. For COMT reaction using FMeTeSAM, the concentration of FMeTeSAM varied between 0 – 320 µM (0, 10, 20, 40, 80, 160, 320 µM), and 3 µM COMT was used. Kinetic assays were run in triplicate. Quenched samples were centrifuged at 16000 × *g* for 20 min before pipetting the supernatant into LC-MS autosampler vials. Components of each quenched reaction were separated on a Zorbax extend-C18 RRHD column (2.1 × 50 mm, 1.8 µm particle size) equilibrated in 100% solvent A (40 mM ammonium acetate, pH 6) and 0% solvent B (acetonitrile). A linear gradient of solvent B from 0% to 15% was applied from 0.5 to 1 min, which was followed by a linear gradient to 60% solvent B from 1 to 3 min. Solvent B was held constant at 60% from 3 to 4 min and then decreased linearly to 0% from 4 to 4.5 min and then held constant for 1.5 min to re-equilibrate the

column before subsequent sample injections. Products were detected using electrospray ionization in negative mode (ESI^-) and quantified with a single ion monitoring (SIM) method for 3-methoxy 4-hydroxy benzoic acid/3-fluoromethoxy 4-hydroxy benzoic acid (products), and Niacin (internal standard).

Kinetic assays of PNMT and quantification of products by LC-MS/MS.

General procedure for PNMT kinetic assays: Reactions were conducted in a volume of 30 µL and contained 50 mM HEPES pH 7.5, 60 µM Norepinephrine, and 100 µM tryptophan as an internal standard with varying concentrations of SAM or SAM analog. Enzyme assays were optimized for less than 10% formation of the product. Reactions were initiated with SAM or SAM analog and incubated at room temperature for 4 minutes for SAM/TeSAM substrates and quenched with an equal volume of 100 mM H_2SO_4. For PNMT reaction using SAM, the concentration of SAM varied between 0 – 16 µM (0, 0.5, 1, 1.5, 2, 3, 4, 6, 8, 16 µM), and 80 nM PNMT was used. For PNMT reaction using TeSAM, the concentration of TeSAM varied between 0 – 20 µM (0, 0.5, 1, 2, 3, 4, 5, 8, 10, 20 µM) and 243 nM PNMT was used. Kinetic assays were run in triplicate. Quenched samples were centrifuged at 16000 × *g* for 20 min before pipetting the supernatant into LC-MS auto sampler vials. For LC-MS analysis, components of each quenched reaction were separated on a Zorbax extend-C18 RRHD column (2.1 × 50 mm, 1.8 µm particle size) equilibrated in 100% solvent A (0.1 % formic acid, pH 2.4) and 0% solvent B (acetonitrile). A linear gradient of solvent B from 0% to 15% was applied from 0 to 1 min, which was followed by a linear gradient to 50% solvent B from 1 to 2 min. Solvent B was held constant at 50% from 2 to 3 min and then decreased linearly to 0% from 3 to 3.3 min and then held constant for 1 min to re-equilibrate the column before subsequent sample injections. Products were detected using electrospray ionization in positive mode (ESI^+) and quantified with a multiple reaction monitoring (MRM) method for

epinephrine (product), and tryptophan (internal standard). Time-course analysis was performed with FMeTeSAM to monitor the co-product TeHCys since the fluoromethylated epinephrine product was hydrolyzed. Time-course reactions with FMeTeSAM contained 1 mM Norepinephrine, 500 µM FMeTeSAM, and 50 µM PNMT and the reaction was initiated with FMeTeSAM and collected time points between 0 – 110 minutes. TeHCys co-product was monitored by high-resolution LC-MS on a QExactive instrument as described above.

Kinetic assays of NNMT and quantification of products by LC-MS/MS.

General procedure for NNMT kinetic assays: Reactions were conducted in a volume of 30 µL and contained 5 mM Tris pH 8.6, 1 mM DTT, 400 µM 4-dimethylaminopyridine (4-DMAP), and 100 µM tryptophan as an internal standard with varying concentrations of SAM or SAM analog. Enzyme assays were optimized for less than 10% formation of the product. Reactions were initiated with SAM or SAM analog and incubated at room temperature for 10 minutes and quenched with an equal volume of 100 mM H_2SO_4. For NNMT reaction using SAM, the concentration of SAM varied between 0 – 160 µM (0, 2.5, 5, 7.5, 10, 20, 40, 60, 80, 160 µM), and 0.5 µM NNMT was used. For NNMT reaction using TeSAM, the concentration of TeSAM varied between 0 – 640 µM (0, 2.5, 5, 10, 20, 40, 60, 80, 160, 320, 640 µM) and 0.5 µM NNMT was used. For NNMT reaction using FMeTeSAM, the concentration of FMeTeSAM varied between 0 – 640 µM (0, 5, 10, 20, 40, 80, 160, 320, 640 µM) and 1 µM NNMT was used. Kinetic assays were run in triplicate. Quenched samples were centrifuged at 16000 × *g* for 20 min before pipetting the supernatant into LC-MS auto sampler vials. Components of each quenched reaction were separated on a Zorbax extend-C18 RRHD column (2.1 × 50 mm, 1.8 µm particle size) equilibrated in 100% solvent A (0.1 % formic acid, pH 2.4) and 0% solvent B (acetonitrile). A linear gradient of solvent B from 0% to 15% was applied from 0.5 to 1 min, which was followed by a linear

gradient to 70% solvent B from 1 to 4 min. Solvent B was held constant at 70% from 4 to 5 min and then decreased linearly to 0% from 5 to 5.5 min and then held constant for 1.5 min to re-equilibrate the column before subsequent sample injections. Products were detected using electrospray ionization in positive mode (ESI^+) and quantified with a multi reaction monitoring (MRM) method for *N*-methylated or fluoromethylated 4-dimethylamino pyridine (products), and tryptophan (internal standard).

Guanine N1-RNA methylase (TrmD) assays.

E. coli $tRNA^{pro}$ of the anti-codon stem-loop (ACSL) UGG (5'-

GGGCGAGUAGCGCAGCUUGGUAGCGCAACUGGUUUGGGACCAGUGGGUCGGAGG

UUCGAAUCCUCUCUCGCCCACCA-3') was prepared by *in vitro* transcription (IVT) from a DNA template by T7 RNA polymerase.[3] The DNA template was generated using two overlapping primers.

Forward primer (5'-3'):

CGGAAATAATACGACTCACTATAGGGCGAGTAGCGCAGCTTGGTAGCGCAACTGGT

TTG.

Reverse primer (5'- 3'):

TmGGTGGGCGAGAGAGGATTCGAACCTCCGACCCACTGGTCCCAAACCAGTTGCGC

TAC.

Enzymatic reactions were conducted in a volume of 220 µL, and the reaction contained 50 mM Tris pH 8.0, 15 µM TrmD, 180 µM $tRNA^{pro}$, 10 mM $MgCl_2$, 200 µM FMeTeSAM or SAM at 30 °C. Reactions were initiated with the addition of TrmD and 20 µL aliquots were removed at various times and quenched with an equal volume of 100 mM H_2SO_4 containing 100 µM L-tryptophan (internal standard) and the solution was neutralized with an equal volume of 2X buffer (0.25M

NH_4OAc pH 6.0, 45 mM NaCl and 4 mM $ZnCl_2$). The tRNA was digested into individual nucleosides by adding P1 nuclease (0.4 U) and Antarctic phosphatase (5 U), and the solution was incubated at 37 °C overnight. The samples were subjected to centrifugation (16000 × *g*, 30 min) to remove any precipitated material. Product analysis by high-resolution LC-MS was performed on a Q-Exactive instrument as described above.

Kinetic assays of TPMT and quantification of products by LC-MS/MS.

General procedure for TPMT kinetic assays: Reactions were conducted in a volume of 30 µL and contained 150 mM phosphate buffer pH 6.5, 3 mM 6-mercapto purine (6-MP), and 100 µM tryptophan as an internal standard with varying concentrations of SAM or SAM analog. Enzyme assays were optimized for less than 10% formation of the product. Reactions were initiated with SAM or SAM analog and incubated at room temperature for 20 minutes for SAM, 15 minutes for TeSAM and 30 minutes for FMeTeSAM and quenched with an equal volume of 100 mM H_2SO_4. For TPMT reaction using SAM, the concentration of SAM varied between 0 – 80 µM (0, 1, 2.5, 5, 10, 15, 20, 40, 80 µM), and 0.4 µM TPMT was used. For TPMT reaction using TeSAM, the concentration of TeSAM varied between 0 – 80 µM (0, 1, 2.5, 5, 10, 15, 20, 40, 80 µM), and 0.45 µM TPMT was used. For TPMT reaction using FMeTeSAM, the concentration of FMeTeSAM varied between 0 – 640 µM (0, 5, 10, 20, 40, 80, 160, 320, 640 µM), and 0.6 µM TPMT was used. Kinetic assays were run in triplicate. Quenched samples were centrifuged at 16000 × *g* for 20 min before pipetting the supernatant into LC-MS auto sampler vials. Components of each quenched reaction were separated on a Zorbax extend-C18 RRHD column (2.1 × 50 mm, 1.8 µm particle size) equilibrated in 100% solvent A (0.1 % formic acid, pH 2.4) and 0% solvent B (acetonitrile). A linear gradient of solvent B from 0% to 30% was applied from 0.5 to 1 min, which was followed by a linear gradient to 60% solvent B from 1 to 3 min. Solvent B was held

constant at 60% from 3 to 3.5 min and then decreased linearly to 0% from 3.5 to 4.5 min and then held constant for 1.5 min to re-equilibrate the column before subsequent sample injections. Products were detected using electrospray ionization in positive mode (ESI^+) and quantified with a multi reaction monitoring (MRM) method for 6-methyl or fluoromethyl mercapto purine (products), and tryptophan (internal standard).

M. SssI (DNA methylase) assays. M. SssI (CpG methyltransferase) was purchased from New England Biolabs. 26-bp DNA double-stranded substrate (CTACCTTGGTATCGATTGGTATGAGT) was purchased from Integrated DNA Technologies (Coralville, IA). Assays contain 26-bp DNA (54 μg, 15.6 μM), 1 mM SAM/TeSAM/FMeTeSAM, and the CpG MTase (M. SssI, varying amounts with a maximum of 100U) were incubated as per the procedure reported by Schramm and coworkers.[4] The *ds*DNA was digested at 37 °C overnight as per the procedure reported by Schramm and coworkers mentioned above. The nucleoside mixture dA, dT, dG, dC, and 5m-dC from each reaction aliquot was separated on a Zorbax extend-C18 RRHD column (2.1 × 50 mm, 1.8 μm particle size) equilibrated in 98% solvent A (0.1 % formic acid, pH 2.4) and 2% solvent B (acetonitrile) equipped with a mass spectrometer. A linear gradient of solvent B from 2% to 60% was applied from 0.5 min to 4 min, and then held constant at 60% B for 1 min before re-equilibrating the column for additional 2 min for subsequent sample injections. Products were detected using electrospray ionization in negative mode (ESI^+) using both the Neutral Loss (NL) and scan (MS2) modes.

SgvM assays. Enzymatic reactions were conducted in a volume of 100 μL and contained 50 mM Tris-HCl, pH 8.0, 50 mM $MgCl_2$, 20 mM KCl, 100 μM SgvM, 1 mM phenyl pyruvic acid or analog of phenyl pyruvic acid, and 200 μM SAM or TeSAM or 400 μM FMeTeSAM. Control experiments were performed without SAM or SAM analog. The activity assays were incubated at

room temperature for 18 hours and were quenched with an equal volume of acetonitrile. Precipitated enzymes were removed by centrifugation (16000 × *g*, 30 min). LC-MS analysis was performed on Zorbax extend-C18 RRHD column (2.1 × 50 mm, 1.8 μm particle size) equilibrated in 98% solvent A (0.1 % formic acid, pH 2.4) and 2% solvent B (acetonitrile). A linear gradient of solvent B from 2% to 15% was applied from 0.5 min to 1 min, followed by 15% to 30% of solvent B from 1 to 1.5 min. Solvent B was then increased linearly to 100% from 1.5 to 2 min before holding constant from 2 to 2.5 min. Solvent B was then decreased linearly to 2% from 2.5 to 3 min and then held constant for 0.5 min to re-equilibrate the column before subsequent sample injections. Products were detected using electrospray ionization in negative scan mode (ESI^-).

NovO assays. Enzymatic reactions were conducted in a volume of 50 μL and contained 100 mM Tris pH 8.0, 0.1 mg/mL BSA, 20 μM NovO, 120 μM coumarin substrate (dissolved in DMSO, <5% DMSO in volume in the reaction), 600 μM SAM/TeSAM/FMeTeSAM substrate. Reactions were initiated with the addition of enzyme. Control experiments were performed without enzyme, without SAM or SAM analog or without coumarin substrate. For derivatization of fluoromethyl coumarin product with various nucleophiles, all the reaction conditions are same as mentioned above except that 500 μM coumarin substrate, 500 μM FMeTeSAM and 2.5 mM of individual nucleophilic substrate was used. The enzyme activity assays were incubated at 37 °C overnight and were quenched with an equal volume of acetonitrile. Precipitated enzymes were removed by centrifugation (16000 × *g*, 30 min). LC-MS analysis was performed on Zorbax extend-C18 RRHD column (2.1 × 50 mm, 1.8 μm particle size) equilibrated in 98% solvent A (0.1 % formic acid, pH 2.4) and 2% solvent B (acetonitrile). A linear gradient of solvent B from 2% to 15% was applied from 0.5 min to 1 min, followed by 15% to 30% of solvent B from 1 to 1.5 min. Solvent B was then increased linearly to 100% from 1.5 to 2 min before holding constant from 2

to 2.5 min. Solvent B was then decreased linearly to 2% from 2.5 to 3 min and then held constant for 0.5 min to re-equilibrate the column before subsequent sample injections. Products were detected using electrospray ionization in negative mode (ESI^-). For high resolution LC-MS analysis, samples were injected onto a Zorbax extend C18 column (2.1 x 50 mm, 1.8 μm particle size) equilibrated with 98% solvent A (0.1% formic acid) and 2% solvent B (0.1% formic acid in acetonitrile) with a total flow of 300 μL/min. Starting at 1.5 minutes, solvent B was increased to 100% over 5.5 min and then held at 100% for 2 minutes. UPLC effluent was introduced into HESI source (ESI+ and ESI- collected) with sheath gas flow rate of 40, auxillary gas flow rate of 20, and 320 ^oC capillary. Data was collected using full scan mode (140 – 1600) at a resolution of 120,000.

OxaC assays. Enzymatic reactions were conducted in a volume of 50 μL and contained 50 mM phosphate buffer pH 7.5, 5 μM OxaC, 150 μM meleagrin (dissolved in DMSO, 2% DMSO in volume in the reaction), 400 μM SAM/FMeTeSAM substrate, 100 μM L-tryptophan (internal standard). Reactions were initiated with the addition of OxaC. Control experiments were performed without enzyme, without SAM or SAM analog and without meleagrin. The enzyme activity assays were incubated at room temperature and 30 μL aliquots were quenched at various time-points (0, 0.5, 1, 2, 4, 8, 15, 30, 60, 120 min) with an equal volume of acetonitrile. Precipitated enzymes were removed by centrifugation (16000 × *g*, 30 min). For high resolution LC-MS analysis, samples were injected onto a Zorbax extend C18 column (4.6 mm x 50 mm, 1.8 μm particle size) equilibrated with 98% solvent A (0.1% formic acid) and 2% solvent B (0.1% formic acid in acetonitrile) with a total flow of 300 μL/min. Starting at 1.5 minutes, solvent B was increased to 100% over 5.5 min and then held at 100% for 2 minutes. UPLC effluent was introduced into HESI source (ESI+ and ESI- collected) with sheath gas flow rate of 40, auxiliary gas flow rate of 20, and 320 ^oC capillary. Data was collected using full scan mode (140 – 1600) at a resolution of 120,000.

DnrK assays. Enzymatic reactions were conducted in a volume of 50 μL and contained 100 mM HEPES pH 7.5, 50 μM DnrK, 1 μM SAH nucleosidase, 1 mM DTT, 200 μM carminomycin (dissolved in DMSO, 2% DMSO in volume in the reaction), 400 μM SAM or FMeTeSAM substrate. Reactions were initiated with the addition of DnrK. The enzyme activity assays were incubated at 37 °C and 30 μL aliquots at various time-points (0, 1, 5, 15, 30, 60, 120 min) were quenched with an equal volume of 200 mM H_2SO_4 containing 100 μM L-tryptophan (internal standard). Control experiments were performed without enzyme, without SAM or SAM analog and without meleagrin. Precipitated enzymes were removed by centrifugation (16000 × *g*, 30 min). For high resolution LC-MS analysis, samples were injected onto a Zorbax extend C18 column (4.6 mm x 50 mm, 1.8 μm particle size) equilibrated with 98% solvent A (0.1% formic acid) and 2% solvent B (0.1% formic acid in acetonitrile) with a total flow of 300 μL/min. Starting at 1.5 minutes, solvent B was increased to 100% over 5.5 min and then held at 100% for 2 minutes. UPLC effluent was introduced into HESI source (ESI+ and ESI- collected) with sheath gas flow rate of 40, auxiliary gas flow rate of 20, and 320 °C capillary. Data was collected using full scan mode (140 – 1600) at a resolution of 120,000.

Synthesis of FMeTeSAM and product standards

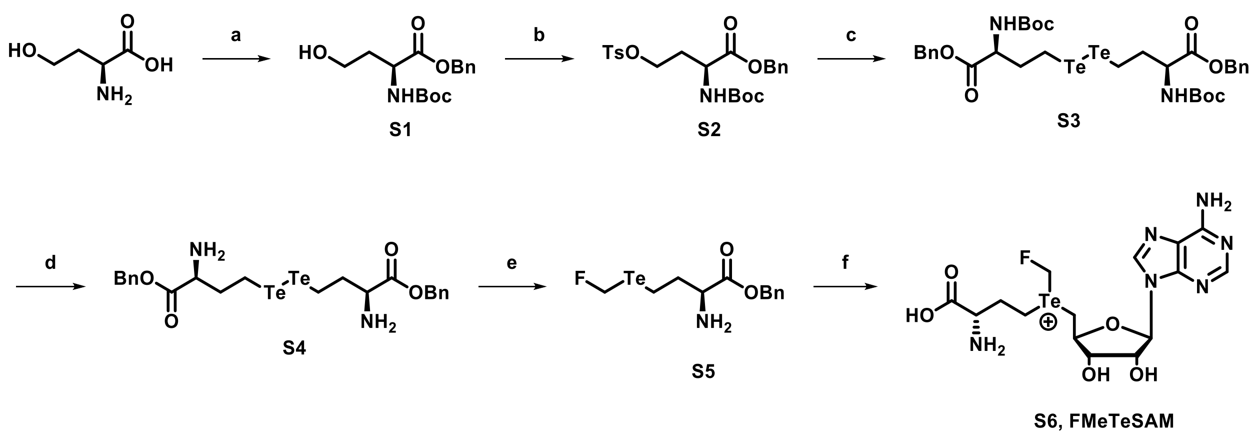

Scheme S1. Synthesis of FMeTeSAM. Reagents and conditions: a) Boc_2O, NaOH, Dioxane-water, rt, overnight; BnBr, K_2CO_3, DMF, 3h, rt, 74% over two steps b) TsCl, TEA, DCM, rt, overnight, 81%; c) Na_2Te_2, DMF, 77%; d) TFA, DCM, quant.; e) chlorofluoromethane, $NaBH_4$, DMF-EtOH, rt, 63%; e) NaOH, THF-MeOH-H_2O, then *Mj* MAT, ATP, Mg^{2+}, rt, 30 min.

Step a:

Sodium hydroxide (5.0 g, 126.0 mmol, 3.0 equiv) and Boc anhydride (18.3 g, 84.0 mmol, 2.0 equiv) were added to an ice-water cooled solution of L-homoserine (5.0 g, 42.0 mmol, 1.0 equiv) in a mixture of water (100 mL) and dioxane (100 mL), and the resulting reaction mixture was stirred at room temperature overnight. After completion, the reaction mixture was concentrated to ~100 mL by rotary evaporation, and the resulting aqueous solution was extracted with diethyl ether (100 mL) twice to remove excess Boc anhydride. The aqueous layer was collected, acidified to pH 2-3, and extracted with ethyl acetate (150 mL) twice. The organic layers were dried over anhydrous sodium sulfate and concentrated *in vacuo*, and the resulting residue was used for the next step without further purification.

K_2CO_3 (11.6 g, 84.0 mmol, 2.0 equiv) and benzyl bromide (7.5 mL, 63.0 mmol, 1.5 equiv) were added to an ice-water cooled solution of the residue in DMF (50 mL). The resulting reaction mixture was stirred at room temperature for 3 h. After completion, the reaction mixture was diluted with ethyl acetate (300 mL) and washed with water. The organic layer was concentrated in vacuo, and the resulting residue was purified by silica gel flash chromatography (hexanes : ethyl acetate = 2:1), giving 9.6 g of compound **S1** as a white solid in a yield of 74% over two steps. ^{1}H NMR (500 MHz, $CDCl^3$) δ 7.44 – 7.28 (m, 5H), 5.23 – 5.10 (m, 2H), 4.50 (s, 1H), 3.74 – 3.54 (m, 2H), 2.13 (s, 1H), 1.64 (s, 1H), 1.43 (s, 12H); ^{13}C NMR (126 MHz, $CDCl_3$) δ 172.73, 156.42, 135.24,

128.63, 128.50, 128.28, 126.97, 80.46, 67.27, 58.27, 50.77, 35.88, 28.26. HRMS: calculated for $C_{16}H_{24}NO_5^+$ [M+H$^+$]: 310.1649; found: 310.1644.

Step b:

Triethylamine (20.2 mL, 145.5 mmol, 5.0 equiv), 4-toluenesulfonyl chloride (8.3 g, 43.7 mmol, 1.5 equiv), and DMAP (356 mg, 2.9 mmol, 0.1 equiv) were added to an ice-water cooled solution of compound **S1** (9.0 g, 29.1 mmol, 1.0 equiv) in DCM (150 mL), and the resulting reaction mixture was stirred at room temperature overnight. After completion, the reaction mixture was washed thoroughly and sequentially with hydrochloric acid (0.5 M), saturated aqueous sodium bicarbonate, and brine. The organic layer was dried over anhydrous sodium sulfate and concentrated *in vacuo*, and the resulting residue was purified by silica gel flash chromatography (hexanes : ethyl acetate = 5:1), giving 11.0 g of compound **S2** as yellowish solid in a yield of 81%. ^{1}H NMR (500 MHz, $CDCl_3$) δ 7.76 (d, J = 8.2 Hz, 2H), 7.38 – 7.29 (m, 7H), 5.19 – 5.08 (m, 3H), 4.34 (s, 1H), 4.15 – 4.03 (m, 2H), 2.43 (s, 3H), 2.28 – 2.16 (m, 1H), 2.15 – 2.05 (m, 1H), 1.40 (s, 9H); ^{13}C NMR (126 MHz, $CDCl_3$) δ 171.47, 155.18, 144.96, 135.11, 132.64, 129.90, 128.65, 128.53, 128.41, 128.02, 80.16, 67.54, 66.28, 50.60, 31.45, 28.25, 21.67; **HRMS**: calculated for $C_{23}H_{30}NO_7S^+$ [M+H$^+$]: 464.1738; found: 464.1729.

Step c:

Preparation of disodium ditelluride (Na_2Te_2, 0.1 M) solution: A suspension of elemental tellurium (1.3 g, 10.0 mmol, 1.0 equiv) and sodium borohydride (1.1 g, 30.0 mmol, 3.0 equiv) in DMF (50 mL) and ethanol (50 mL) was degassed using freeze-vacuum-thaw protocol in a 500 mL Schlenk flask. The suspension was heated at 90 ^{o}C until cease of bubbling, giving a colorless solution. The resulting solution was frozen with liquid nitrogen, and another batch of elemental

tellurium (1.3 g, 10.0 mmol, 1.0 equiv) was added. After degassing, the resulting reaction mixture was heated at 90 °C for 1 hour to give a dark purple solution with Na_2Te_2 concentration at approx. 0.1 M.

The solution of compound **S2** (926 mg, 2.0 mmol, 1.0 equiv) in DMF (10 mL) and ethanol (10 mL) was degassed using freeze-vacuum-thaw protocol in a 100 mL Schlenk flask. To the resulting solution was added Na_2Te_2 solution (0.1 M, 10.0 mL, 0.5 equiv), and the resulting reaction mixture was stirred at room temperature for 30 min. After completion, the reaction mixture was diluted with ethyl acetate (150 mL) and washed thoroughly with hydrochloric acid (0.5 M), saturated aqueous sodium bicarbonate, and brine. The organic layer was dried over anhydrous sodium sulfate and concentrated *in vacuo*, and the resulting residue was purified by silica gel flash chromatography (hexanes : ethyl acetate = 3:1), giving 650 mg of compound **S3** as yellow solid in a yield of 77%. ^1H NMR (500 MHz, $CDCl_3$) δ 7.40 – 7.35 (m, 10H), 5.26 – 5.13 (m, 6H), 4.40 (s, 2H), 3.06 – 2.93 (m, 4H), 2.27 (s, 2H), 2.11 – 2.00 (m, 2H), 1.46 (s, 18H); ^{13}C NMR (126 MHz, $CDCl_3$) δ 171.87, 155.37, 135.28, 128.67, 128.53, 128.38, 80.13, 67.24, 54.93, 37.28, 28.33, -2.25; **HRMS**: calculated for $C_{32}H_{45}N_2O_8Te_2^+$ [M+H$^+$]: 845.1295; found: 845.1287.

Step d:

To the solution of compound **S3** (600 mg, 0.7 mmol, 1.0 equiv) in DCM (20 mL) was added TFA (10 mL), and the resulting red solution was stirred at room temperature for 30 min. After completion, the solvent was vanished under nitrogen flow, and the resulting residue was resuspended in DCM (100 mL). The resulting solution was washed thoroughly by saturated aqueous sodium bicarbonate. The organic layer was dried over anhydrous sodium sulfate and

concentrated *in vacuo*, and the resulting red residue was used for next step without further purification. **HRMS**: calculated for $C_{22}H_{29}N_2O_4Te_2^+$ [M+H$^+$]: 645.0246; found: 645.0239.

Step e:

The solution of compound **S4** (300 mg, 0.47 mmol, 1.0 equiv) and sodium borohydride (71 mg, 1.9 mmol, 4.0 equiv) in DMF (5 mL) and ethanol (5 mL) was degassed using freeze-vacuum-thaw protocol in a 50 mL Schlenk flask. After the color of compound **S4** faded, the reaction was cooled with ice-water bath. Then, chlorofluoromethane was bubbled into the reaction for 2 min. The resulting reaction mixture was stirred at room temperature for 30 min. After completion, the reaction mixture was diluted with ethyl acetate (50 mL) and washed thoroughly with saturated aqueous sodium bicarbonate, and brine. The organic layer was dried over anhydrous sodium sulfate and concentrated *in vacuo*, and the resulting residue was purified by silica gel flash chromatography (chloroform : methanol = 50:1), giving 210 mg of compound **S5** as yellow oil in a yield of 63%. ^{1}H NMR (500 MHz, $CDCl_3$) δ 7.38 (s, 5H), 6.27 (d, J = 51.4 Hz, 2H), 5.19 (q, J = 12.1 Hz, 2H), 3.59 (dd, J = 8.0, 4.7 Hz, 1H), 3.01 – 2.83 (m, 2H), 2.44 – 2.26 (m, 1H), 2.15 – 1.99 (m, 1H); ^{13}C NMR (126 MHz, $CDCl_3$) δ 175.31, 135.58, 128.68, 128.50, 128.36, 66.90, 64.38, 62.60 (d, $J_{C\text{-}F}$ = 222.5 Hz), 55.96, 36.83, 1.16; ^{19}F NMR (471 MHz, $CDCl_3$) δ -208.28, -208.39, -208.50; **HRMS**: calculated for $C_{12}H_{17}FNO_2Te^+$ [M+H$^+$]: 356.0300; found: 356.0291.

Step f:

To a solution of compound **S5** (8.8 mg, 25 μmol, 1.0 equiv) in methanol (350 uL) was added KOH (0.57 M, 90 uL, 2 equiv) and MeOH (80 uL), and the resulting reaction mixture was incubated at room temperature for 3 min. After completion, the reaction mixture was added to MAT reaction directly.

The MAT reaction was performed in a 5 mL scale with Tris HCl (100 mM, pH 8.0), KCl (50 mM), $MgCl_2$ (40 mM), ATP (15 mM), and Mj MAT (0.15 mM) at 37 ^oC. After 0.5 h, the reaction was quenched with MeOH (5 mL). The precipitant was palleted, and the supernatant was lyophilized. The resulting residue was dissolved in 10 mM TEAA buffer (10 mM triethylamine in water, adjust the pH to 5.0 with acetic acid) and purified by HPLC twice using Zorbax SB-C18 9.4mm X 25cm on Agilent 1260 Infinity II Prep HPLC system. The first HPLC purification was performed using solvent A (10 mM TEAA), solvent B (acetonitrile), and solvent C (methanol) as follows:

Table S1. 1st HPLC condition for the purification of FMeTeSAM

Time (min)	%A	%B	%C	Flow (mL/min)
**0**	95.0	0	5.0	4.000
**17**	20.0	50.0	30.0	4.000
**19**	0	50.0	50.0	4.000
**22**	95.0	0	5.0	4.000

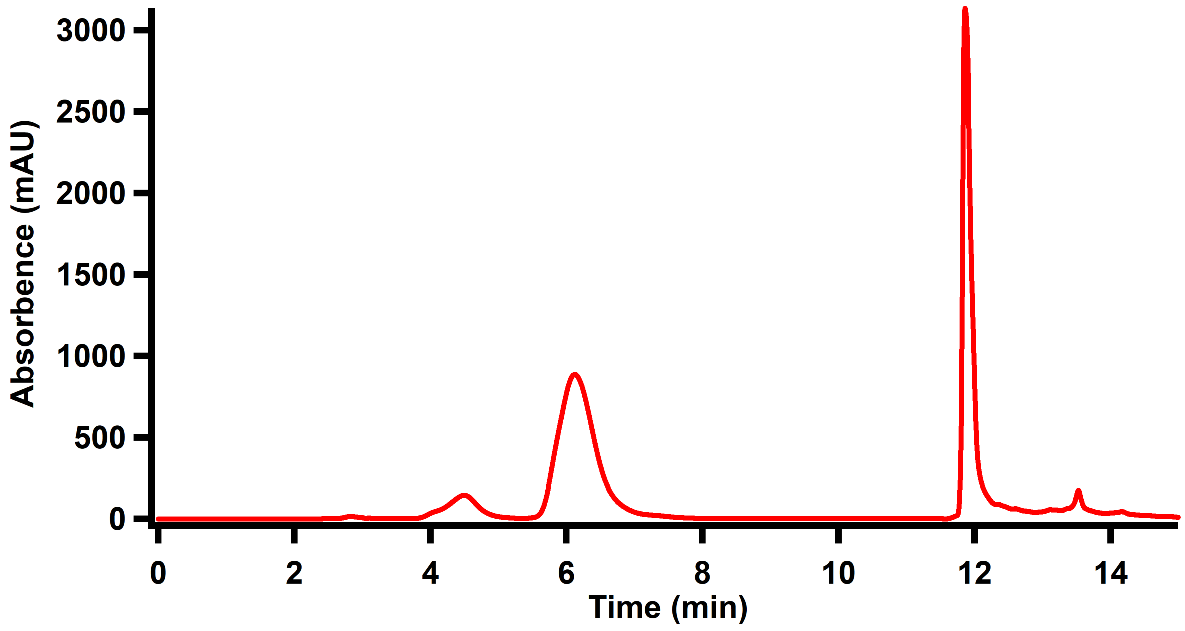

Figure S16. HPLC trace of FMeTeSAM, 1st purification.

The peak at 6.1 min contains FMeTeSAM. This peak was collected, pooled, and lyophilized. The resulting residue was dissolved in water and purified for the second time. The second HPLC purification was performed using solvent A (0.1% formic acid in water) and solvent B (acetonitrile) as follows:

Table S2. 2nd HPLC condition for the purification of FMeTeSAM

**Time (min)**	**%A**	**%B**	**Flow (mL/min)**
**0**	100	0	4.000
**4**	100	0	4.000

**20**	0	40	4.000

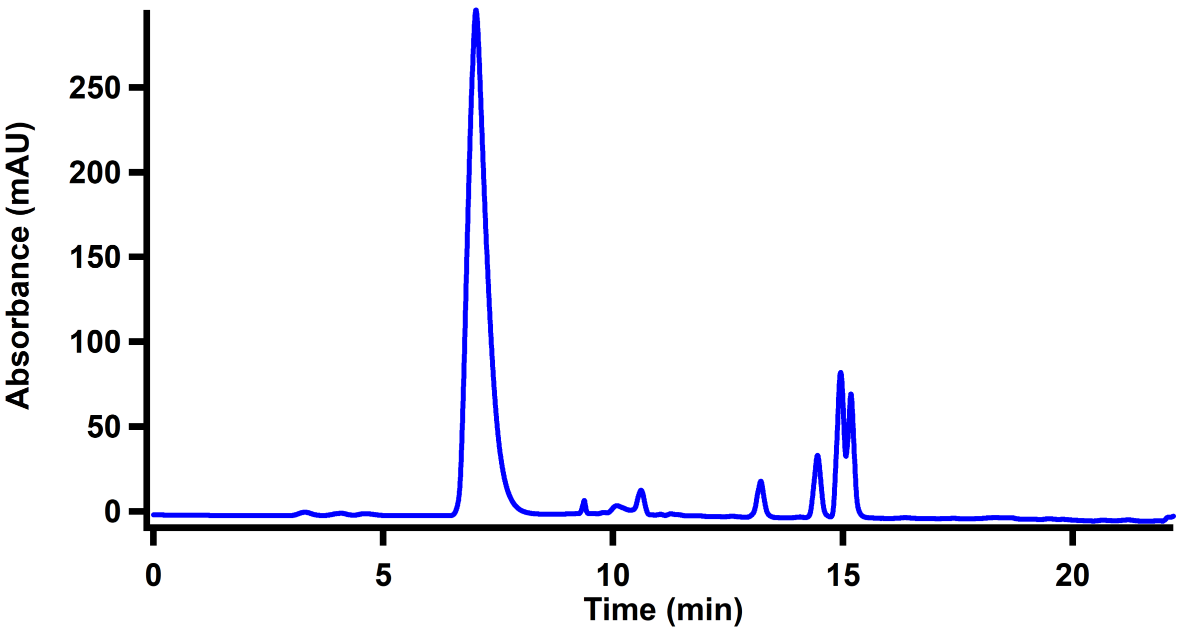

Figure S17. HPLC trace of FMeTeSAM, 2nd purification.

The peak at 7 min contains FMeTeSAM. This peak was collected, pooled, and lyophilized. The resulting residue was dissolved in water and kept under -80 ^{o}C for further use. The concentration of FMeTeSAM was calculated using extinction coefficient 15400 $M^{-1}cm^{-1}$ at 260 nM.

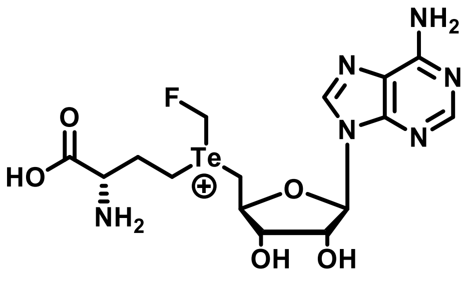

S6, FMeTeSAM

^{1}H NMR (500 MHz, D_2O) δ 8.30 (s, 1H), 8.22 (s, 1H), 8.19 (s, 1H), 5.97 (d, *J* = 4.0 Hz, 1H), 5.80 (dd, *J* = 17.8, 7.0 Hz, 1H), 5.70 (dd, *J* = 17.9, 7.0 Hz, 1H), 4.80 – 4.77 (m, 1H), 4.49 – 4.40 (m, 2H), 3.67 (dd, *J* = 9.4, 3.9 Hz, 1H), 3.55 (dd, *J* = 12.3, 4.0 Hz, 1H), 3.28 (dd, *J* = 12.3, 7.6 Hz, 1H), 3.17 (t, *J* = 6.6 Hz, 2H), 2.39 – 2.22 (m, 2H); ^{13}C NMR (126 MHz, D_2O) δ 173.39, 169.81, 154.92, 151.77, 148.58, 141.03, 119.16, 89.06, 79.50, 78.83, 77.01 (d, $J_{C\text{-}F}$ = 227.5 Hz), 74.04, 73.09, 53.57, 46.61, 28.42, 28.39, 26.57, 21.83, 21.81, 8.16; ^{19}F NMR (471 MHz, D_2O) δ -229.03, -229.12, -229.22; **HRMS**: calculated for $C_{15}H_{22}FN_6O_5Te^+$ [M^+]: 515.0693; found: 515.0679.

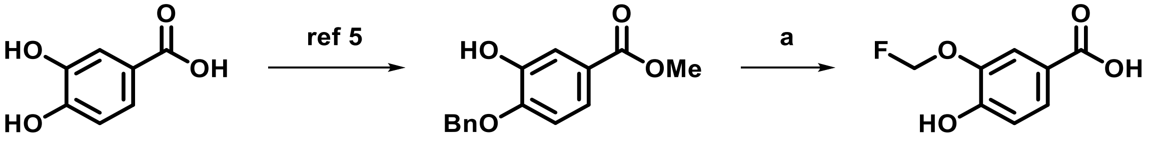

S7 **S8**

Scheme S2. Synthesis of 3-(fluoromethoxy)-4-hydroxybenzoic acid.

Compound **S7** was synthesized as a methyl ester according to the reported procedure[5].

^{1}H NMR (500 MHz, $CDCl_3$) δ 7.63 – 7.56 (m, 2H), 7.45 – 7.34 (m, 5H), 6.94 (d, *J* = 8.4 Hz, 1H), 5.16 (s, 2H), 3.88 (s, 3H); ^{13}C NMR (126 MHz, $CDCl_3$) δ 166.79, 149.58, 145.46, 135.57, 128.85, 128.69, 127.91, 123.68, 122.71, 115.85, 111.21, 71.13, 52.02.

Step a:

To a stirred solution of compound **S7** (258.3 mg, 1.0 mmol, 1.0 equiv) and Cs_2CO_3 (650.0 mg, 2.0 mmol, 2.0 equiv) in DMF (10 mL) was bubbled fluorochloromethane for 5 min. The resulting reaction mixture was stirred at room temperature for 1 hour. After completion, the reaction mixture was diluted with ethyl acetate (50 mL) and washed thoroughly with water and brine. The organic layer was dried over anhydrous sodium sulfate and concentrated *in vacuo*, and the resulting residue was used for next step without further purification.

The residue and Pd/C (50 mg, 10%) were suspended in MeOH (10 mL), and catalytic hydrogenation was performed under H_2 atmosphere (1 atm) for 2 hours. Pd/C was filtered and the filtrate was concentrated *in vacuo.* The resulting residue was used for next step without further purification.

To a stirred solution of the residue in THF (5 mL), MeOH (5 mL), and water (5 mL) was added NaOH (200 mg, 5.0 mmol, 5 equiv). The resulting reaction mixture was stirred at room temperature for 2 hours. After completion, the reaction mixture was acidified to pH 2.0 using HCl (1M) and extracted with EtOAc (50 mL). The organic layer was dried over anhydrous sodium sulfate and concentrated *in vacuo*, and the resulting residue was purified by silica gel flash chromatography (chloroform : methanol = 50:1), giving 120 mg of compound **S8** as white solid in a yield of 65%. ^{1}H NMR (500 MHz, DMSO) δ 12.63 (s, 1H), 10.34 (s, 1H), 7.67 – 7.61 (m, 1H), 7.61 – 7.56 (m, 1H), 6.95 (d, *J* = 8.3 Hz, 1H), 5.81 (d, *J* = 54.6 Hz, 2H); ^{13}C NMR (126 MHz, DMSO) δ 167.24, 152.47, 144.20, 126.85, 122.22, 119.06, 116.76, 102.95, 101.24 (d, $J_{C\text{-}F}$ = 215.5 Hz); ^{19}F NMR (471 MHz, DMSO) δ -149.10, -149.21, -149.33; **HRMS**: calculated for $C_8H_6FO_4^-$ [M-H$^+$]: 185.0256; found: 185.0254.

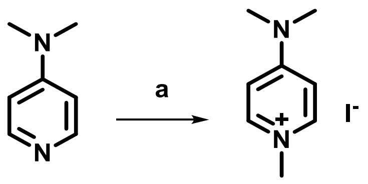

S9

Scheme S3. Synthesis of 4-(dimethylamino)-1-methylpyridin-1-ium iodide.

Compound **S9** is synthetized according to the reported procedure.[6] ^{1}H NMR (500 MHz, DMSO) δ 8.24 (d, *J* = 7.6 Hz, 2H), 7.03 (d, *J* = 7.7 Hz, 2H), 3.92 (s, 3H), 3.18 (s, 6H); ^{13}C NMR (126 MHz, DMSO) δ 156.10, 143.29, 107.96, 44.50.

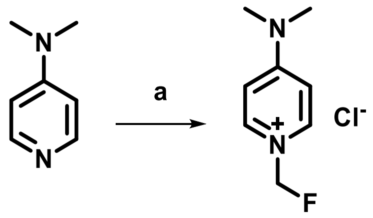

S10

Scheme S4. Synthesis of 4-(dimethylamino)-1-(fluoromethyl)pyridin-1-ium iodide.

Fluorochloromethane gas was solidified into a 100 mL pressure vessel by liquid nitrogen. DMAP (122 mg, 1.0 mmol, 1 equiv) in acetonitrile (20 mL) was added to the pressure vessel. The reaction mixture was sealed and heated at 100 °C overnight. After completion, the product crystallized from the reaction and was collected by filtration. ^{1}H NMR (500 MHz, DMSO) δ 8.55 (d, *J* = 7.9 Hz, 2H), 7.17 (d, *J* = 7.8 Hz, 2H), 6.23 (d, *J* = 50.3 Hz, 2H), 3.27 (s, 6H); ^{13}C NMR (126 MHz, DMSO) δ 157.38, 142.76, 108.29, 93.58 ($J_{C\text{-}F}$ = 203 Hz); ^{19}F NMR (471 MHz, DMSO) δ -164.55, -164.66, -164.77; **HRMS**: calculated for $C_8H_{12}FN_2^+$ [M+H$^+$]: 155.0979; found: 155.0974.

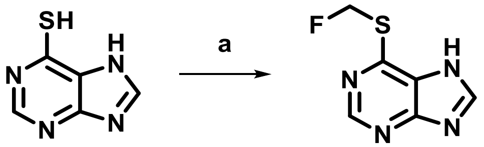

S11

Scheme S5. Synthesis of 6-((fluoromethyl)thio)-7*H*-purine.

To a stirred solution of 6-mercaptopurine (152 mg, 1.0 mmol, 1.0 equiv) and Cs_2CO_3 (650 mg, 1.0 equiv, 2.0 equiv) in DMF (10 mL) was bubbled fluorochloromethane for 5 min. The reaction mixture was stirred at 55 °C for 1h. After completion, the reaction mixture was diluted with EtOAc (50 mL) and washed with water. The organic layer was dried over anhydrous sodium sulfate and concentrated *in vacuo*, and the resulting residue was purified by silica gel flash chromatography (chloroform : methanol = 50:1), giving 50 mg of compound **S11** as yellowish solid in a yield of 27%. ^{1}H NMR (500 MHz, DMSO) δ 13.70 (s, 1H), 8.80 (s, 1H), 8.55 (s, 1H), 6.45 (d, J = 50.6 Hz, 2H); ^{13}C NMR (126 MHz, DMSO) δ 154.89, 150.77, 149.16, 144.58, 130.97, 82.11, 80.41; ^{19}F NMR (471 MHz, DMSO) δ -188.84, -188.94, -189.05; **HRMS**: calculated for $C_6H_6FN_4S^+$ [M+H$^+$]: 185.0292; found: 185.0288.

References

1. Iwig, D. F.; Booker, S. J., Insight into the polar reactivity of the onium chalcogen analogues of S-adenosyl-L-methionine. *Biochemistry-Us* **2004,** *43* (42), 13496-13509.
2. Newmister, S. A.; Romminger, S.; Schmidt, J. J.; Williams, R. M.; Smith, J. L.; Berlinck, R. G. S.; Sherman, D. H., Unveiling sequential late-stage methyltransferase reactions in the meleagrin/oxaline biosynthetic pathway. *Organic & Biomolecular Chemistry* **2018,** *16* (35), 6450-6459.
3. Milligan, J. F.; Uhlenbeck, O. C., Synthesis of Small Rnas Using T7 Rna-Polymerase. *Methods in Enzymology* **1989,** *180*, 51-62.
4. Du, Q.; Wang, Z.; Schramm, V. L., Human DNMT1 transition state structure. *Proc Natl Acad Sci U S A* **2016,** *113* (11), 2916-21.
5. Zhang, Q. Z.; Raheem, K. S.; Botting, N. P.; Slawin, A. M. Z.; Kay, C. D.; O'Hagan, D., Flavonoid metabolism: the synthesis of phenolic glucuronides and sulfates as candidate metabolites for bioactivity studies of dietary flavonoids. *Tetrahedron* **2012,** *68* (22), 4194-4201.
6. Rollema, H.; Johnson, E. A.; Booth, R. G.; Caldera, P.; Lampen, P.; Youngster, S. K.; Trevor, A. J.; Naiman, N.; Castagnoli, N., Jr., In vivo intracerebral microdialysis studies in rats of MPP+ analogues and related charged species. *J Med Chem* **1990,** *33* (8), 2221-30.

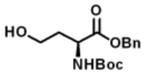

S1

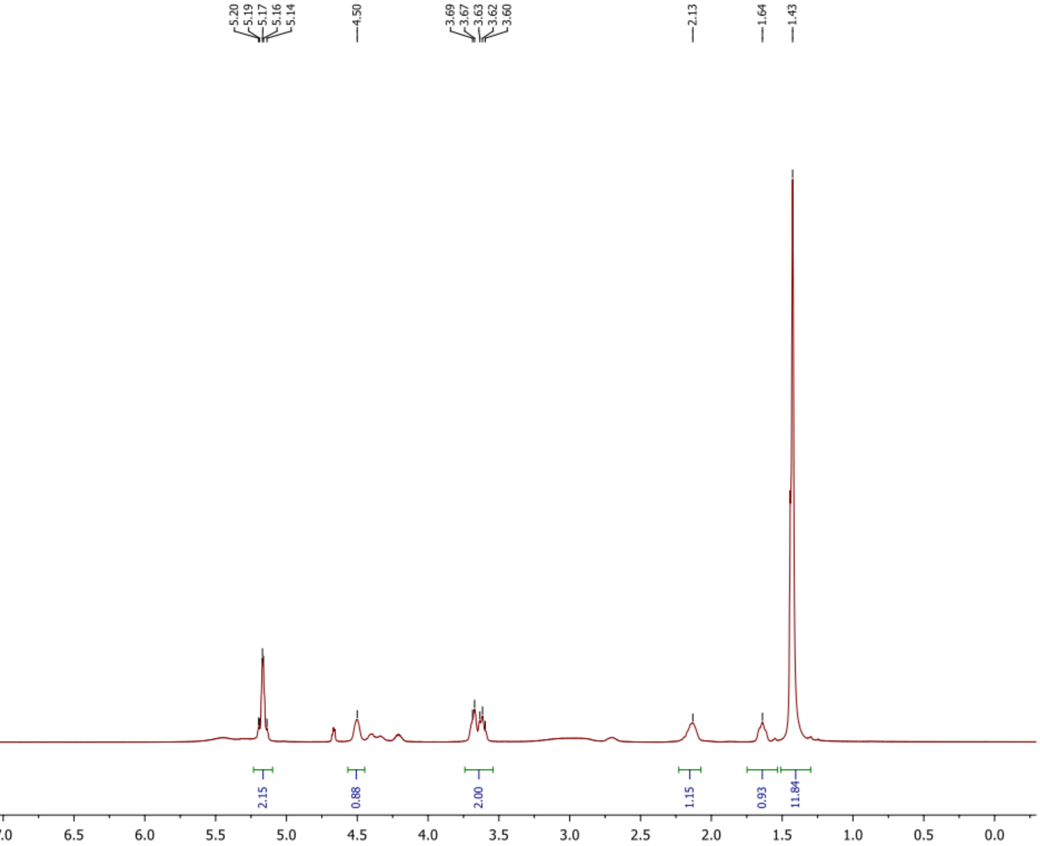

7.34
7.34
5.20
5.19
5.17
5.16
5.14
4.50
3.69
3.67
3.63
3.62
3.60
2.13
1.64
1.43
5.45
2.15
0.88
2.00
1.15
0.93
11.84
8.0
7.5
7.0
6.5
6.0
5.5
5.0
4.5
4.0
3.5
3.0
2.5
2.0
1.5
1.0
0.5
0.0

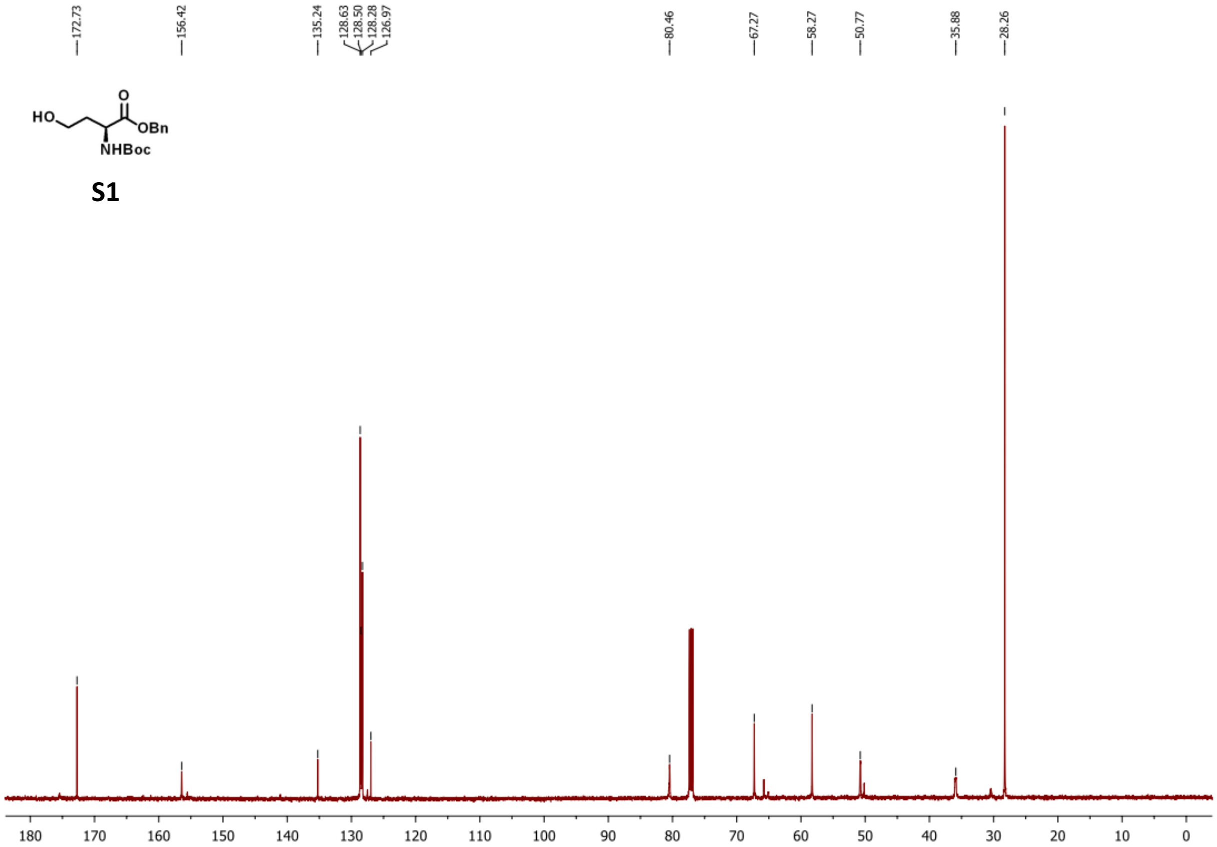
172.73
156.42
135.24
128.63
128.50
128.28
126.97
80.46
67.27
58.27
50.77
35.88
28.26
HO
O
OBn
NHBoc
180 170 160 150 140 130 120 110 100 90 80 70 60 50 40 30 20 10 0

S1

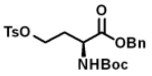
TsO
O
OBn
NHBoc

S2

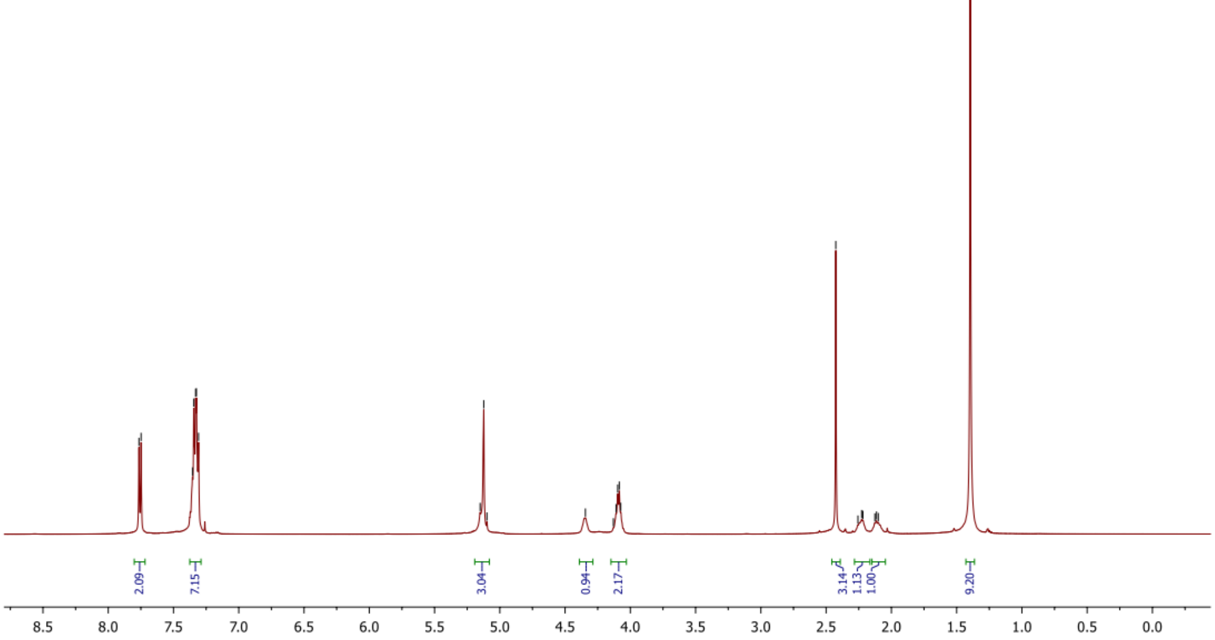
7.76
7.75
7.35
7.34
7.33
7.32
7.31
5.15
5.12
5.10
4.34
4.13
4.11
4.10
4.08
4.07
2.43
2.26
2.23
2.22
2.13
2.11
2.10
1.40
2.09
7.15
3.04
0.94
2.17
3.14
1.13
1.00
9.20
8.5
8.0
7.5
7.0
6.5
6.0
5.5
5.0
4.5
4.0
3.5
3.0
2.5
2.0
1.5
1.0
0.5
0.0

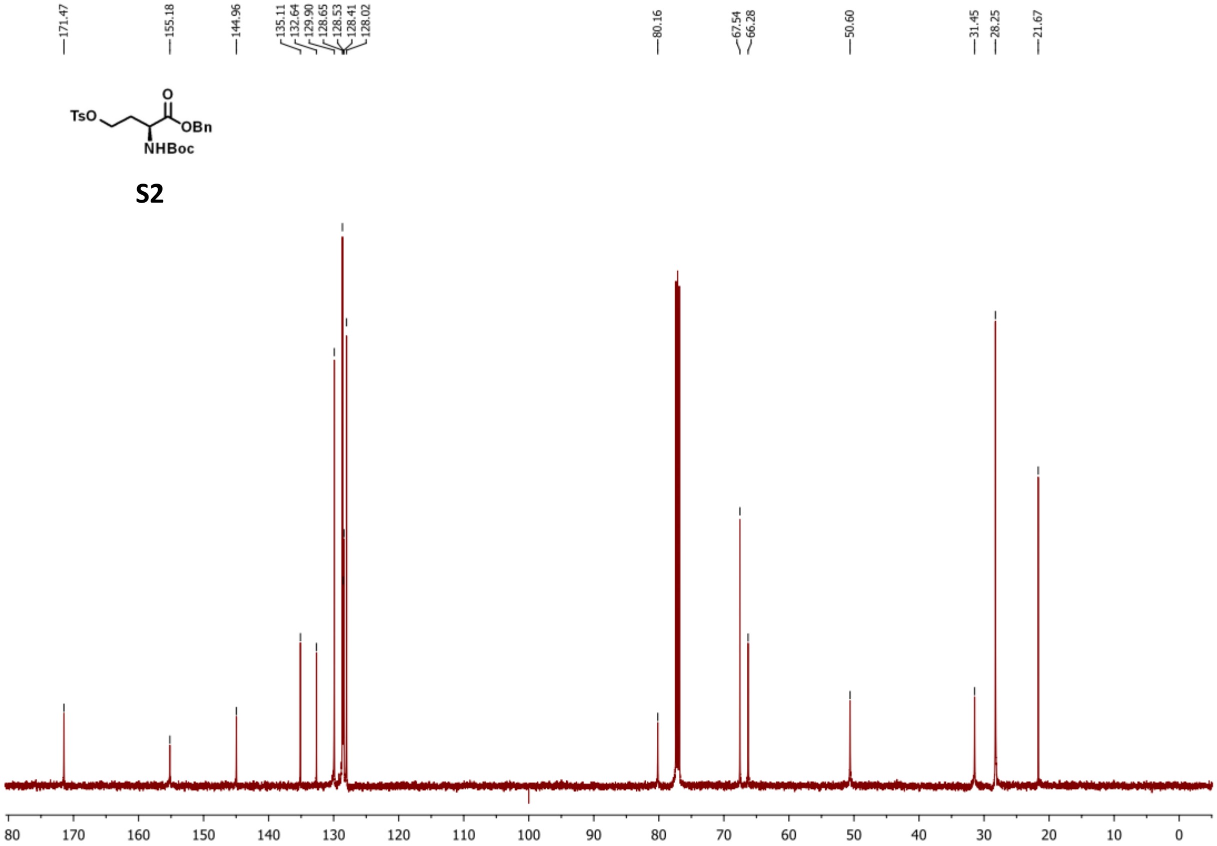
171.47
155.18
144.96
135.11
132.64
129.90
128.65
128.53
128.41
128.02
80.16
67.54
66.28
50.60
31.45
28.25
21.67
TsO
O
OBn
NHBoc
S2
180 170 160 150 140 130 120 110 100 90 80 70 60 50 40 30 20 10 0

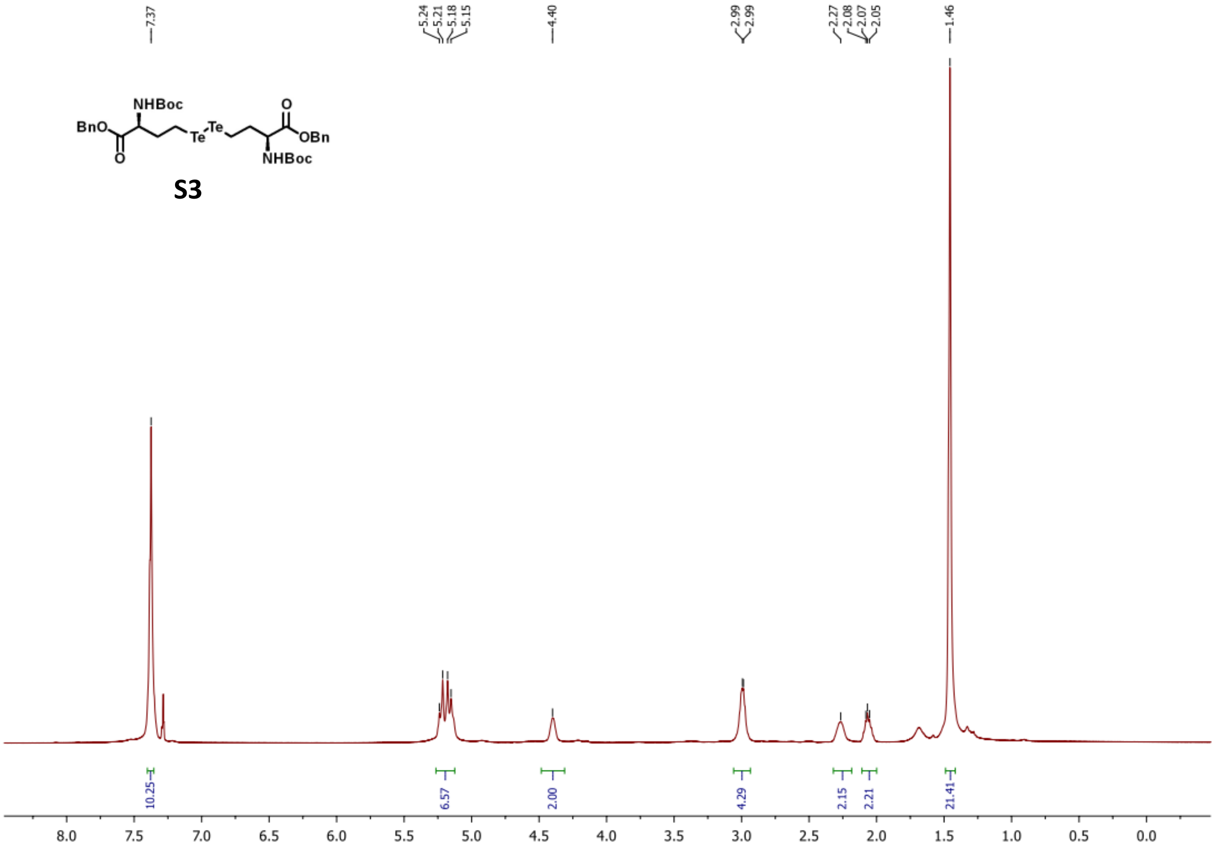
NHBoc
BnO
Te
Te
O
OBn
O
NHBoc
7.37
5.24
5.21
5.18
5.15
4.40
2.99
2.99
2.27
2.08
2.07
2.05
1.46
10.25
6.57
2.00
4.29
2.15
2.21
21.41
8.0
7.5
7.0
6.5
6.0
5.5
5.0
4.5
4.0
3.5
3.0
2.5
2.0
1.5
1.0
0.5
0.0

S3

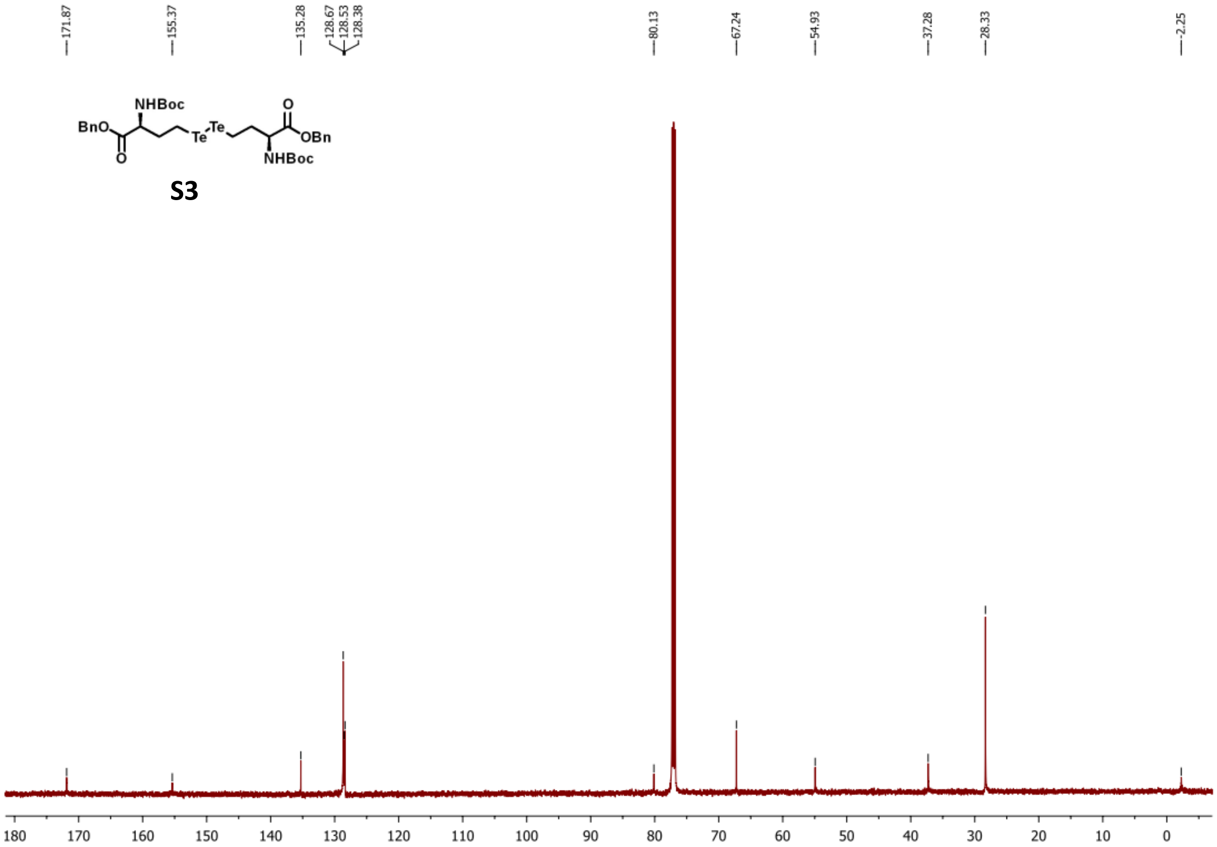

171.87
155.37
135.28
128.67
128.53
128.38
80.13
67.24
54.93
37.28
28.33
2.25
NHBoc
BnO
O
Te
Te
O
OBn
NHBoc
180 170 160 150 140 130 120 110 100 90 80 70 60 50 40 30 20 10 0

S3

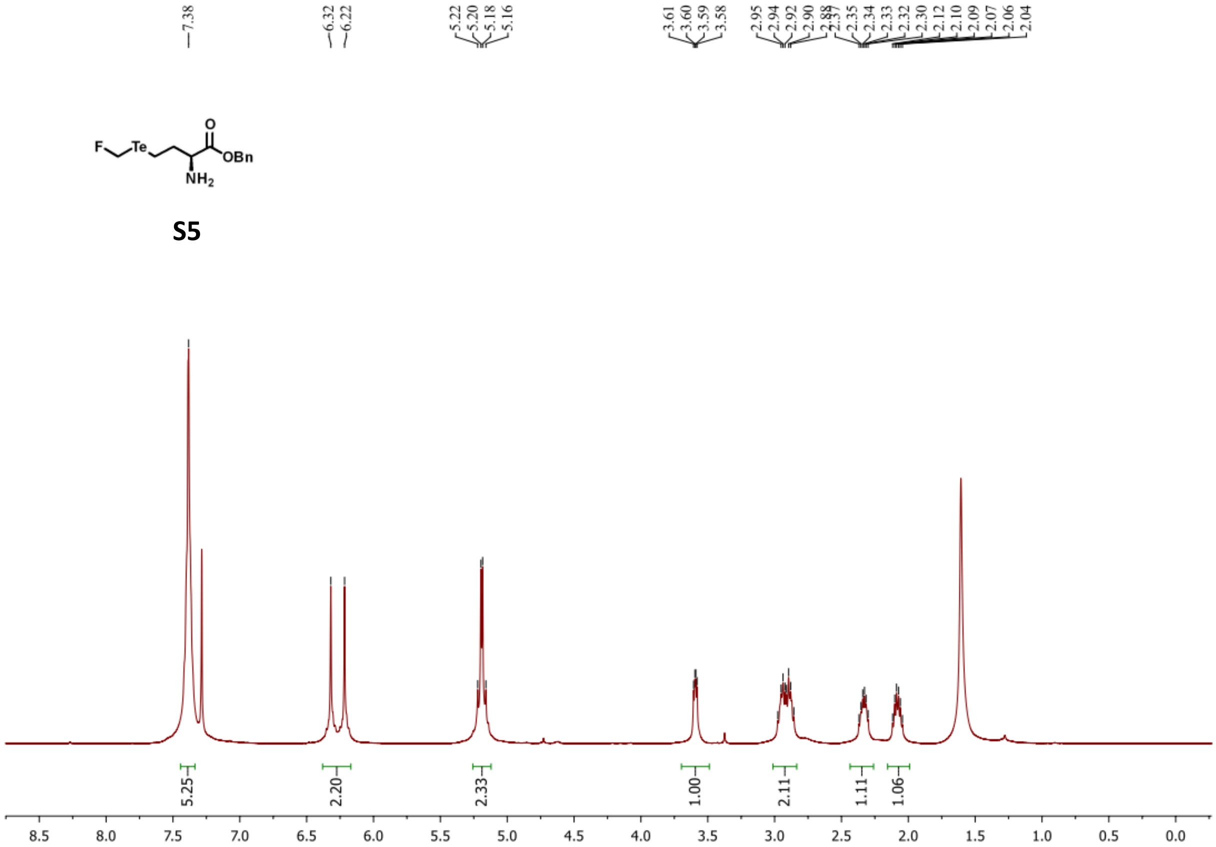
7.38
6.32
6.22
5.22
5.20
5.18
5.16
3.61
3.60
3.59
3.58
2.95
2.94
2.92
2.90
2.88
2.37
2.35
2.34
2.33
2.32
2.30
2.12
2.10
2.09
2.07
2.06
2.04
F
Te
O
OBn
NH_2
5.25
2.20
2.33
1.00
2.11
1.11
1.06
8.5
8.0
7.5
7.0
6.5
6.0
5.5
5.0
4.5
4.0
3.5
3.0
2.5
2.0
1.5
1.0
0.5
0.0

S5

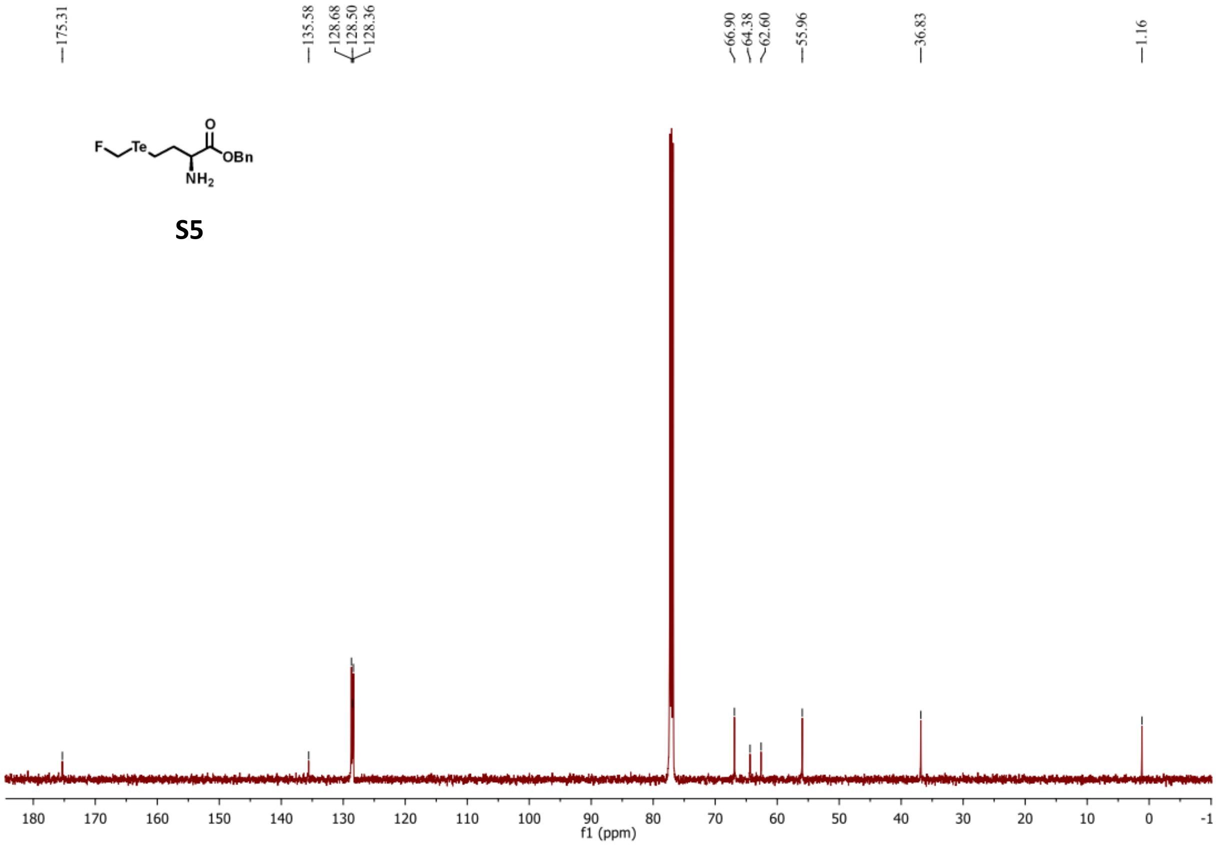
175.31
135.58
128.68
128.50
128.36
66.90
64.38
62.60
55.96
36.83
1.16
F
Te
O
OBn
NH2
S5
180
170
160
150
140
130
120
110
100
90
80
70
60
50
40
30
20
10
0
-1
f1 (ppm)

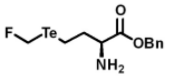

S5

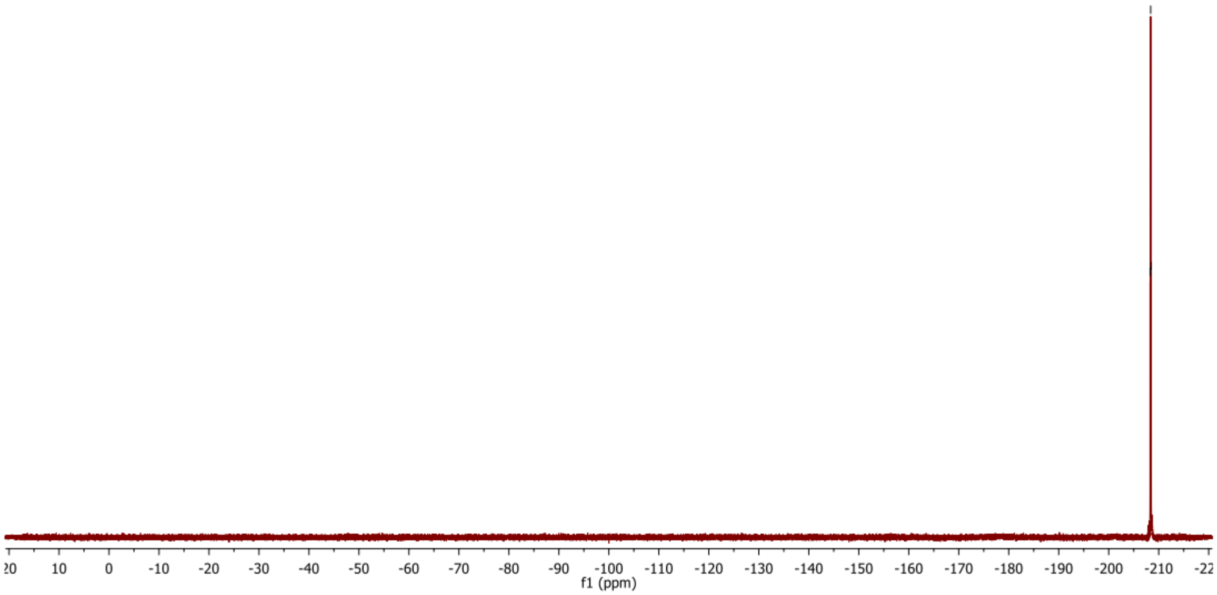

-208.28
-208.39
-208.50
20
10
0
-10
-20
-30
-40
-50
-60
-70
-80
-90
-100
-110
-120
-130
-140
-150
-160
-170
-180
-190
-200
-210
-22
f1 (ppm)

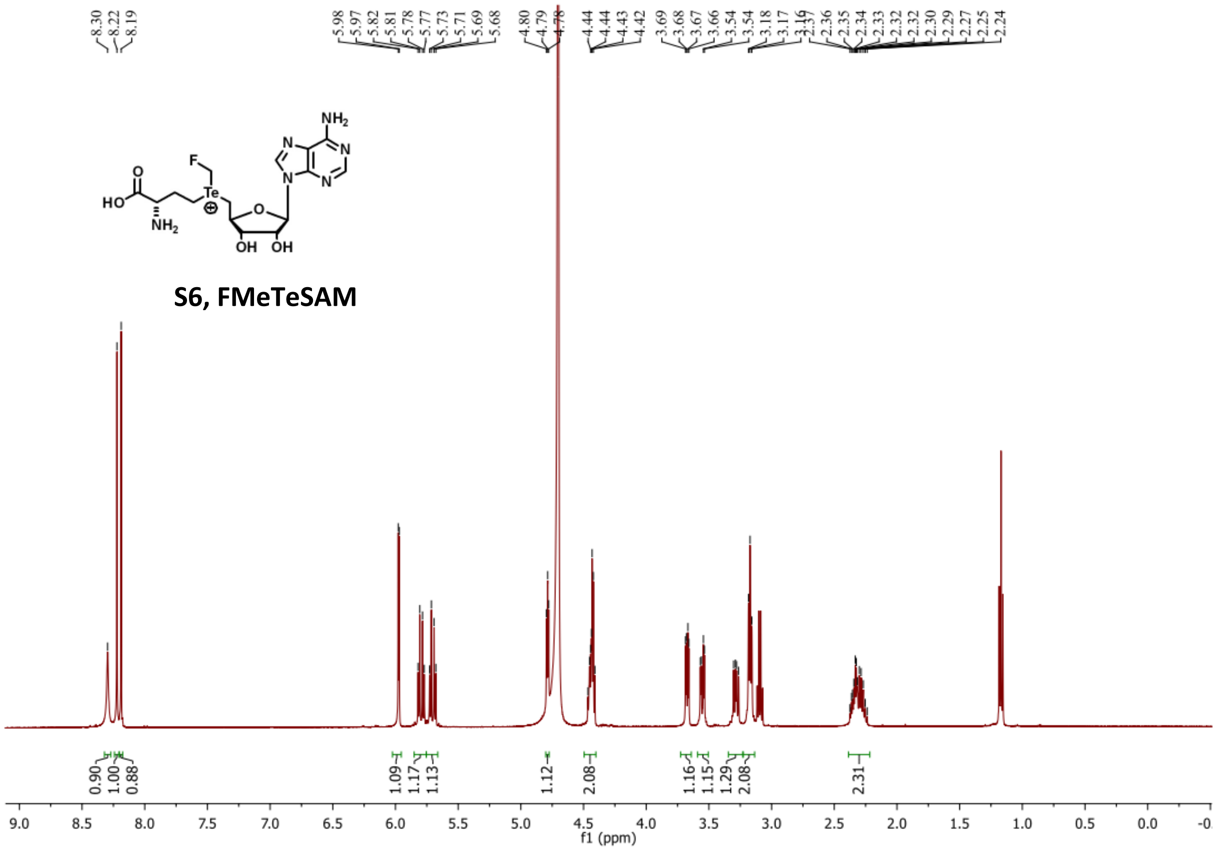

8.30
8.22
8.19
5.98
5.97
5.82
5.81
5.78
5.77
5.73
5.71
5.69
5.68
4.80
4.79
4.78
4.44
4.44
4.43
4.42
3.69
3.68
3.67
3.66
3.54
3.54
3.18
3.17
3.16
2.37
2.36
2.35
2.34
2.33
2.32
2.32
2.30
2.29
2.27
2.25
2.24
NH2
N
N
N
N
F
O
HO
Te
O
NH2
OH
OH
0.90
1.00
0.88
1.09
1.17
1.13
1.12
2.08
1.16
1.15
1.29
2.08
2.31
9.0
8.5
8.0
7.5
7.0
6.5
6.0
5.5
5.0
4.5
4.0
3.5
3.0
2.5
2.0
1.5
1.0
0.5
0.0
-0
f1 (ppm)

S6, FMeTeSAM

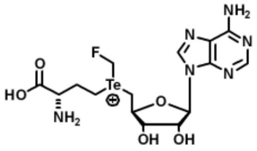
NH2
N
N
N
N
F
O
HO
Te
O
NH2
OH
OH

S6, FMeTeSAM

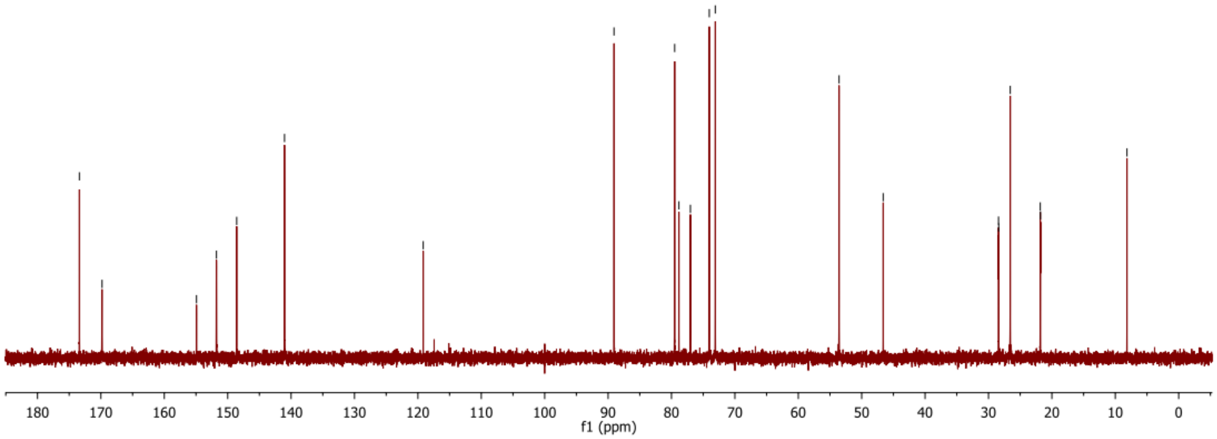
173.39
169.81
154.92
151.77
148.58
141.03
119.16
89.06
79.50
78.83
77.01
74.04
73.09
53.57
46.61
28.42
28.39
26.57
21.83
21.81
8.16
180 170 160 150 140 130 120 110 100 90 80 70 60 50 40 30 20 10 0
f1 (ppm)

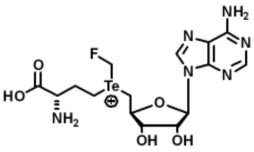

S6, FMeTeSAM

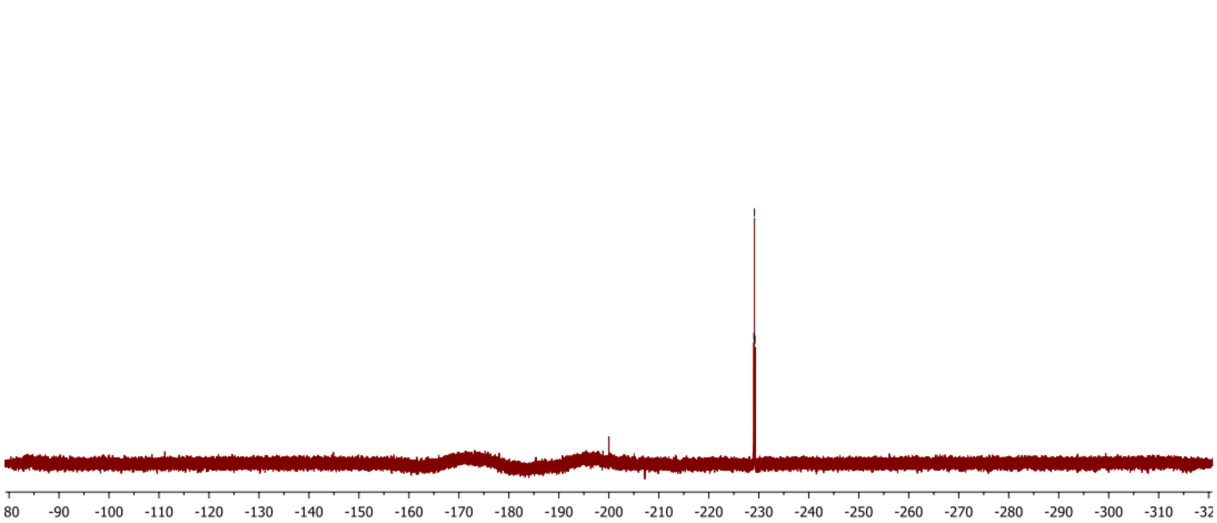

-229.03
-229.12
-229.22
80 -90 -100 -110 -120 -130 -140 -150 -160 -170 -180 -190 -200 -210 -220 -230 -240 -250 -260 -270 -280 -290 -300 -310 -32

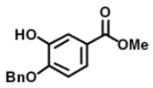

S7

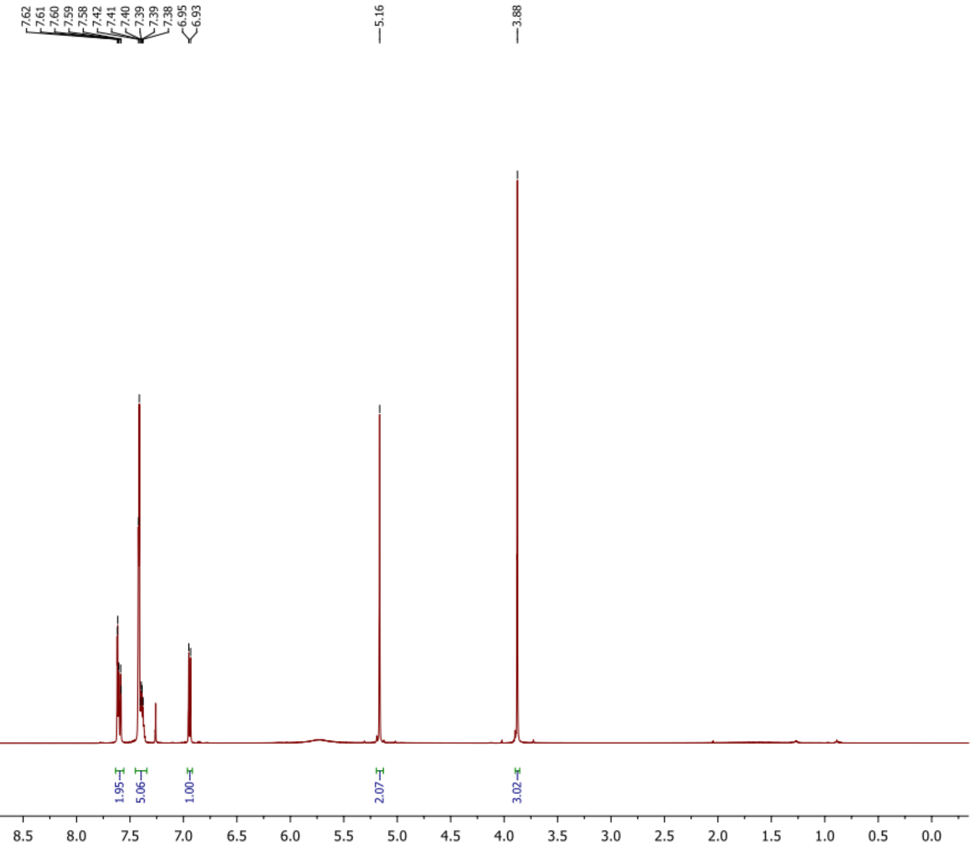
7.62
7.61
7.60
7.59
7.58
7.42
7.41
7.40
7.39
7.39
7.38
6.95
6.93
5.16
3.88
1.95
5.06
1.00
2.07
3.02
10.5
10.0
9.5
9.0
8.5
8.0
7.5
7.0
6.5
6.0
5.5
5.0
4.5
4.0
3.5
3.0
2.5
2.0
1.5
1.0
0.5
0.0

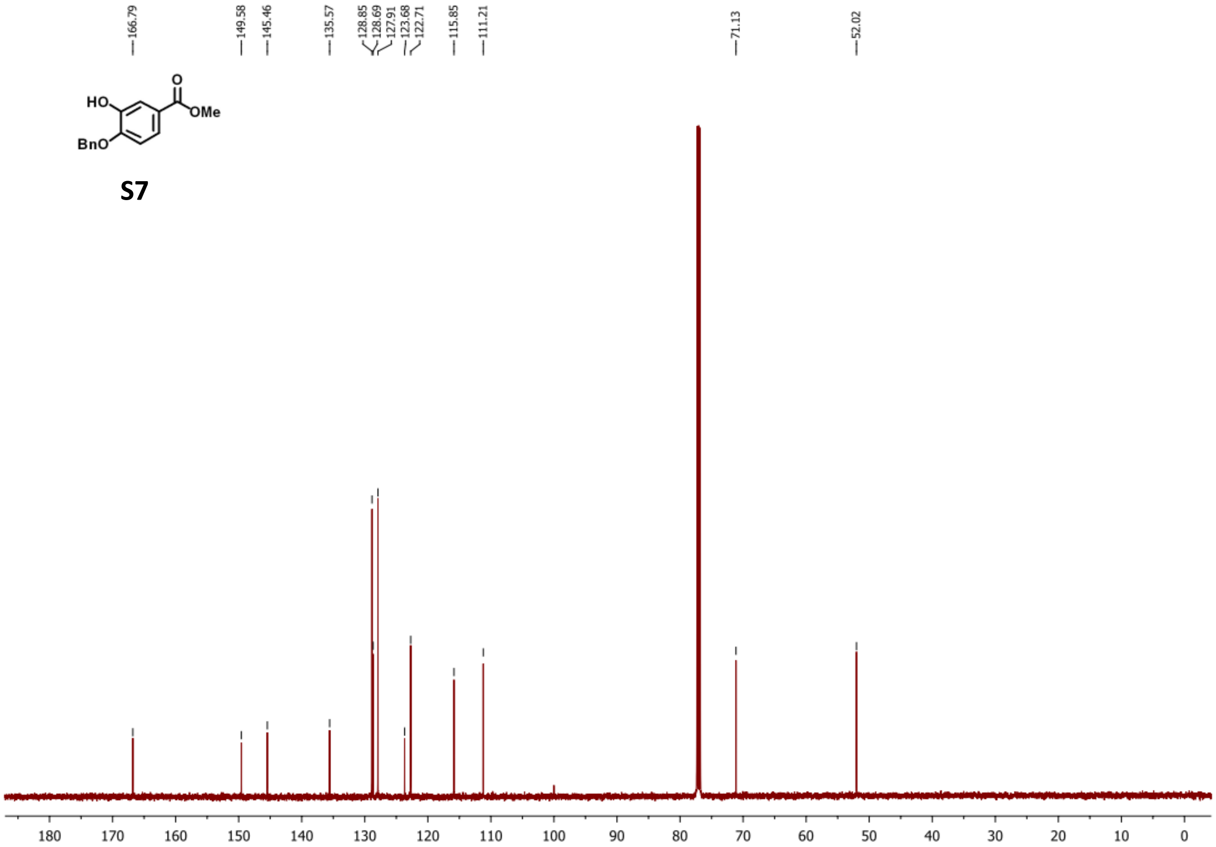
166.79
149.58
145.46
135.57
128.85
128.69
127.91
123.68
122.71
115.85
111.21
71.13
52.02
180
170
160
150
140
130
120
110
100
90
80
70
60
50
40
30
20
10
0

S7

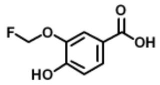

F
O
O
OH
HO

S8

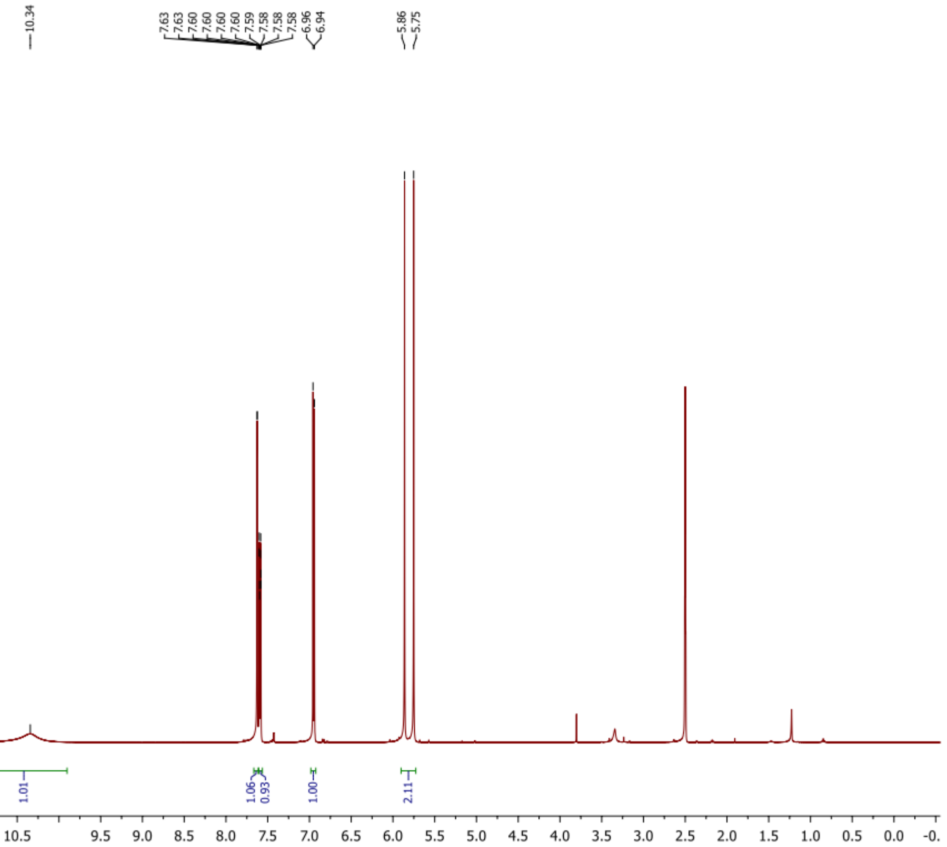

12.63
10.34
7.63
7.63
7.60
7.60
7.60
7.59
7.58
7.58
7.58
6.96
6.94
5.86
5.75
1.01
1.01
1.06
0.93
1.00
2.11
13.5 12.5 11.5 10.5 9.5 9.0 8.5 8.0 7.5 7.0 6.5 6.0 5.5 5.0 4.5 4.0 3.5 3.0 2.5 2.0 1.5 1.0 0.5 0.0 -0.

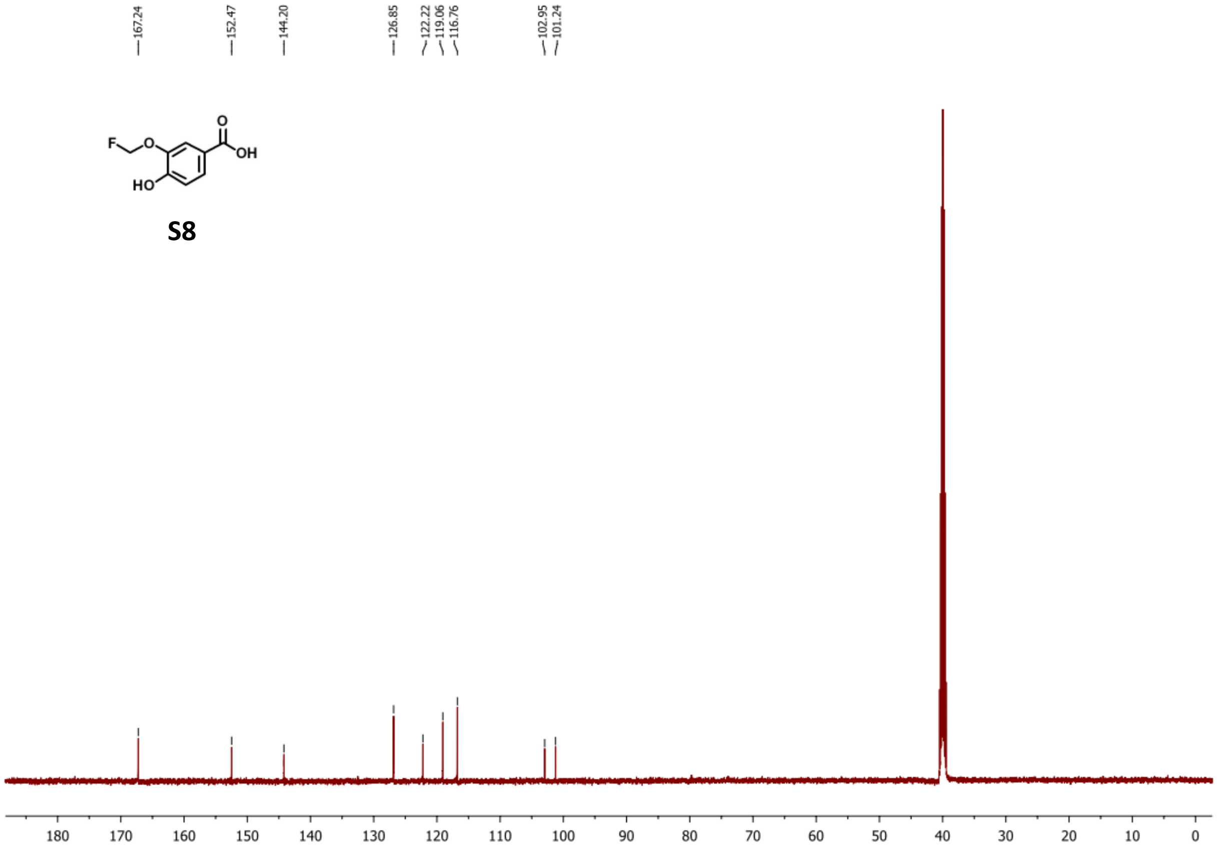
167.24
152.47
144.20
126.85
122.22
119.06
116.76
102.95
101.24
F
O
O
OH
HO
180 170 160 150 140 130 120 110 100 90 80 70 60 50 40 30 20 10 0

S8

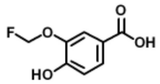

S8

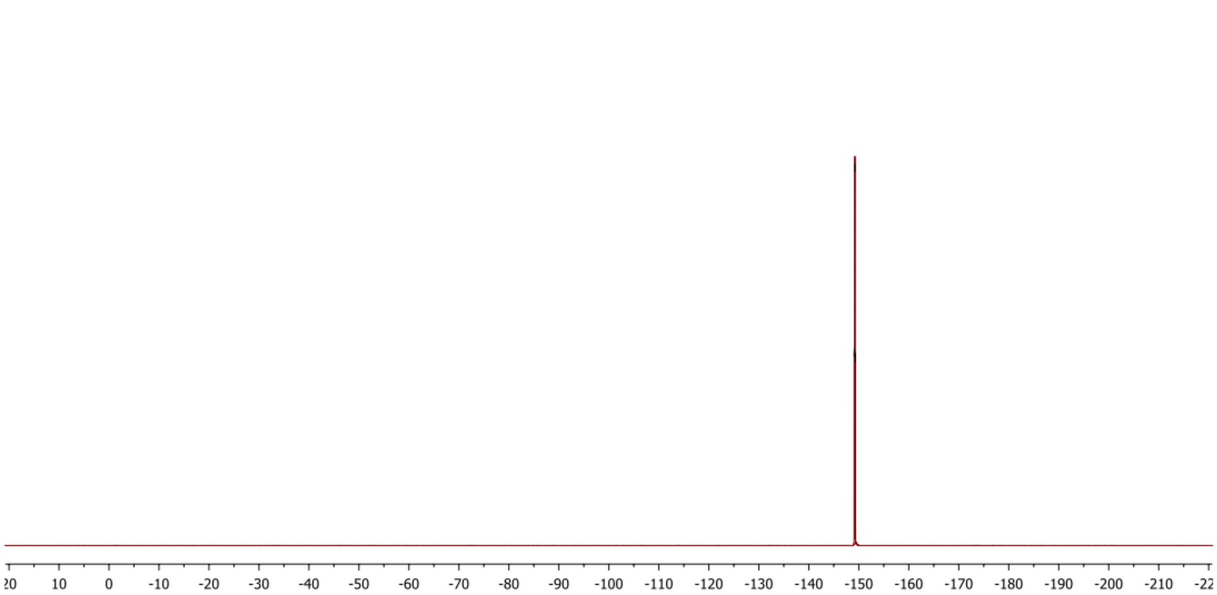
-149.10
-149.21
-149.33
20
10
0
-10
-20
-30
-40
-50
-60
-70
-80
-90
-100
-110
-120
-130
-140
-150
-160
-170
-180
-190
-200
-210
-22

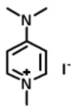

S9

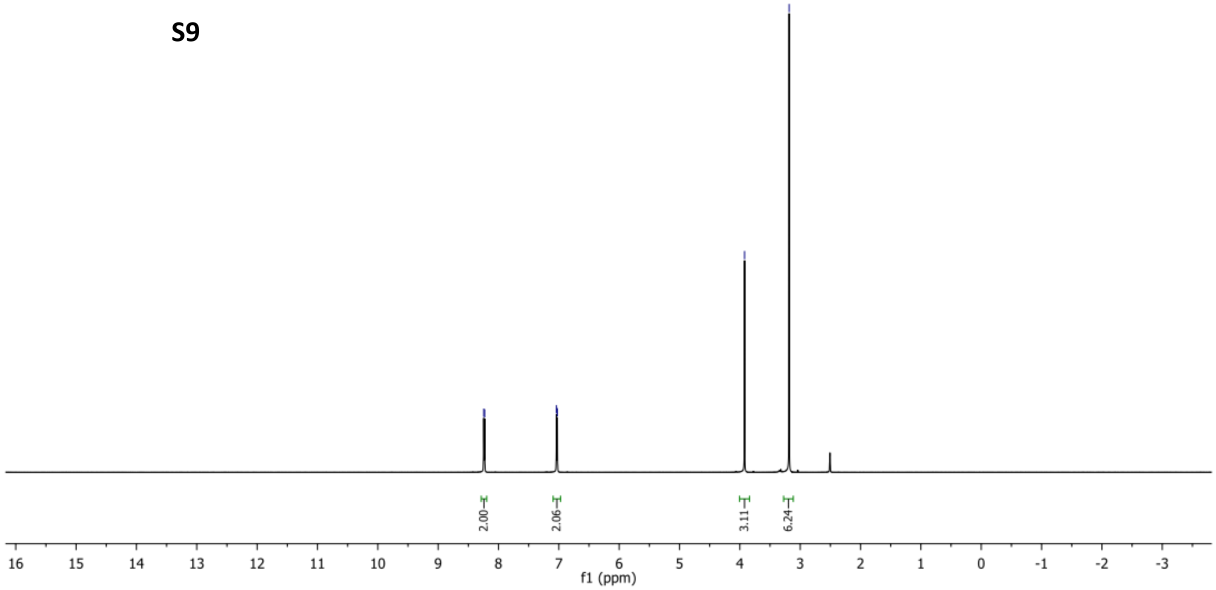

8.24
8.23
7.04
7.02
3.92
3.18
2.00
2.06
3.11
6.24
16
15
14
13
12
11
10
9
8
7
6
5
4
3
2
1
0
-1
-2
-3
f1 (ppm)

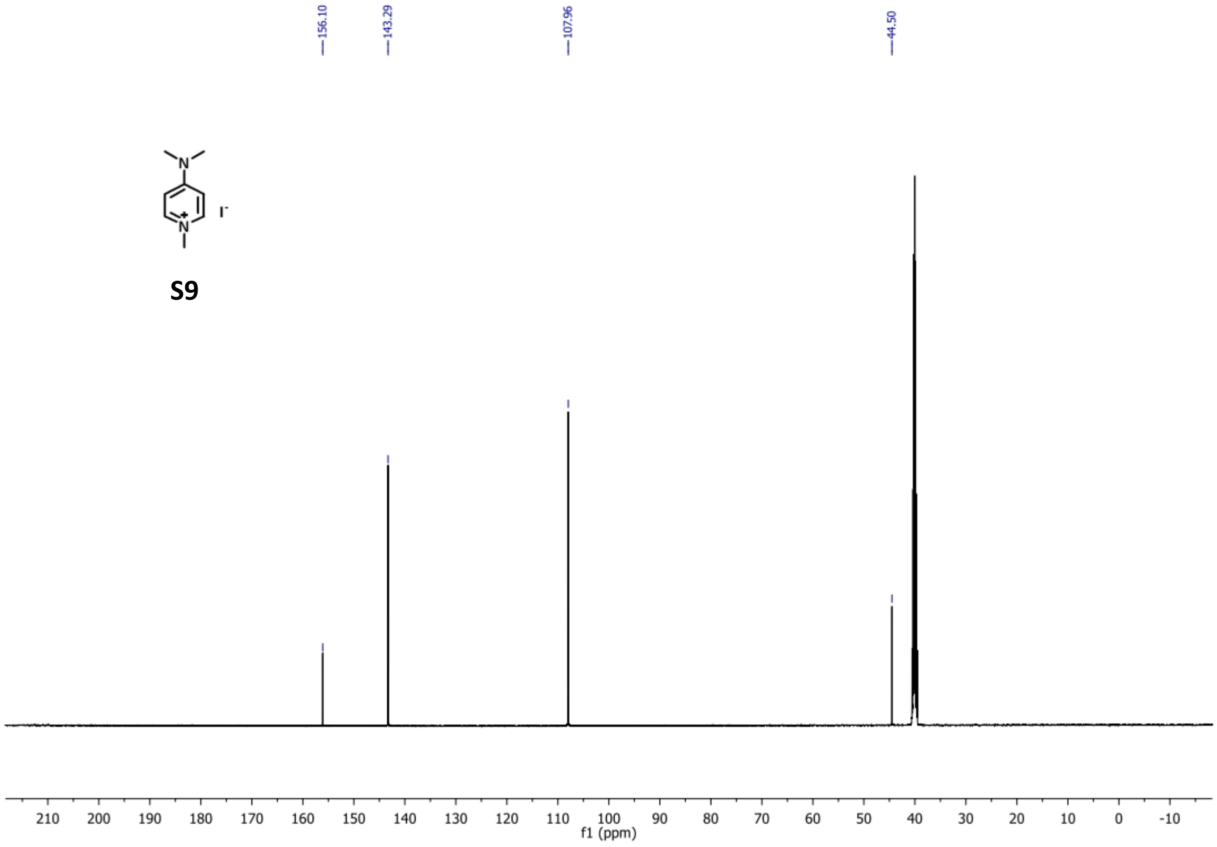
S9
156.10
143.29
107.96
44.50
210 200 190 180 170 160 150 140 130 120 110 100 90 80 70 60 50 40 30 20 10 0 -10
f1 (ppm)

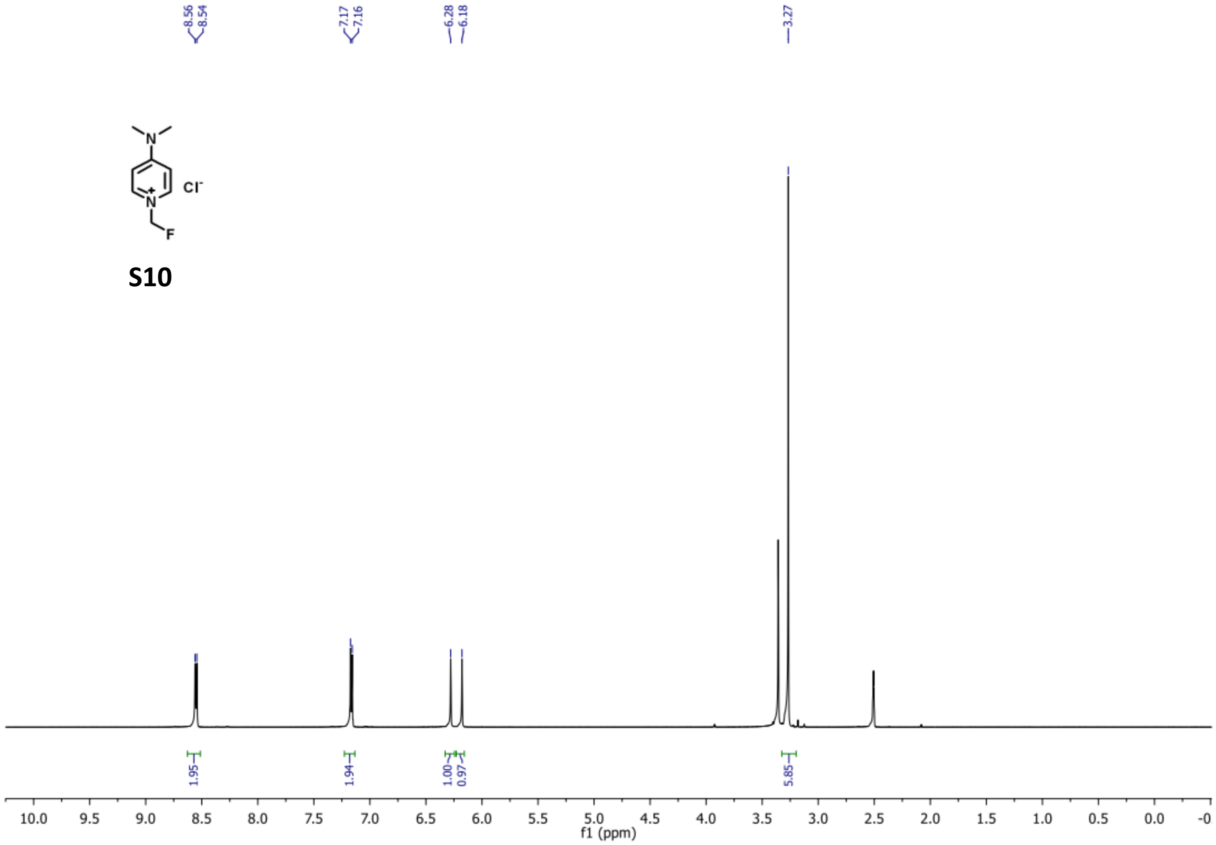
8.56
8.54
7.17
7.16
6.28
6.18
3.27
Cl⁻
S10
1.95
1.94
1.00
0.97
5.85
10.0 9.5 9.0 8.5 8.0 7.5 7.0 6.5 6.0 5.5 5.0 4.5 4.0 3.5 3.0 2.5 2.0 1.5 1.0 0.5 0.0 -0
f1 (ppm)

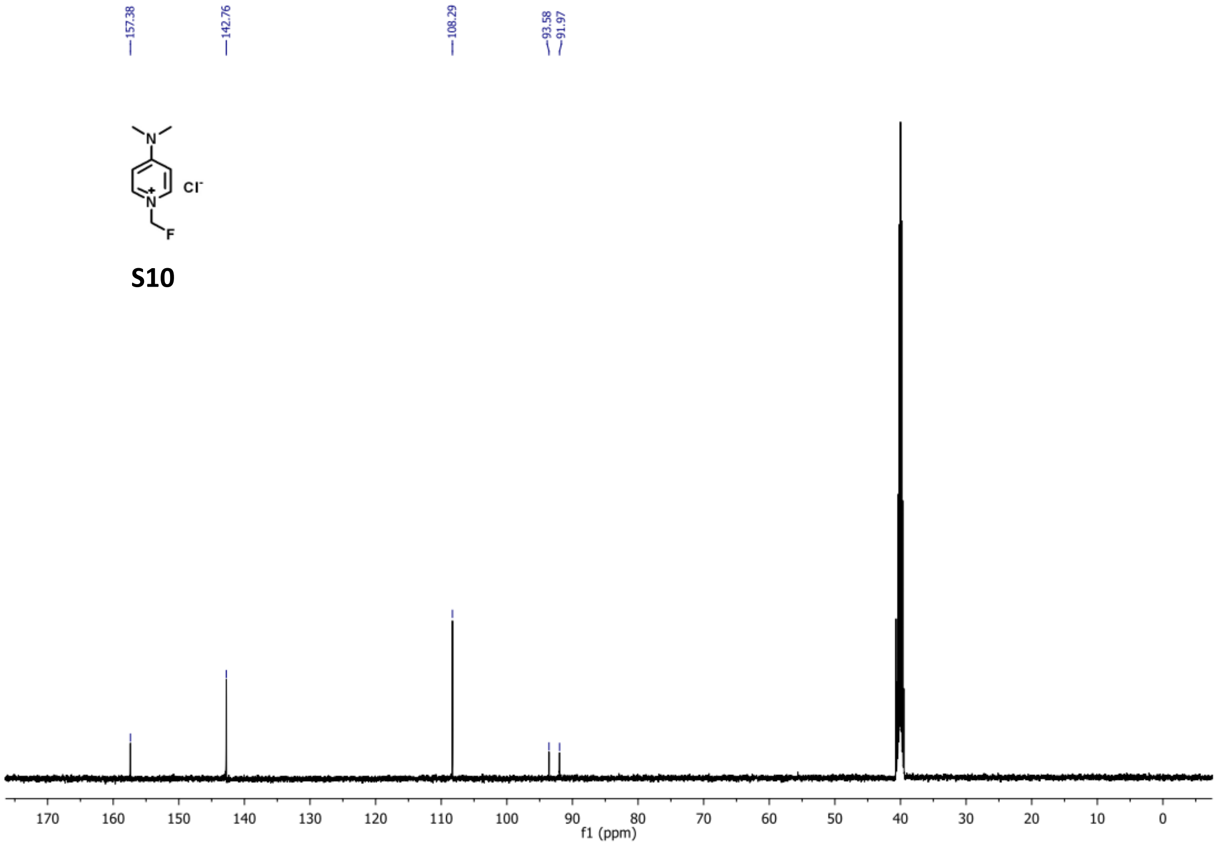
157.38
142.76
108.29
93.58
91.97
Cl-
170
160
150
140
130
120
110
100
90
80
70
60
50
40
30
20
10
0
f1 (ppm)

S10

S10

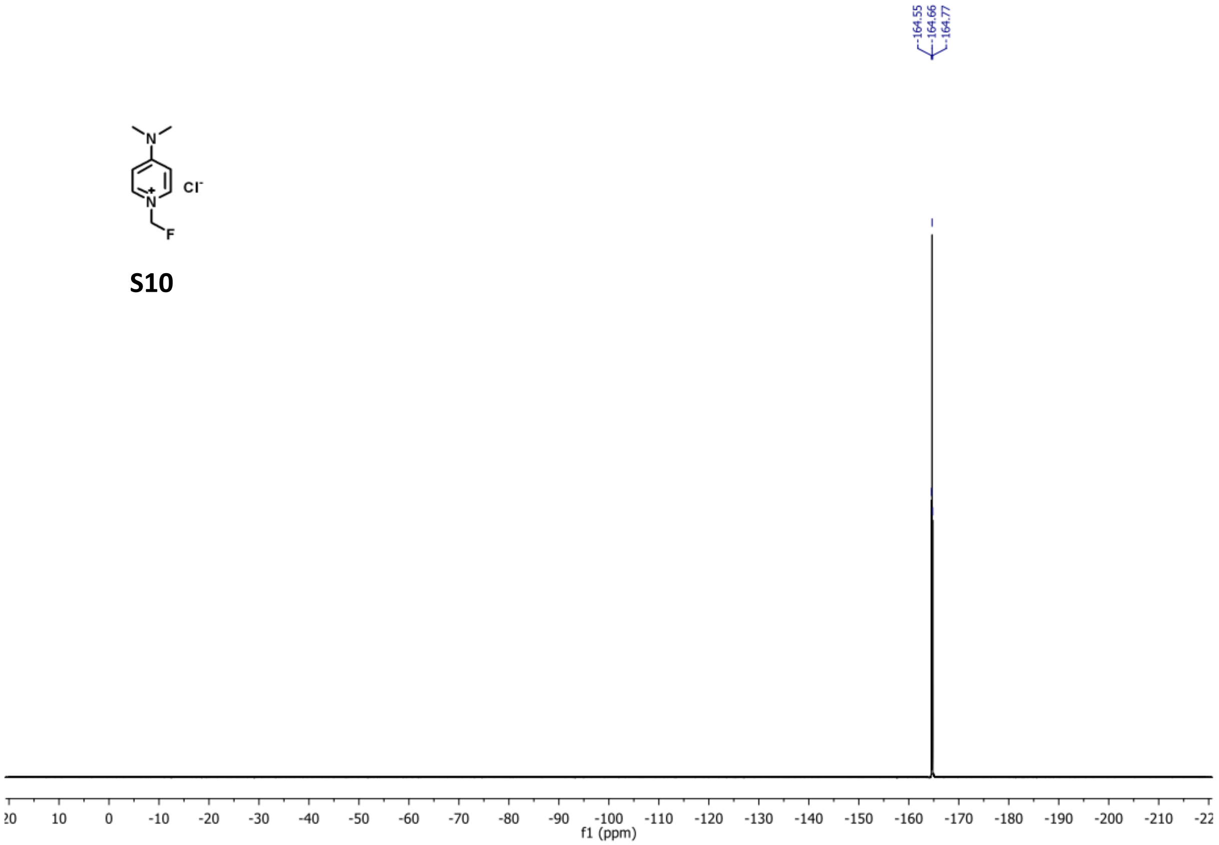
-164.55
-164.66
-164.77
20 10 0 -10 -20 -30 -40 -50 -60 -70 -80 -90 -100 -110 -120 -130 -140 -150 -160 -170 -180 -190 -200 -210 -22
f1 (ppm)

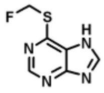
F
S
N
H
N
N
N

S11

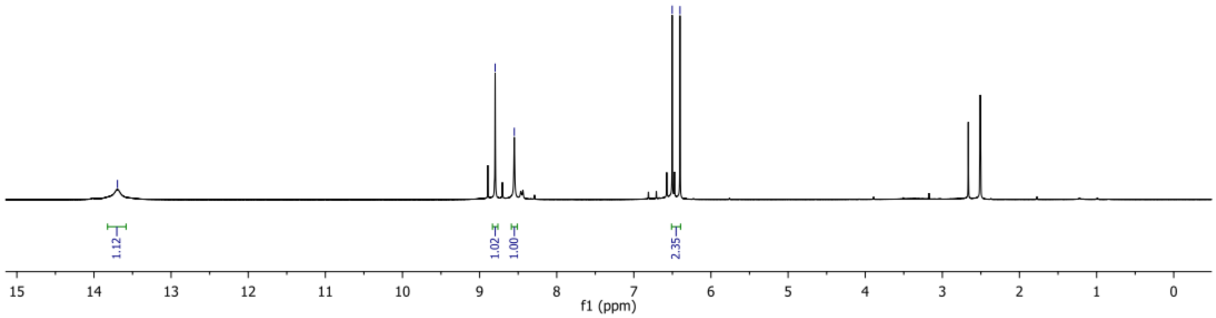
13.70
8.80
8.55
6.50
6.40
1.12
1.02
1.00
2.35
15 14 13 12 11 10 9 8 7 6 5 4 3 2 1 0
f1 (ppm)

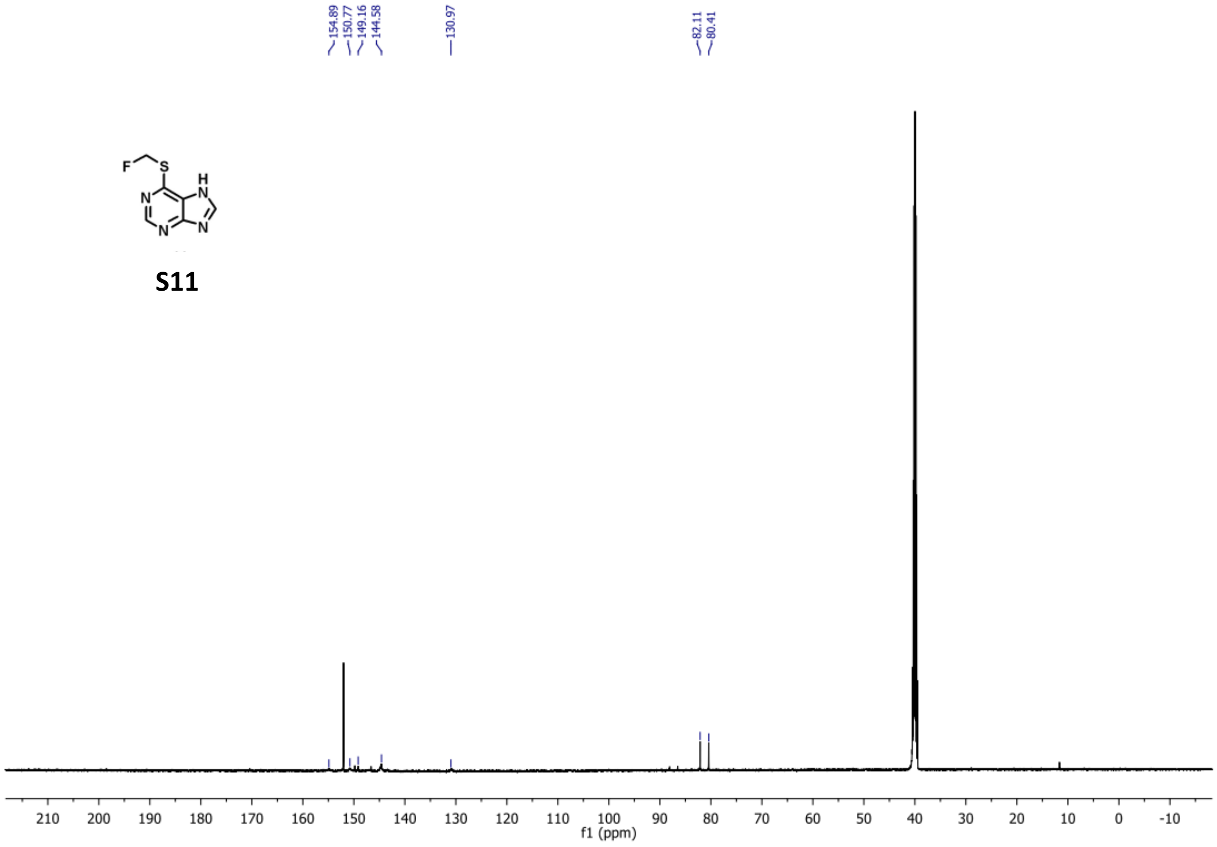
F
S
H
N
N
N
N
154.89
150.77
149.16
144.58
130.97
82.11
80.41
210
200
190
180
170
160
150
140
130
120
110
100
90
80
70
60
50
40
30
20
10
0
-10
f1 (ppm)

S11

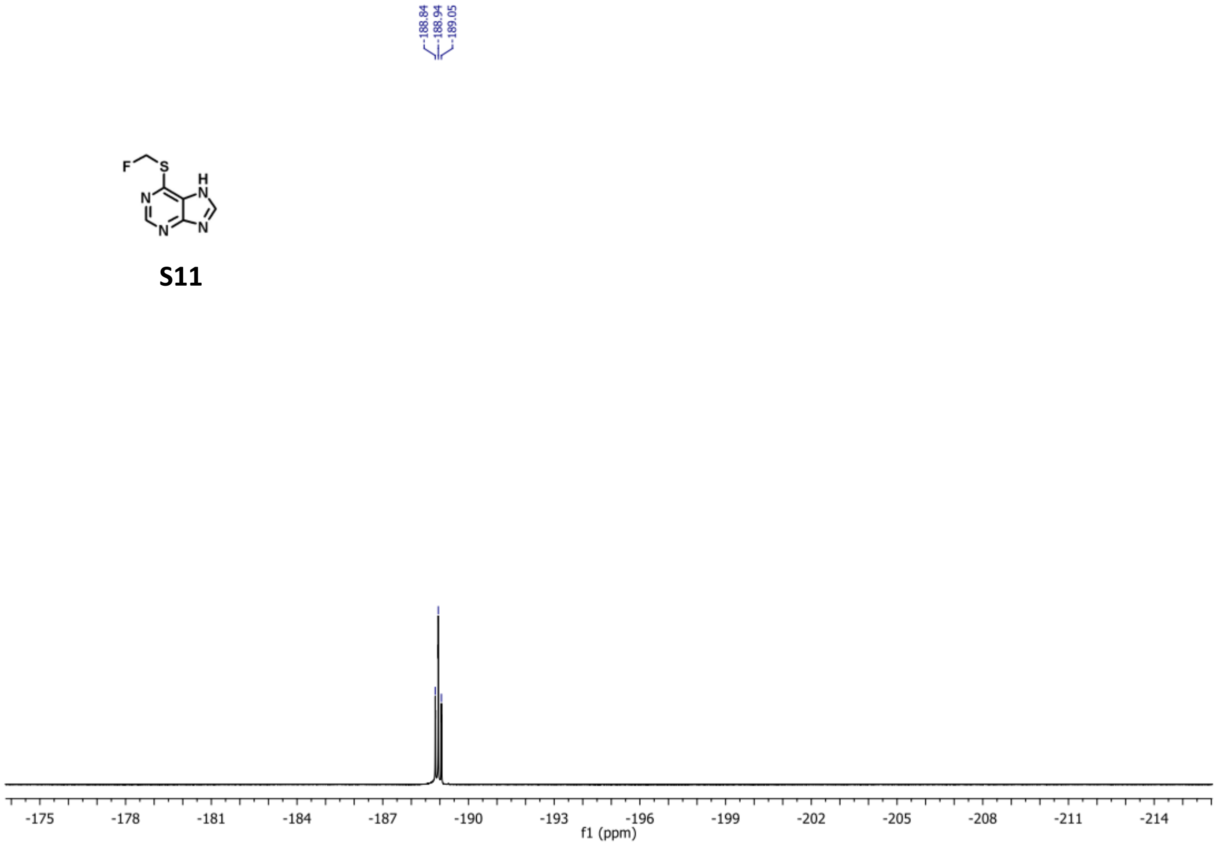
-188.84
-188.94
-189.05
F
S
H
N
N
N
N
S11
-175
-178
-181
-184
-187
-190
-193
-196
-199
-202
-205
-208
-211
-214
f1 (ppm)
